# Supplementary material for: Why do biting horseflies prefer warmer hosts? tabanids can escape easier from warmer targets
Source: PLoS One. 2020 May 13;15(5):e0233038. doi: 10.1371/journal.pone.0233038 (PMC7219777; doi:10.1371/journal.pone.0233038)
Supplement: S1 Table — : average, ±ΔT: standard deviation, Tmin: minimum, Tmax: maximum. (DOC) [file pone.0233038.s001.doc]

**S1 Table. Temperatures of black horses measured with thermography on shady and sunlit sides of the back and belly, and when the sun was occluded by clouds (cloudy).** <*T*>: average, ±Δ*T*: standard deviation, *T*_min_: minimum, *T*_max_: maximum.

| **black horses** | | | | | | | | | | | | |
| --- | --- | --- | --- | --- | --- | --- | --- | --- | --- | --- | --- | --- |
|  | | | | **back** | | | | | **belly** | | | |
| **No.** | **side** | **file name** | **<*T*>** | | **±Δ*T*** | ***T*_min_** | ***T*_max_** | **<*T*>** | | **±Δ*T*** | ***T*_min_** | ***T*_max_** |
| **1** | **shady** | AE070411 | 39.1 | | 1.7 | 31.7 | 43.1 | 35.6 | | 0.8 | 32.1 | 38.7 |
|  | **cloudy** | AF070401 | 37.4 | | 1.1 | 33.0 | 41.1 | 35.2 | | 0.8 | 32.3 | 38.1 |
|  | **sunlit** | AE070401 | 45.6 | | 2.3 | 31.3 | 51.9 | 38.7 | | 1.5 | 34.1 | 42.2 |
| **2** | **sunlit** | AA070400 | 40.8 | | 2.0 | 34.1 | 47.2 | 35.1 | | 0.9 | 30.9 | 38.4 |
|  |  | AA070402 | 44.2 | | 2.8 | 36.9 | 54.2 | - | | - | - | - |
|  |  | AA070403 | 43.4 | | 2.6 | 37.7 | 54.6 | 37.9 | | 0.8 | 35.6 | 41.1 |
|  |  | AD070403 | 40.3 | | 1.8 | 34.7 | 46.9 | - | | - | - | - |
|  |  | AD070404 | 38.6 | | 2.6 | 33.0 | 46.9 | 34.7 | | 0.7 | 32.7 | 37.1 |
