# Supplementary material for: Why do biting horseflies prefer warmer hosts? tabanids can escape easier from warmer targets
Source: PLoS One. 2020 May 13;15(5):e0233038. doi: 10.1371/journal.pone.0233038 (PMC7219777; doi:10.1371/journal.pone.0233038)
Supplement: S2 Table — : average, ±ΔT: standard deviation, Tmin: minimum, Tmax: maximum. (DOC) [file pone.0233038.s002.doc]

**S2 Table.** Temperatures of brown horses measured with thermography on shady and sunlit sides of the back and belly, and when the sun was occluded by clouds (cloudy). <*T*>: average, ±Δ*T*: standard deviation, *T*_min_: minimum, *T*_max_: maximum.

| **brown horses** | | | | | | | | | | |
| --- | --- | --- | --- | --- | --- | --- | --- | --- | --- | --- |
|  | | | **back** | | | | **belly** | | | |
| **No.** | **side** | **file name** | **<*T*>** | **±Δ*T*** | ***T*_min_** | ***T*_max_** | **<*T*>** | **±Δ*T*** | ***T*_min_** | ***T*_max_** |
| **1** | **shady** | AB70402 | 38.9 | 2.4 | 32.8 | 43.1 | 34.4 | 0.7 | 31.9 | 35.9 |
|  | **cloudy** | AE70413 | 35.8 | 1.0 | 31.8 | 39.6 | 34.5 | 0.4 | 31.7 | 36.0 |
|  | **sunlit** | AB070401 | 38.3 | 1.6 | 31.2 | 43.3 | 34.2 | 0.6 | 31.5 | 36.2 |
| **2** | **sunlit** | AE070416 | 40.1 | 1.4 | 35.7 | 44.6 | 36.2 | 1.0 | 34.1 | 38.9 |
