# Supplementary material for: Why do biting horseflies prefer warmer hosts? tabanids can escape easier from warmer targets
Source: PLoS One. 2020 May 13;15(5):e0233038. doi: 10.1371/journal.pone.0233038 (PMC7219777; doi:10.1371/journal.pone.0233038)
Supplement: S3 Table — : average, ±ΔT: standard deviation, Tmin: minimum, Tmax: maximum. (DOC) [file pone.0233038.s003.doc]

**S3 Table.** Temperatures of beige horses measured with thermography on shady and sunlit sides of the back and belly, and when the sun was occluded by clouds (cloudy). <*T*>: average, ±Δ*T*: standard deviation, *T*_min_: minimum, *T*_max_: maximum.

| **beige horses** | | | | | | | | | |
| --- | --- | --- | --- | --- | --- | --- | --- | --- | --- |
|  |  | **back** | | | | **belly** | | | |
| **side** | **file name** | **<*T*>** | **±Δ*T*** | ***T*_min_** | ***T*_max_** | **<*T*>** | **±Δ*T*** | ***T*_min_** | ***T*_max_** |
| **shady** | AE070408 | 40.7 | 1.9 | 35.6 | 45.4 | 36.1 | 0.5 | 34.2 | 38.4 |
| **cloudy** | AE070417 | 40.6 | 1.2 | 32.6 | 43.1 | 36.0 | 0.7 | 33.1 | 38.6 |
|  | AF070404 | 35.8 | 0.8 | 33.0 | 37.9 | 34.1 | 0.4 | 32.9 | 36.2 |
| **sunlit** | AE070405 | 41.7 | 2.0 | 37.8 | 46.2 | 36.7 | 0.9 | 35.0 | 39.4 |
