# Supplementary material for: Why do biting horseflies prefer warmer hosts? tabanids can escape easier from warmer targets
Source: PLoS One. 2020 May 13;15(5):e0233038. doi: 10.1371/journal.pone.0233038 (PMC7219777; doi:10.1371/journal.pone.0233038)
Supplement: S4 Table — : average, ±ΔT: standard deviation, Tmin: minimum, Tmax: maximum. (DOC) [file pone.0233038.s004.doc]

**Supplementary Table S4**: Temperatures of white horses measured with thermography on shady and sunlit sides of the back and belly, and when the sun was occluded by clouds (cloudy). <*T*>: average, ±Δ*T*: standard deviation, *T*_min_: minimum, *T*_max_: maximum.

| **white horses** | | | | | | | | | |
| --- | --- | --- | --- | --- | --- | --- | --- | --- | --- |
|  |  | **back** | | | | **belly** | | | |
| **side** | **file name** | **<*T*>** | **±Δ*T*** | ***T*_min_** | ***T*_max_** | **<*T*>** | **±Δ*T*** | ***T*_min_** | ***T*_max_** |
| **shady** | AA070405 | 34.8 | 0.9 | 32.4 | 37.4 | 35.7 | 0.5 | 34.0 | 37.6 |
|  | AC070401 | 34.3 | 0.9 | 31.5 | 36.5 | 35.0 | 0.5 | 33.2 | 36.6 |
| **cloudy** | AE070415 | 34.5 | 0.8 | 32.8 | 37.2 | 35.0 | 0.4 | 33.7 | 36.6 |
|  | AF070403 | 33.5 | 0.7 | 31.0 | 35.4 | 33.6 | 0.4 | 32.0 | 35.2 |
| **sunlit** | AE070403 | 39.3 | 1.6 | 32.5 | 46.6 | 36.5 | 0.5 | 34.7 | 38.1 |
|  | AE070410 | 39.5 | 1.2 | 33.9 | 44.0 | 37.1 | 0.7 | 35.5 | 39.4 |
|  | AH070404 | 36.1 | 0.9 | 33.3 | 39.4 | 34.9 | 0.4 | 33.1 | 35.9 |
|  | AH070405 | 36.1 | 1.6 | 31.6 | 40.6 | 33.9 | 0.6 | 32.4 | 36.2 |
