# Supplementary material for: Why do biting horseflies prefer warmer hosts? tabanids can escape easier from warmer targets
Source: PLoS One. 2020 May 13;15(5):e0233038. doi: 10.1371/journal.pone.0233038 (PMC7219777; doi:10.1371/journal.pone.0233038)
Supplement: S5 Table — (DOC) [file pone.0233038.s005.doc]

**Supplementary Table S5:** Results of χ^2^ tests comparing the sums of Supplementary Tables S6-S10 obtained in experiments 1-5.

| **experiment** | | **p** | **χ^2^** | **df** |
| --- | --- | --- | --- | --- |
| 1 | | p < 0.001 | 48.167 | 1 |
| 2 | | p = 0.5465 | 0.36364 | 1 |
| 3 | | p = 0.02781 | 4.84 | 1 |
| 4 | air filled | p < 0.001 | 41.953 | 1 |
|  | water filled | p = 0.02688 | 4.8983 | 1 |
| 5 | air filled, sunlit side | p < 0.001 | 58.182 | 1 |
|  | air filled, shady side | p = 0.1944 | 1.6842 | 1 |
|  | water filled, sunlit side | p = 0.3711 | 0.8 | 1 |
|  | water filled, shady side | p = 0.003276 | 8.6471 | 1 |
