# Supplementary material for: Why do biting horseflies prefer warmer hosts? tabanids can escape easier from warmer targets
Source: PLoS One. 2020 May 13;15(5):e0233038. doi: 10.1371/journal.pone.0233038 (PMC7219777; doi:10.1371/journal.pone.0233038)
Supplement: S6 Table — (DOC) [file pone.0233038.s006.doc]

**Supplementary Table S6**: Capture success (-: not captured, +: captured) of horseflies, and temperatures of the air (*T*_air_) and the surface of the air-filled sunlit barrel (*T*_barrel_) in experiment 1 on 1 July 2019.

| **1 July 2019**  **time (UTC + 2 h)** | ***T*_air_ (^o^C)** | ***T*_barrel_ (^o^C)** | **capture**  **success** |
| --- | --- | --- | --- |
| 10:20 | 28 | 50 | 2-, 2+ |
| 10:30 | 29 | 49 | 4-, 2+ |
| 10:45 | 30 | 45 | 6-, 2+ |
| 11:00 | 30 | 46 | 6-, 2+ |
| 11:20 | 31 | 48 | 6- |
| 11:40 | 32 | 46 | 4-, 4+ |
| 12:00 | 33 | 45 | 8- |
| 12:20 | 35 | 54 | 2- |
| 12:40 | 36 | 50 | 2- |
| 13:00 | 37 | 54 | 2- |
| 13:20 | 37 | 52 | 2- |
| 13:40 | 38 | 52 | 2- |
| 14:00 | 38 | 50 | 6- |
| 14:20 | 38 | 52 | 4- |
| 14:40 | 37 | 54 | 2- |
| 15:00 | 37 | 52 | 8- |
| 15:20 | 34 | 55 | 8- |
| 15:40 | 33 | 60 | 2- |
| 16:00 | 33 | 62 | 2- |
| 16:20 | 32 | 32 (cloudy) | 2+ |
| 16:40 | 32 | 61 | 2- |
| 17:00 | 31 | 61 | 2- |
|  |  | **sum** | **96 =**  **82- (85.4 %)**  **14+ (14.6 %)** |
