# Supplementary material for: Why do biting horseflies prefer warmer hosts? tabanids can escape easier from warmer targets
Source: PLoS One. 2020 May 13;15(5):e0233038. doi: 10.1371/journal.pone.0233038 (PMC7219777; doi:10.1371/journal.pone.0233038)
Supplement: S7 Table — (DOC) [file pone.0233038.s007.doc]

**Supplementary Table S7**: Capture success (-: not captured, +: captured)) of horseflies, and temperatures of the air (*T*_air_) and the surface of the air-filled shady barrel (*T*_barrel_) in experiment 2 on 2 July 2019.

| **2 July 2019**  **time (UTC + 2 h)** | ***T*_air_ (^o^C)** | ***T*_barrel_ (^o^C)** | **capture**  **success** |
| --- | --- | --- | --- |
| 9:40 | 27 | 27 | 2+ |
| 10:00 | 27 | 28 | 1+ |
| 10:20 | 28 | 28 | 1-, 2+ |
| 10:40 | 28 | 29 | 1-, 2+ |
| 11:00 | 28 | 29 | 2+ |
| 11:20 | 29 | 30 | 1- |
| 11:40 | 29 | 30 | 3-, 1+ |
| 12:00 | 30 | 30 | 2- |
| 12:20 | 30 | 30 | 2- |
| 12:40 | 30 | 31 | 1- |
| 13:00 | 30 | 31 | 3-, 2+ |
| 13:20 | 30 | 31 | 3-, 2+ |
| 13:40 | 31 | 31 | 1- |
| 14:00 | 31 | 31 | 1- |
| 14:20 | 31 | 32 | 2-, 1+ |
| 14:40 | 31 | 32 | 1-, 3+ |
| 15:00 | 31 | 32 | 1+ |
| 15:20 | 30 | 31 | 1- |
| 15:40 | 30 | 31 | 1+ |
| 16:00 | 30 | 31 | 1- |
|  |  | **sum** | **44 =**  **24- (54.5 %)**  **20+ (45.5 %)** |
