# Supplementary material for: Why do biting horseflies prefer warmer hosts? tabanids can escape easier from warmer targets
Source: PLoS One. 2020 May 13;15(5):e0233038. doi: 10.1371/journal.pone.0233038 (PMC7219777; doi:10.1371/journal.pone.0233038)
Supplement: S9 Table — (DOC) [file pone.0233038.s009.doc]

**Supplementary Table S9**: Capture success (-: not captured, +: captured) of horseflies, and temperatures of the air (*T*_air_) and the sunlit side of the surface of the air-filled warm barrel (*T*_warm_) and the water-filled cold barrel (*T*_cold_) in experiment 4 on 4 July 2019.

| **4 July 2019**  **time (UTC + 2 h)** | ***T*_air_ (^o^C)** | ***T*_warm_ (^o^C) / success** | ***T*_cold_ (^o^C) / success** |
| --- | --- | --- | --- |
| 10:00 | 23 | 37 ^o^C / 3-, 3+ | 19 ^o^C / 2+, 1- |
| 10:16 | 23 | 39 ^o^C / 5-, 7+ | 19 ^o^C / 3+ |
| 10:20 | 23 | 41 ^o^C / 11-, 4+ | 19 ^o^C / 2+, 3- |
| 10:25 | 23 | 42 ^o^C / 8-, 2+ | 19 ^o^C / 3+, 1- |
| 10:30 | 24 | 42 ^o^C / 10-, 1+ | 19 ^o^C / 1+, 1- |
| 10:35 | 24 | 42 ^o^C / 5-, 1+ | 19 ^o^C / 2+, 1- |
| 10:40 | 24 | 41 ^o^C / 6- | 19 ^o^C / 2+, 1- |
| 10:50 | 24 | 40 ^o^C / 6-, 1+ | 19 ^o^C / 3+, 1- |
| 11:00 | 24 | 40 ^o^C / 6- | 20 ^o^C / 2+, 1- |
| 11:15 | 24 | 39 ^o^C / 7-, 1+ | 20 ^o^C / 3+, 2- |
| 11:17 | 24 | 38 ^o^C / 5- | 20 ^o^C / 3+, 2- |
| 11:30 | 25 | 38 ^o^C / 4- | 20 ^o^C / 5+, 2- |
| 11:55 | 25 | 37 ^o^C / 5- | 20 ^o^C / 4+, 3- |
| 12:00 | 25 | 37 ^o^C / 6- | 20 ^o^C / 3+, 2- |
| **sum** |  | **107 =**  **87- (81.3 %)**  **20+ (18.7 %)** | **59 =**  **21- (35.6 %)**  **38+ (64.4 %)** |
