# Supplementary material for: Why do biting horseflies prefer warmer hosts? tabanids can escape easier from warmer targets
Source: PLoS One. 2020 May 13;15(5):e0233038. doi: 10.1371/journal.pone.0233038 (PMC7219777; doi:10.1371/journal.pone.0233038)
Supplement: S10 Table — (DOC) [file pone.0233038.s010.doc]

**Supplementary Table S10**: Capture success (-: not captured, +: captured) of horseflies, and temperatures of the air (*T*_air_) and the surface of the air-filled sunlit barrel (*T*_warm_) and the cold-water-filled sunlit barrel (*T*_cold_) in experiment 5 on 11 July 2019.

|  |  | **sunlit air-filled barrel**  ***T* (^o^C) / success** | | **sunlit water-filled barrel**  ***T* (^o^C) / success** | |
| --- | --- | --- | --- | --- | --- |
| **11 July 2019**  **time (UTC + 2 h)** | ***T*_air_ (^o^C)** | **sunlit side** | **shady side** | **sunlit side** | **shady side** |
| 10:20 | 23 | 41 ^o^C / 11- | 25 ^o^C / 4+ | 24 ^o^C / 1-, 8+ | 21 ^o^C / 6+ |
| 11:00 | 25 | 39 ^o^C / 10-, 1+ | 27 ^o^C / 6+ | 26 ^o^C / 3-, 7+ | 22 ^o^C / 4+ |
| 11:30 | 27 | 38 ^o^C / 14-, 2+ | 28 ^o^C / 5+ | 27 ^o^C / 3-, 6+ | 22 ^o^C / 3+ |
| 12:00 | 28 | 38 ^o^C / 9-, 2+ | 28 ^o^C / 4- | 28 ^o^C / 5-, 5+ | 23 ^o^C / 1-, 2+ |
| 12:30 | 29 | 38 ^o^C / 1-, 4+ | 31 ^o^C / 3- | 29 ^o^C / 6-, 4+ | 23 ^o^C / 1-, 4+ |
| 13:00 | 28 | 38 ^o^C / 15-, 3+ | 30 ^o^C / 5- | 29 ^o^C / 7-, 2+  ice pack  refreshment | 24 ^o^C / 1-, 2+  ice pack  refreshment |
| 13:30 | 28 | 39 ^o^C / 3-, 2+ | 31 ^o^C / 2- | 27 ^o^C / 1-, 4+ | 23 ^o^C / 1-, 5+ |
| 14:00 | 27 | 40 ^o^C / 7-, 1+ | 31 ^o^C / 1- | 27 ^o^C / 1-, 3+ | 23 ^o^C / 2-, 3+ |
| 14:30 | 28 | 41 ^o^C / 6- | 30 ^o^C / 2+ | 28 ^o^C / 2-, 2+ | 25 ^o^C / 3-, 2+ |
| 15:00 | 29 | 41 ^o^C / 4- | 30 ^o^C / 3+ | 28 ^o^C / 1-, 1+ | 25 ^o^C / 2-, 2+ |
| 15:30 | 28 | 41 ^o^C / 9- | 31 ^o^C / 2+ | 29 ^o^C / 3-, 1+ | 26 ^o^C / 2-, 1+ |
| 16:00 | 27 | 42 ^o^C / 6- | 31 ^o^C / 1+ | 29 ^o^C / 3-, 1+ | 27 ^o^C / 2-, 2+ |
| **sum** |  | **110 =**  **95- (86.4 %)**  **15+ (13.6 %)** | **38 =**  **15- (39.5 %)**  **23+ (60.5 %)** | **80 =**  **36- (45 %)**  **44+ (55 %)** | **51 =**  **15- (29.4 %)**  **36+ (70.6 %)** |
