# Supplementary material for: Why do biting horseflies prefer warmer hosts? tabanids can escape easier from warmer targets
Source: PLoS One. 2020 May 13;15(5):e0233038. doi: 10.1371/journal.pone.0233038 (PMC7219777; doi:10.1371/journal.pone.0233038)
Supplement: S11 Table — The escape probability ε of tabanids depends highly significantly on the barrel surface temperature Tbarrel in the interval 17°C ≤ Tbarrel ≤ 62°C. The large difference between the null deviance and the residual deviance suggests that the logistic regression model is accurate. (DOC) [file pone.0233038.s011.doc]

**Supplementary Table S11.** Summary of the logistic regression. The escape probability ε of tabanids depends highly significantly on the barrel’s surface temperature *T*_barrel_ in the interval 17 °C ≤ *T*_barrel_ ≤ 62 °C. The large difference between the null deviance and the residual deviance suggests that the logistic regression model is accurate.

| **coefficients** | **estimate** | **standard error** | **z** | **p** |
| --- | --- | --- | --- | --- |
| intercept | -3.67169 | 0.37845 | -9.702 | < 0.0001 |
| 17 °C ≤ *T*_barrel_ ≤ 62 °C | 0.12854 | 0.01168 | 11.005 | < 0.0001 |
|  | | | | |
| **null deviance** | **df** |  | **residual deviance** | **df** |
| 807.27 | 610 |  | 640.20 | 609 |
