# Supplementary material for: Why do biting horseflies prefer warmer hosts? tabanids can escape easier from warmer targets
Source: PLoS One. 2020 May 13;15(5):e0233038. doi: 10.1371/journal.pone.0233038 (PMC7219777; doi:10.1371/journal.pone.0233038)
Supplement: S12 Table — The escape probability ε of tabanids depends highly significantly on the barrel surface temperature Tbarrel in the interval 31°C ≤ Tbarrel ≤ 55°C. (DOC) [file pone.0233038.s012.doc]

**Supplementary Table S12.** Summary of the logistic regression. The escape probability ε of tabanids depends highly significantly on the barrel’s surface temperature *T*_barrel_ in the interval 31 °C ≤ *T*_barrel_ ≤ 55 °C.

| **coefficients** | **estimate** | **standard error** | **z** | **p** |
| --- | --- | --- | --- | --- |
| intercept | -2.58837 | 1.13754 | -2.275 | 0.022881 |
| 31 °C ≤ *T*_barrel_ ≤ 55 °C | 0.10173 | 0.02871 | 3.544 | 0.000395 |
|  | | | | |
| **null deviance** | **df** |  | **residual deviance** | **df** |
| 322.96 | 339 |  | 308.74 | 338 |
