# Supplementary material for: Why do biting horseflies prefer warmer hosts? tabanids can escape easier from warmer targets
Source: PLoS One. 2020 May 13;15(5):e0233038. doi: 10.1371/journal.pone.0233038 (PMC7219777; doi:10.1371/journal.pone.0233038)
Supplement: S13 Table — The escape probability ε of tabanids depends highly significantly on the air temperature Tair. (DOC) [file pone.0233038.s013.doc]

**Supplementary Table S13.** Summary of the logistic regression. The escape probability ε of tabanids depends highly significantly on the air temperature *T*_air_.

| **coefficients** | **estimate** | **standard error** | **z** | **p** |
| --- | --- | --- | --- | --- |
| intercept | -3.02192 | 0.73835 | -4.093 | < 0.0001 |
| *T*_air_ | 0.13073 | 0.02735 | 4.781 | < 0.0001 |
|  | | | | |
| **null deviance** | **df** |  | **residual deviance** | **df** |
| 806.34 | 609 |  | 780.69 | 608 |
