# Supplementary material for: Why do biting horseflies prefer warmer hosts? tabanids can escape easier from warmer targets
Source: PLoS One. 2020 May 13;15(5):e0233038. doi: 10.1371/journal.pone.0233038 (PMC7219777; doi:10.1371/journal.pone.0233038)
Supplement: S14 Table — The escape probability ε of tabanids depends highly significantly on the temperature difference Tbarrel—Tair. (DOC) [file pone.0233038.s014.doc]

**Supplementary Table S14.** Summary of the logistic regression. The escape probability ε of tabanids depends highly significantly on the temperature difference *T*_barrel_ - *T*_air_.

| **coefficients** | **estimate** | **standard error** | **z** | **p** |
| --- | --- | --- | --- | --- |
| intercept | -0.18672 | 0.10793 | -1.73 | 0.0836 |
| *T*_barrel_ - *T*_air_ | 0.12648 | 0.01181 | 10.71 | < 0.0001 |
|  | | | | |
| **null deviance** | **df** |  | **residual deviance** | **df** |
| 806.34 | 609 |  | 660.43 | 608 |
