# Supplementary material for: Why do biting horseflies prefer warmer hosts? tabanids can escape easier from warmer targets
Source: PLoS One. 2020 May 13;15(5):e0233038. doi: 10.1371/journal.pone.0233038 (PMC7219777; doi:10.1371/journal.pone.0233038)

**S1 Fig.** **Photographs, thermograms and thermograms with selected back and belly areas of black horses under different illumination conditions.** shady: shady side of the sunlit horse. sunlit: sunlit side of the sunlit horse. cloudy: illuminated by skylight when the sun was occluded by clouds.


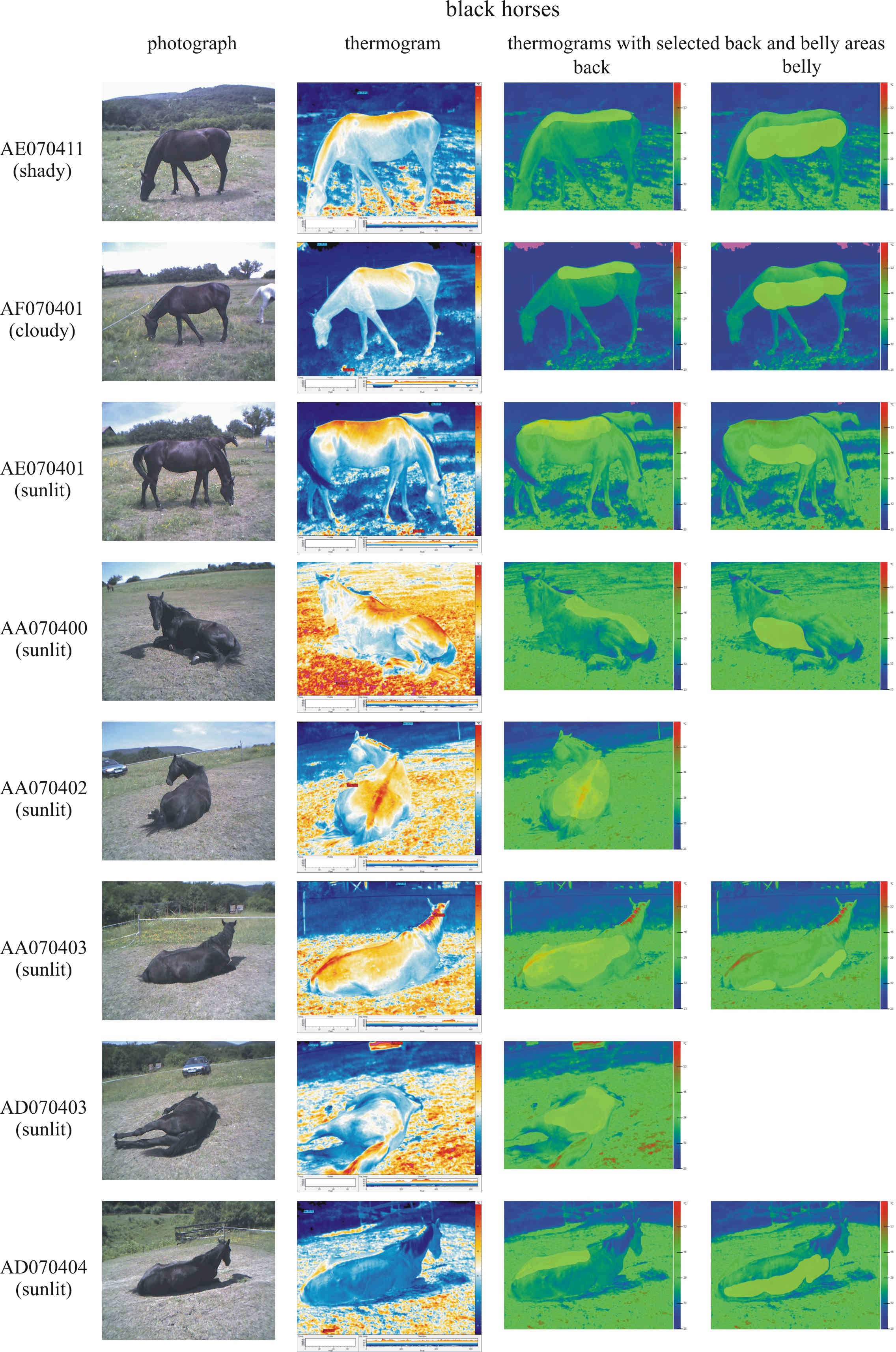

Supplement: S1 Fig — Shaded: shaded side of the sunlit horse. sunlit: sunlit side of the sunlit horse. cloudy: illuminated by skylight when the sun was occluded by clouds. (DOC) [file pone.0233038.s015.doc]
