# Supplementary material for: Why do biting horseflies prefer warmer hosts? tabanids can escape easier from warmer targets
Source: PLoS One. 2020 May 13;15(5):e0233038. doi: 10.1371/journal.pone.0233038 (PMC7219777; doi:10.1371/journal.pone.0233038)
Supplement: Attachment — Submitted filename: PONE-D-20-04024_R1 editor feedback.pdf [file pone.0233038.s020.pdf]

PLOS ONE

Why Do Biting Horseflies Prefer Warmer Hosts? Tabanids can Escape Easier from Warmer Targets

--Manuscript Draft--

**Manuscript Number:**	PONE-D-20-04024R1
**Article Type:**	Research Article
**Full Title:**	Why Do Biting Horseflies Prefer Warmer Hosts? Tabanids can Escape Easier from Warmer Targets
**Short Title:**	Horseflies versus cold/warm horses
**Corresponding Author:**	Gabor Horvath, D. Sc.<br>Eötvös University<br>Budapest, HUNGARY
**Keywords:**	Tabanidae; horsefly; thermoreception; warmth preference; host animal; parasite-host interaction
**Abstract:**	Blood-sucking horseflies (tabanids) prefer warmer (sunlit, darker) host animals and generally attack them in sunshine, the reason for which was unknown until now. Recently, it was hypothesized that blood-seeking female tabanids prefer elevated temperatures, because their wing muscles are quicker and their nervous system functions better at a warmer body temperature brought about by warmer microclimate, and thus they can more successfully avoid the host's parasite-repelling reactions by prompt takeoffs. To test this hypothesis, we studied in field experiments the success rate of escape reactions of tabanids landed on black targets as a function of the target temperature, and measured the surface temperature of differently coloured horses with thermography. We found that the escape success of tabanids decreased with decreasing target temperature, that is escape success is driven by temperature. Our results explain the behaviour of biting horseflies that they prefer warmer hosts against colder ones. Since in sunshine the darker the host the warmer its body surface, our results also explain why horseflies prefer sunlit dark (brown, black) hosts against bright (beige, white) ones, and why these parasites attack their hosts usually in sunshine, rather than under shady conditions.
**Order of Authors:**	Gabor Horvath
	Ádám Pereszlényi
	Ádám Egri
	Tímea Tóth
	Imre M. Jánosi
**Opposed Reviewers:**	Timothy CARO<br>University of California Davis<br>tmcaro@ucdavis.edu<br>Since Timothy CARO (Department of Wildlife, Fish and Conservation Biology, University of California, USA, e-mail: tmcaro@ucdavis.edu) is a serious rival of our group in the research topic of horse-horsefly interaction, he is an unpreferred referee of our submitted manuscript.
**Response to Reviewers:**	All specific reviewer and editor comments are responded in detail in the following uploaded file:<br><br>+TabanidThermalTrapping_PLoS-One-response.doc
**Additional Information:**	
**Question**	**Response**
**Financial Disclosure**<br><br>Enter a financial disclosure statement that	This work was supported by the grant NKFIH K-123930 (Experimental Study of the Functions of Zebra Stripes: A New Thermophysiological Explanation) received by Gábor Horváth from the Hungarian National Research, Development and Innovation

describes the sources of funding for the work included in this submission. Review the submission guidelines for detailed requirements. View published research articles from *PLOS ONE* for specific examples.<br><br>This statement is required for submission and **will appear in the published article** if the submission is accepted. Please make sure it is accurate.<br><br>**Unfunded studies**<br>Enter: *The author(s) received no specific funding for this work.*<br><br>**Funded studies**<br>Enter a statement with the following details:<br>• Initials of the authors who received each award<br>• Grant numbers awarded to each author<br>• The full name of each funder<br>• URL of each funder website<br>• Did the sponsors or funders play any role in the study design, data collection and analysis, decision to publish, or preparation of the manuscript?<br>• **NO** - Include this sentence at the end of your statement: *The funders had no role in study design, data collection and analysis, decision to publish, or preparation of the manuscript.*<br>• **YES** - Specify the role(s) played.<br><br>* typeset	Office. Ádám Egri was supported by the Economic Development and Innovation Operational Programme (GINOP-2.3.2-15-2016-00057), the grant NKFIH PD-131738 and the János Bolyai Research Scholarship of the Hungarian Academy of Sciences. The funders had no role in study design, data collection and analysis, decision to publish, or preparation of the manuscript.
**Competing Interests**<br><br>Use the instructions below to enter a competing interest statement for this submission. On behalf of all authors, disclose any competing interests that could be perceived to bias this work—acknowledging all financial support and any other relevant financial or non-financial competing interests.<br><br>This statement **will appear in the published article** if the submission is accepted. Please make sure it is accurate. View published research articles from *PLOS ONE* for specific examples.	The authors have declared that no competing interests exist.

**NO authors have competing interests**<br><br>Enter: *The authors have declared that no competing interests exist.*<br><br>**Authors with competing interests**<br><br>Enter competing interest details beginning with this statement:<br><br>*I have read the journal's policy and the authors of this manuscript have the following competing interests: [insert competing interests here]*<br><br>* typeset	
**Ethics Statement**<br><br>Enter an ethics statement for this submission. This statement is required if the study involved:<br><br>• Human participants<br>• Human specimens or tissue<br>• Vertebrate animals or cephalopods<br>• Vertebrate embryos or tissues<br>• Field research<br><br>Write "N/A" if the submission does not require an ethics statement.<br><br>General guidance is provided below. Consult the submission guidelines for detailed instructions. **Make sure that all information entered here is included in the Methods section of the manuscript.**	For the location and activities of our field study no specific permissions were required.<br><br>Csaba Viski permitted us to photograph his horses in his horse farm in Szokolya.

**Format for specific study types**<br><br>**Human Subject Research (involving human participants and/or tissue)**<br>• Give the name of the institutional review board or ethics committee that approved the study<br>• Include the approval number and/or a statement indicating approval of this research<br>• Indicate the form of consent obtained (written/oral) or the reason that consent was not obtained (e.g. the data were analyzed anonymously)<br><br>**Animal Research (involving vertebrate animals, embryos or tissues)**<br>• Provide the name of the Institutional Animal Care and Use Committee (IACUC) or other relevant ethics board that reviewed the study protocol, and indicate whether they approved this research or granted a formal waiver of ethical approval<br>• Include an approval number if one was obtained<br>• If the study involved *non-human primates*, add *additional details* about animal welfare and steps taken to ameliorate suffering<br>• If anesthesia, euthanasia, or any kind of animal sacrifice is part of the study, include briefly which substances and/or methods were applied<br><br>**Field Research**<br><br>Include the following details if this study involves the collection of plant, animal, or other materials from a natural setting:<br>• Field permit number<br>• Name of the institution or relevant body that granted permission	
**Data Availability**<br><br>Authors are required to make all data underlying the findings described fully available, without restriction, and from the time of publication. PLOS allows rare exceptions to address legal and ethical concerns. See the PLOS Data Policy and FAQ for detailed information.	Yes - all data are fully available without restriction

A Data Availability Statement describing where the data can be found is required at submission. Your answers to this question constitute the Data Availability Statement and **will be published in the article**, if accepted.<br><br>**Important:** Stating 'data available on request from the author' is not sufficient. If your data are only available upon request, select 'No' for the first question and explain your exceptional situation in the text box.<br><br>Do the authors confirm that all data underlying the findings described in their manuscript are fully available without restriction?	
**Describe where the data may be found in full sentences. If you are copying our sample text, replace any instances of XXX with the appropriate details.**<br><br>• If the data are **held or will be held in a public repository**, include URLs, accession numbers or DOIs. If this information will only be available after acceptance, indicate this by ticking the box below. For example: *All XXX files are available from the XXX database (accession number(s) XXX, XXX.).*<br>• If the data are all contained **within the manuscript and/or Supporting Information files**, enter the following: *All relevant data are within the manuscript and its Supporting Information files.*<br>• If neither of these applies but you are able to provide **details of access elsewhere**, with or without limitations, please do so. For example:<br><br>*Data cannot be shared publicly because of [XXX]. Data are available from the XXX Institutional Data Access / Ethics Committee (contact via XXX) for researchers who meet the criteria for access to confidential data.*<br><br>*The data underlying the results presented in the study are available from (include the name of the third party*	All relevant data are within the manuscript and its Supporting Information file.

Powered by Editorial Manager® and ProduXion Manager® from Aries Systems Corporation

and contact information or URL).<br>• This text is appropriate if the data are owned by a third party and authors do not have permission to share the data.<br><br>* typeset	
Additional data availability information:	

Dear Editorial Office of *PLoS One*
and
Dr. Heike Lutermann (Academic Editor)

thank you for your letter of 16 March 2020 in which you sent me three reviews about our paper PONE-D-20-04024 entitled

Why Do Biting Horseflies Prefer Warmer Hosts?
Tabanids can Escape Easier from Warmer Targets

by Gabor Horvath, Adam Pereszlenyi, Adam Egri, Timea Toth and Imre M. Janosi

We have revised our paper on the basis of the three reviews. All changes done with respect to the reviews of Referee 1, Referee 2 and Referee 3 are marked with blue, green and orange, respectively. Below you can read our Point-by-Point Responses to the points of all three Reviewers.

On behalf of my co-authors, I would like to submit our revised paper to *PLoS One*.

Sincerely yours: Gábor Horváth (corresponding author)

Prof. GABOR HORVATH
Environmental Optics Laboratory
Department of Biological Physics
Eotvos University
H-1117 Budapest, Pazmany setany 1, Hungary
e-mail: gh@arago.elte.hu
https://arago.elte.hu

Manuscript Click here to access/download;Manuscript;+TabanidThermalTrapping_PLoS

Why Do Biting Horseflies Prefer Warmer Hosts? Tabanids can Escape Easier from Warmer Targets

Gábor Horváth[1]*, Ádám Pereszlényi[1,2], Ádám Egri[3,4], Tímea Tóth[1] and Imre M. Jánosi[5]

1: Department of Biological Physics, ELTE Eötvös Loránd University, H-1117 Budapest, Pázmány sétány 1, Hungary

2: Department of Zoology, Hungarian Natural History Museum, H-1083 Budapest, Ludovika tér 2-6., Hungary

3: MTA Centre for Ecological Research, Danube Research Institute, H-1113 Budapest, Karolina út 29-31, Hungary

4: MTA Centre for Ecological Research, Evolutionary Systems Research Group, Klebelsberg Kuno utca 3, H-8237 Tihany, Hungary

5: Department of Physics of Complex Systems, ELTE Eötvös Loránd University, H-1117 Budapest, Pázmány sétány 1, Hungary

*: correspondig author, e-mail: gh@arago.elte.hu

Abstract

Blood-sucking horseflies (tabanids) prefer warmer (sunlit, darker) host animals and generally attack them in sunshine, the reason for which was unknown until now. Recently, it was hypothesized that blood-seeking female tabanids prefer elevated temperatures, because their wing muscles are quicker and their nervous system functions better at a warmer body temperature brought about by warmer microclimate, and thus they can more successfully avoid the host's parasite-repelling reactions by prompt takeoffs. To test this hypothesis, we studied in field experiments the success rate of escape reactions of tabanids landed on black targets as a function of the target temperature, and measured the surface temperature of differently coloured horses with thermography. We found that the escape success of tabanids decreased with decreasing target temperature, that is escape success is driven by temperature. Our results explain the behaviour of biting horseflies that they prefer warmer hosts against colder ones. Since in sunshine the darker the host the warmer its body surface, our results also explain why horseflies prefer sunlit dark (brown, black) hosts against bright (beige, white) ones, and why these parasites attack their hosts usually in sunshine, rather than under ~~shady~~ conditions.

Media Abstract

We proposed that horseflies prefer sunlit darker host animals, because they enjoy increased escape success on darker animals. We suggest that this is due to sunlit darker surfaces being warmer than brighter one, which in turn lets horseflies escape faster because of their increased muscle and nervous system performance. To test this hypothesis, we first measured the thermal properties of the surface of horses. This was then compared against the escape response of horseflies that had landed on barrels of different temperatures (in shade/sun, and/or containing warm air or cold water). We found that the escape response of horseflies increased with increasing barrel temperature, that is escape success is driven by temperature. Our finding explains why horseflies prefer sunlit darker hosts.

Keywords: Tabanidae; horsefly; thermoreception; warmth preference; host animal; parasite-host interaction

Introduction

Blood-sucking horseflies (tabanids) prefer warmer (sunlit, darker) host animals against colder (~~shady,~~ brighter) ones and generally attack them in sunshine [1, 2, 3, 4, 5]. Tabanids attack black cattle more frequently than white ones [6]. Among white, brown and black cattle, black individuals are the preferred targets of *Tabanus* spp. horsefly attacks [7]. The attractiveness of sunlit brown horses to tabanids is about four times larger than that of sunlit white ones, and in comparison with a white horse, a brown horse spends two times longer ~~period~~ in a tabanid-free ~~shady~~ forest than in a sunny field with intense tabanid attacks [1]. The most effective tabanid traps use shiny black decoys [8, 9, 10, 11, 12, 13, 14, 15]. The so-called H-traps – composed of a bright tent with a shiny black sphere suspended below it – placed in sunny sites capture significantly more female tabanids than at ~~shady~~ sites [16]. The reason for this is that sunlit shiny dark targets reflect light at the Brewster's angle with higher degrees of linear polarization *d* than ~~shady~~ ones [17, 18], and host-seeking female tabanids prefer high *d*-values independent of the direction of polarization [19]. Thus, shiny black decoys used to catch horseflies work ~~for~~ their colour and reflected degree of polarization, rather than ~~for~~ their temperature.

After these experimental and observational findings concerning tabanid thermal preference, Horváth *et al.* [20] showed that *Tabanus tergestinus* horseflies prefer sunlit warm shiny black targets ~~against~~ sunlit or ~~shady~~ cold ones with the same optical characteristics. Furthermore, they hypothesized that a blood-seeking female tabanid prefers elevated temperatures, because her wing muscles are quicker and her nervous system functions better in a warmer microclimate, and thus she can more successfully avoid the host's parasite-repelling reactions by prompt takeoffs. Of course, there could also be other reasons why blood-sucking horseflies might prefer to attack warmer host animals. For example, to increase sweating, the capillaries could be enlarged near the epidermis of warmer hosts, which could be advantageous for blood-sucking insects.

The prediction of the hypothesis of Horváth *et al.* [20] is that the escape success of horseflies landed on host animals increases with increasing surface temperature. To test this prediction, we studied ~~in field experiments~~ the escape success of tabanids landed on black targets as a function of the surface temperature, and measured the coat temperature of differently coloured sunlit and ~~shady~~ horses with thermography. The results of our field experiments presented here corroborated ~~the mentioned~~ prediction which explains why blood-seeking horseflies prefer sunlit dark (warmer) host animals.

Results

As expected, the surface temperature T of the sunlit back of horses decreased in the colour order black > brown > beige > white, and the mean temperature $<T>$ of the bellies had smaller standard deviation ΔT than the backs (Fig. 1, Supplementary Figs. S1-S4). The minimum and maximum surface temperatures of horses were: black: 30.9-54.6 °C, brown: 31.2-44.6 °C, beige: 32.6-46.2 °C, white: 31.0-46.6 °C. The range T_{max} - T_{min} and ΔT increased with increasing $<T>$ (Fig. 2, Supplementary Tables S1-S4).

Figure 3 displays the surface temperature range $T_{min} \leq T \leq T_{max}$ of barrels and the percentages of escape success and capture rate of tabanids landed on the barrels under different illumination and thermal conditions. Considering experiments 1-3, the escape success was the highest on the sunlit air-filled barrel (85.4 %, p < 0.001, χ^2 = 48.167, df = 1, significant), it was the lowest on the shady water-filled barrel (28 %, p = 0.02781, χ^2 = 4.84, df = 1, significant), and on the shady air-filled barrel it was in between the former two (54.5 %, p = 0.5465, χ^2 = 0.36364, df = 1, not significant). Under sunlit conditions in experiment 4, the escape success on the air-filled barrel (81.3 %) was definitely higher by a factor of 2.3 (p < 0.001, χ^2 = 34.9634, df = 1) than that on the water-filled barrel (35.6 %). In experiment 5, tabanids could escape also with a significantly higher success (p < 0.001, χ^2 = 32.5403, df = 1) from the sunlit side of the air-filled barrel (86.4 %) than from its shady side (39.5 %), similarly to the sunlit (45 %) and shady (29.4 %) sides of the water-filled barrel (p = 0.074398, χ^2 = 3.1832, df = 1, non-significant). The numbers of captured and escaped tabanids were not significantly different in the following situations: shady side of the air-filled barrel in experiment 2, shady side of the air-filled barrel in experiment 5, and sunlit side of the water-filled barrel in experiment 5 (Fig. 3, Supplementary Table S5). According to Fig. 3 (Supplementary Tables S6-S10), the surface of air-filled barrels was always warmer than that of water-filled ones, and the sunlit surface of a given barrel was warmer than its shady side. All these results support our hypothesis that tabanids can escape more successfully from warmer targets than from cooler ones.

According to Fig. 4A, the number N_e of escaped tabanids landed on barrels has a maximum at around 41 °C, drops to zero at 17 °C and decreases almost to zero at 62 °C. The explanation of the drop of N_e at lower surface temperatures is that (i) less tabanids landed on colder surfaces, because tabanids flying through the cold boundary layer around the cold barrels could sense the lower surface temperature by their thermoreceptors (in the legs, antennae, mouthpart) and decided less frequently to alight, as Horváth *et al.* [20] showed, and (ii) the escape rate is lower at colder surfaces (see Fig. 4C). At higher surface temperatures more tabanids landed on the barrels, but because of the hot surface many of them flew away before the capturing attempt was performed in 10 seconds after landing (see Materials and Methods and Discussion). Therefore, the landing events shorter than 10 seconds were not registered, thus N_e droped with increasing T. In Fig. 4B, the number N_c of captured tabanids landed on barrels exhibits a clear decreasing trend with increasing T_{barrel} for both temperature intervals of 17 °C $\leq T \leq$ 62 °C and T_{min} = 31 °C $\leq T \leq T_{max,BL}$ = 55 °C. In Fig. 4C the increasing tendency of the normalized escape succes $e = N_e/(N_e + N_c)$ with increasing barrel's surface temperature T_{barrel} is clear. Figure 4C also illustrates that the systematic increase of the escape success e is also present in the temperature range T_{min} = 31 °C $\leq T \leq T_{max,BL}$ = 55 °C that is typical for the surface of horses.

We can see in Fig. 5A that the barrel's surface temperature T_{barrel} and the air temperature T_{air} correlate positively. According to Fig. 5B, there is a positive correlation between the normalized escape success $e = N_e/(N_e + N_c)$ and T_{air} if we take into consideration the results of all five experiments. Since T_{air} and T_{barrel} correlate positively (Fig. 5A) and e increases with increasing T_{barrel} (Fig. 4C), it could also be expected that e also increases with increasing T_{air} as seen in Fig. 5B. However, applying linear regression only for e-values measured at air temperatures lower than 33 °C (this way the warmest observations are eliminated when only sunlit air-filled warm barrels were used in experiment 1 resulting in a strong bias in the escape success e), the regressed line becomes horizontal as Fig. 5C displays. In this case there is no correlation between e and T_{air}. This suggests

that the ~~landed~~ horseflies spent ~~enough~~ time (10 seconds) on the barrel so that T_{barrel} determined the escape success, rather than T_{air}.

~~According to~~ Fig. 5D~~,~~ the normalized escape success e positively correlates with the temperature difference $\Delta T = T_{barrel} - T_{air}$ (°C) for all five experiments. Note that the 17-62 °C range of T_{barrel} is larger than the 23-38 °C range of T_{air}. This means that if ΔT is low/high, then T_{barrel} is also low/high. Thus, the result in Fig. 5D is similar to that in Fig. 4C, because only the temperature range (horizontal axis) was changed, which resulted in some blur due to the relatively small variation T_{air}.

~~In~~ Fig. 6A the results of a logistic regression show a highly significant ($p < 0.0001$) positive correlation between ~~the barrel's surface temperature~~ T_{barrel} and the escape probability ε of ~~landed~~ tabanids (Supplementary Table S11). The effect of T_{barrel} in the logistic regression ~~is~~ also significant ($p = 0.000395$) for the temperature range 31-55 °C (Supplementary Table S12). The logistic regression in Fig. 6B displays a positive correlation between the air temperature T_{air} and the escape probability ε of tabanids, and the effect of T_{air} ~~is~~ highly significant ($p < 0.0001$, Supplementary Table S13). Figure 6C ~~represents a~~ highly significant ($p < 0.0001$) positive correlation between the difference $T_{barrel} - T_{air}$ and the escape probability ε of tabanids (Supplementary Table S14). These findings correspond to the results of the linear regressions.

Discussion

Female tabanids prefer to attack sunlit against ~~shady~~ dark host animals, and dark against bright hosts for a blood meal, the exact reasons for which were unknown. Our results presented here show that the surface temperature of sunlit darker horses is higher than that of sunlit brighter horses. This result corresponds to previous measurements [21, 22, 23, 24, 25, 26, 27]. The differences in surface temperatures of dark and bright as well as sunlit and ~~shady~~ hosts may partly explain their different attractiveness to tabanids. Horváth *et al.* [20] found that *Tabanus tergestinus* horseflies prefer sunlit warm shiny black targets against sunlit or ~~shady~~ cold ones with the same optical characteristics. They hypothesized that blood-sucking female tabanids prefer higher temperature, because their wing muscles are quicker and their nervous system functions better in a warmer microclimate, therefore they can avoid the parasite-repelling reactions of host animals by prompt takeoffs. Since the thermoreceptors of tabanids (as ~~usually in Diptera~~) are in their legs, antennae and mouthpart [28, 29, 30], they cannot sense ~~remotely~~ (e.g. by infrareceptors) the temperature of a target. They can sense ~~precisely~~ the surface temperature of a substrate/host only after ~~a~~ physical contact (landing). However, ~~on the basis of~~ the leg/antenna/mouth-sensed temperature of the boundary layer around a target, tabanids can decide whether the target's surface is or is not warm enough for alighting [20].

The blood meal from warm-blooded animals is used by biting female horseflies as an energy source for maturation of their eggs [25, 28, 29, 30]. For this purpose, the blood of any warm-blooded hosts is ~~good,~~ regardless ~~if hosts are~~ dark- or bright-coloured, ~~or shady~~ or sunlit. In spite of this, blood-seeking female tabanids prefer dark and sunlit hosts [1, 2, 3, 4, 5, 6, 7], and this is the reason why horsefly traps usually have black decoys and are most effective in sunshine [8, 9, 10, 11, 12, 13, 14, 15, 16]. Our main assumption was that blood-seeking tabanids prefer to land on sunlit dark hosts to keep their body warm, which aids their rapid escape when the host performs such typical antiparasite reactions as removing horseflies from their coats with tails brushing ~~them off~~, ~~by~~ stamping and dislodging the flies, or by nibbling their ~~skin~~, ~~for instance~~ [1, 30]. These fly-repelling reactions are dangerous for blood-sucking tabanids, therefore have to be avoided by a quick flying away.

In this work we analysed the results of our field experiments for the whole tabanid population of the study area without considering differences between tabanid species/genus, because species/genus identification was not feasible in the field. However, there may be differences between species/genus in landing, daily activity and responses to environmental parameters that might influence their escape success. For example, Haematopota species might be more active in

late afternoon when ~~air temperature~~ decreases. It is also unknown whether the influence of ~~air temperature~~ T_{air} on host preference for *Haematopota* sp. is lower than that for *Tabanus* or *Atylotus* sp. To test these hypotheses could be the ~~task~~ of further studies. What we know from our field~~s~~ experiments is the following: (i) ~~Apart~~ from T_{air} (Supplementary Tables S6-S10) the weather situation (calm with no meteorological fronts) was the same during our field experiments. (ii) There was no correlation between the escape success *e* of ~~landed~~ horseflies and T_{air} < 33 °C (Fig. 5C). In this case ~~the barrel temperature~~ T_{barrel} determined the escape success *e*, rather than T_{air}.

According to earlier field experiments [31, 32] in the same experimental site (a Hungarian horse farm in Szokolya) with the same tabanid species (*Tabanus tergestinus*, *T. bromius*, *T. bovinus*, *T. autumnalis*, *Atylotus fulvus*, *A. loewianus*, *A. rusticus*, *Haematopota italica*) as in the ~~case of our~~ present field experiments, the daily activity of different tabanid species and the effect of weather variables on their flight activity ~~are~~ slightly different. Herczeg *et al*. [32], for example, found the following: (i) ~~Rainfall,~~ air temperature, and sunshine were the three most important factors influencing the ~~trapping~~ number of tabanids. (ii) The effect of relative [illegible] humidity *H* on tabanids was indirect through the ~~air temperature~~ *T*: *H* ≈ 35 % (corresponding to *T* ≈ 32 °C) was optimal for tabanid ~~trapping,~~ and tabanids were not captured ~~for~~ *H* ≥ 80 % (corresponding to *T* ≤ 18 °C). (iii) A fast decrease in the air pressure enhanced the ~~trapping number of~~ horseflies. (iv) Wind velocities ~~larger than~~ 10 km/h ~~reduced drastically~~ the number of trapped tabanids.

In our field experiments 4 and 5 warm and cold sunlit barrels were used simultaneously, while in experiments 1-3 the cold and the warm barrels were tested separately (experiment 1: sunlit air-filled barrels, experiment 2: ~~shady~~ air-filled barrels, experiment 3: ~~shady~~ water-filled barrels). This was, however, not a problem, because apart from the air temperature (Supplementary Tables S6-S10) the environmental conditions (calm with no meteorological fronts) were practically ~~the same~~ on all experimental days. Thus, the slightly different environmental factors in our field experiments could have resulted in only small differences in the activity of a given tabanid species.

In our field experiments the investigator waited 10 seconds before he tried to capture a tabanid landed on a barrel. This 10-second period turned out to be optimal: it was neither too short, nor too long. Within a period shorter than 10 s the flight muscles of ~~landed~~ tabanids could not warm up or cool down to the surface temperature of barrels. On the other hand, when a ~~landed~~ tabanid recognizes that the barrel is not a host animal, it flies away after a certain period. If the waiting period ~~of~~ counting of landed tabanids ~~were~~ too long, then many landing events would not be counted/registered. ~~The air temperature~~ did not correlate with the escape rate (Fig. 5C), ~~therefore~~ 10 s was long ~~enough~~ to warm the wing muscles of flies landed on the barrel. On the other hand, if a tabanid lands on a sunlit dark (warm) surface, then it ~~does~~ not cool down, contrary to a bright (colder) surface where its wing muscles can cool down. It is ~~pertinent~~ to suppose that during flight the wing muscles are appropriately warm. Furthermore, according to Heinrich [33], many Dipteran species (including true flies and also tsetse flies) can be heterothermic, or generate certain amount of heat which can then be used to improve their performance. However, as far as we know, practically nothing is known about the thermoregulation in horseflies. Thus, without knowing the horsefly thermal physiology, we restricted our study to the correlation between (air/barrel) temperature and escape success with a waiting period of 10 seconds before the experimenter tried to capture a tabanid landed on a barrel.

During the field experiments all barrels were optically uniformly attractive to host-seeking horseflies. Our air-filled sunlit warm barrels (37-62 °C) thermally imitated sunlit black horses (31-55 °C), while our water-filled sunlit cold barrels (19-29 °C) were cooler than sunlit brown, beige and white horses (31-47 °C). Although the measured low escape successes in the case of our water-filled cold barrels were associated with temperatures that were lower than those on the studied horse bodies, the positive correlation between escape success *e* and surface temperature *T* (i.e. increasing *e* with increasing *T*) is also evident in the interval T_{min} = 31 °C ≤ *T* ≤ $T_{max,BL}$ = 55 °C as clearly shown by the dotted curves of Figs. 4C and 6A. Thus, the results of our field experiments show that the escape success of tabanids depends on the host's surface temperature *T*: the higher the *T*, the

larger the escape success of horseflies. The average surface temperature of the studied sunlit black horses was between 48 and 55 °C. Almost all tabanids managed to escape in this temperature range.

In Figs. 4 and 6A, the 100 % escape successes at surface temperatures $T > 50$ °C are associated with very small absolute numbers of horseflies and also with hot air temperatures ranging from 33 to 38 °C (Supplementary Table S6). The reason for this is that only the minority of tabanids landing on hot ($T > 55$ °C) surfaces spent periods longer than 2 seconds on them, and the waiting time until a catch attempt was 10 seconds after a tabanid landed on a barrel. In experiment 1 the weather was very warm ($T_{air} \leq 38$ °C) and the surface temperature of the warm barrel was above 45 °C the whole day (Supplementary Table S6). Larger horseflies can tolerate extreme temperatures for a short time, and are also intrinsically faster [30].

The thermal transfer between a horsefly and a surface could be very different in the following two cases: (i) When a fly lands on a horse, the grip is maintained by the insect's legs holding on the hairs, leaving plenty of insulating air between the two animals. (ii) When a fly lands on a plastic barrel, its legs must remain attached to a much less convenient substrate and the thermal transfer could be different (perhaps more efficient on plastic). In the future, an important task could be to measure both thermal transfers.

It has been shown that dark-bodied host animals have a much stronger reflection-polarization signature than bright-bodied ones, which is an important visual sign, leading to horsefly attacks [18]. The high degrees of polarization of reflected light helps tabanids to select sunlit dark host animals from the dark patches of their visual environment. A strong polarization signature could also advertise a hot animal and greater chances of escape of tabanids, but both traits could be also dissociated, all depending on the thermal transfer and the thermal physiology of horseflies.

Conclusion

We presented here the results of our field experiment studying the dependence of tabanid escape success on the temperature of the landing surface. The temperature of artificial landing sites was parallelled by the analysis of thermal imaging measurements of surface temperatures in different body parts of differently coloured horses. Not surprisingly, fur temperatures were higher in darker horses and lower in bright coloured ones. The tabanid escape success strongly depended on the surface temperature; the highest escape success occurred on surfaces having temperatures similar to those recorded in black horses, i.e. above 50 °C. We drew the conclusion that the warmer (also darker) host animals allow higher escape success of blood-sucking horseflies. This supports our hypothesis that the preference of horseflies to dark hosts has partly evolved due to higher survival rate.

Materials and Methods

Animal Ethics Statement and Field Study Permits

Csaba Viski permitted us to photograph his horses in his horse farm in Szokolya. For the location and activities of our field study no specific permissions were required.

Thermography of horses

On a warm day (6 July 2019) 46 thermograms of 2 black, 2 brown, 2 beige and 2 white horses were measured with an infrared camera (VarioCAM®, Jenoptik Laser Optik Systeme GmbH, Jena, Germany, nominal precision of ±1.5 °C) under sunny and cloudy conditions. The validation and calibration of this thermocamera with a contact thermometer (GAO Digital Multitester EM392B 06554H, EverFlourish Europe Gmbh., Friedrichsthal, Germany, nominal precision of ±1 °C) were described in the Supporting Material of [20].

Field experiments

Field experiments 1-5 were performed on 1, 2, 3, 4 and 11 July 2019 on a Hungarian horse farm in Szokolya (47° 52' North, 19° 00' East), where horseflies were present. All five experimental days were windless, and only weak local winds blew in early afternoons. Meteorological fronts did not move through the site ~~of experiments~~. Thus, apart from the air temperature (according to Supplementary Tables S6-S10, in experiment 1 the air temperatures were warmer than on the other four experimental days), the environmental conditions were practically the same. In mornings, the weather was sunny, warm and cloudless, however in the afternoons a few cumulus clouds formed. In these experiments the escape success of tabanids landed on shiny black cylindrical plastic barrels (height = 42 cm, diameter = 30 cm, wall thickness = 5 mm) of different surface temperatures (set with warm air or cold water load) but with the same optical characteristics was studied under sunlit and ~~shady~~ conditions. The purpose of these barrels was to imitate warm and cold dark host animals of tabanids. ~~The high enough~~ temperature differences between the warm and cold barrels could easily be ensured with air-filled warm and water-filled cold barrels. An experimenter, who was "blind" to the predictions of the experiment tried to capture the tabanids landed on the barrels with a hemispherical tea-strainer of diameter 15 cm. The ~~waiting time until a catch~~ attempt was 10 seconds (measured with a stopwatch) after a tabanid landed on a barrel (the choice of this optimal 10-second value is explained in the Discussion).

- In **experiment 1** (1 July 2019, 10:20-17:00 hour = local summer time = UTC + 2 h) two air-filled sunlit black barrels were used, which thermally modelled sunlit black host animals (e.g. horses) for tabanids. The two barrels were put on top of each other, and both were placed on a four-legged white plastic stand (height = 46 cm) at a sunlit site without any shade cast by vegetation or other objects. Only tabanids landed on the sunlit side of the barrels were taken into account.
- In **experiment 2** (2 July 2019, 9:40-16:00 h) two air-filled barrels ~~under shadow~~ were used, which modelled ~~shady~~ hosts. The barrels were put on top of each other and the white stand was in the shade of trees during the experiment. Only tabanids landed on the side of the barrels facing toward the open field were considered.
- In **experiment 3** (3 July 2019, 9:50-16:00 h) two cold-water-filled ~~shady~~ barrels were used, which modelled cool ~~shady~~ hosts. The barrels were continuously ~~under~~ the shadow of trees. Both barrels were filled with tap water and 10 frozen ice packs (Aspico G40, 0.25 litre, 0.76 kg). The experimenter tried to capture only tabanids landed on the side of the barrels facing toward the open field.
- In **experiment 4** (4 July 2019, 10:00-12:00 h) two sunlit air-filled barrels and two sunlit cold-water-filled barrels were used which thermally modelled sunlit and ~~shady~~ hosts, respectively. Both barrels were continuously exposed to sunlight. Only tabanids landed on the sunlit side of the barrels were tried to capture.
- **Experiment 5** (11 July 2019, 10:20-16:00 h) was technically the same as experiment 4, but all tabanids ~~landed~~ on both sunlit and ~~shady~~ sides of barrels were subject of attack.

The experimenter wore white clothes and hat against direct sunshine and to minimize his visual attractiveness to tabanids. He was sitting on a chair during the experiments next to the barrels (50 cm) in such a way that he could easily reach the tabanids on the barrels with the tea-strainer. After the fly was successfully caught, it was released. After each capture trial the air temperature and the surface temperature of the barrel at the tabanid's landing location was measured ~~by~~ a contact thermometer (GAO Digital Multitester EM392B 06554H, EverFlourish Europe Gmbh., Friedrichsthal, Germany, nominal precision of ±1 °C). For this purpose, thermography was not possible to use because of technical problems. The experimenter was the same person throughout all experiments, who ~~has practiced himself in tabanid capture~~ during a pilot experiment. In the

vicinity of the barrels there were no many flying tabanids. Threfore, only one tabanid landed on one of the two (warm or cold) barrels at a given time, thus the experimenter did not have to split his attention between two or more landed tabanids.

An *in situ* identification of the species of tabanids landed on the barrels was not feasible. It was obvious, however, that they were tabanids (Diptera: Tabanidae). In previous field experiments [31, 32], the following tabanid species occurred at the same study site: *Tabanus tergestinus*, *T. bromius*, *T. bovinus*, *T. autumnalis*, *Atylotus fulvus*, *A. loewianus*, *A. rusticus*, *Haematopota italica*. Since we could record the escape success of different tabanid species, our results can be considered as the average escape success of the tabanid population of the experimental site.

Since the reflection-polarization characteristics of the dry barrel's surface are independent of its temperature in the visible spectral range, all optical parameters (radiance, degree of linear polarization and angle of polarization) of our warm and cool barrels ~~were the same~~.

Statistical analysis

For comparison of the numbers of escaped and captured tabanids landed on test surfaces of various temperatures, we applied χ^2 tests of homogeneity, where the escape versus non-escape ratio was tested against the predicted 50/50 ratio. These χ^2 tests were performed to compare escape/non-escape numbers for a given barrel or barrel side (sunlit or ~~shady~~). Thus, the compared escape/non-escape numbers corresponded to the same barrel temperature and there was no comparison between data originating from different barrel temperatures. In other words, χ^2 test was used to detect whether a given barrel temperature had an effect on tabanid escape success.

Linear regressions were applied to find a trend of the escape success of horseflies as a function of the surface temperature T_{barrel}, air temperature T_{air} and the temperature difference $T_{barrel} - T_{air}$ between the barrel surface and the air. We also applied logistic regression to model the probability of escape as a function of T_{barrel}, T_{air} and $T_{barrel} - T_{air}$. We also applied the linear and logistic regressions as a funciton of T_{barrel} using the data within the 31 °C $\leq T \leq$ 55 °C interval. Logistic regression was also used to find whether there is a correlation between T_{air} and T_{barrel}. The R statistical package 3.0.2 [34] was used for statistical analyses.

Copyrighted Figures

There is no any element of our submission (e.g. figures, supporting information, striking image) under copyright. In our manuscript nothing has been adapted from copyrighted material.

Supporting Information

Our paper has the following electronic supporting materials: Supplementary Tables S1-S11, Supplementary Figures S1-S4.
(DOC)

Supplementary Table S1: Temperatures of black horses measured with thermography on ~~shady~~ and sunlit sides of the back and belly, and when the sun was occluded by clouds (cloudy). $\langle T \rangle$: average, $\pm\Delta T$: standard deviation, T_{min}: minimum, T_{max}: maximum.
(DOC)

Supplementary Table S2: Temperatures of brown horses measured with thermography on ~~shady~~ and sunlit sides of the back and belly, and when the sun was occluded by clouds (cloudy). $\langle T \rangle$: average, $\pm\Delta T$: standard deviation, T_{min}: minimum, T_{max}: maximum.
(DOC)

Supplementary Table S3: Temperatures of beige horses measured with thermography on ~~shady~~ and sunlit sides of the back and belly, and when the sun was occluded by clouds (cloudy). $<T>$: average, $\pm\Delta T$: standard deviation, T_{min}: minimum, T_{max}: maximum.
(DOC)

Supplementary Table S4: Temperatures of white horses measured with thermography on ~~shady~~ and sunlit sides of the back and belly, and when the sun was occluded by clouds (cloudy). $<T>$: average, $\pm\Delta T$: standard deviation, T_{min}: minimum, T_{max}: maximum.
(DOC)

Supplementary Table S5: Results of χ^2 tests comparing the sums of Supplementary Tables S6-S10 obtained in experiments 1-5.
(DOC)

Supplementary Table S6: Capture success (-: not captured, +: captured) of horseflies, and temperatures of the air (T_{air}) and the surface of the air-filled sunlit barrel (T_{barrel}) in experiment 1 on 1 July 2019.
(DOC)

Supplementary Table S7: Capture success (-: not captured, +: captured)) of horseflies, and temperatures of the air (T_{air}) and the surface of the air-filled ~~shady~~ barrel (T_{barrel}) in experiment 2 on 2 July 2019.
(DOC)

Supplementary Table S8: Capture success (-: not captured, +: captured) of horseflies, and temperatures of the air (T_{air}) and the surface of the cold-water-filled ~~shady~~ barrel (T_{barrel}) in experiment 3 on 3 July 2019.
(DOC)

Supplementary Table S9: Capture success (-: not captured, +: captured) of horseflies, and temperatures of the air (T_{air}) and the sunlit side of the surface of the air-filled warm barrel (T_{warm}) and the water-filled cold barrel (T_{cold}) in experiment 4 on 4 July 2019.
(DOC)

Supplementary Table S10: Capture success (-: not captured, +: captured) of horseflies, and temperatures of the air (T_{air}) and the surface of the air-filled sunlit barrel (T_{warm}) and the cold-water-filled sunlit barrel (T_{cold}) in experiment 5 on 11 July 2019.
(DOC)

Supplementary Table S11. Summary of the logistic regression. The escape probability ε of tabanids depends highly significantly on the barrel's surface temperature T_{barrel} in the interval 17 °C $\leq T_{barrel} \leq$ 62 °C. The large difference between the null deviance and the residual deviance suggests that the logistic regression model is accurate.
(DOC)

Supplementary Table S12. Summary of the logistic regression. The escape probability ε of tabanids depends highly significantly on the barrel's surface temperature T_{barrel} in the interval 31 °C $\leq T_{barrel} \leq$ 55 °C.
(DOC)

Supplementary Table S13. Summary of the logistic regression. The escape probability ε of tabanids depends highly significantly on the air temperature T_{air}.

(DOC)

Supplementary Table S14. Summary of the logistic regression. The escape probability ε of tabanids depends highly significantly on the temperature difference T_{barrel} - T_{air}.
(DOC)

Supplementary Figure S1. Photographs, thermograms and thermograms with selected back and belly areas of black horses under different illumination conditions. ~~shady: shady~~ side of the sunlit horse. sunlit: sunlit side of the sunlit horse. cloudy: illuminated by skylight when the sun was occluded by clouds.
(TIF)

Supplementary Figure S2. Photographs, thermograms and thermograms with selected back and belly areas of brown horses under different illumination conditions. ~~shady: shady~~ side of the sunlit horse. sunlit: sunlit side of the sunlit horse. cloudy: illuminated by skylight when the sun was occluded by clouds.
(TIF)

Supplementary Figure S3. Photographs, thermograms and thermograms with selected back and belly areas of a beige horse under different illumination conditions. ~~shady: shady~~ side of the sunlit horse. sunlit: sunlit side of the sunlit horse. cloudy: illuminated by skylight when the sun was occluded by clouds.
(TIF)

Supplementary Figure S4. Photographs, thermograms and thermograms with selected back and belly areas of white horses under different illumination conditions. ~~shady: shady~~ side of the sunlit horse. sunlit: sunlit side of the sunlit horse. cloudy: illuminated by skylight when the sun was occluded by clouds.
(TIF)

Author contributions

Substantial contributions to conception and design: GH, ÁE, ÁP
Performing experiments and data acquisition: GH, ÁP, TT, IMJ
Data analysis and interpretation: GH, ÁP, TT, IMJ
Drafting the article or revising it critically for important intellectual content: GH, ÁE, ÁP, IMJ

Competing interests

The authors have no competing interests.

Funding

This work was supported by the grant NKFIH K-123930 (Experimental Study of the Functions of Zebra Stripes: A New Thermophysiological Explanation) received by Gábor Horváth from the Hungarian National Research, Development and Innovation Office. Ádám Egri was supported by the Economic Development and Innovation Operational Programme (GINOP-2.3.2-15-2016-00057), the grant NKFIH PD-131738 and the János Bolyai Research Scholarship of the Hungarian Academy of Sciences. The funders had no role in study design, data collection and analysis, decision to publish, or preparation of the manuscript.

Acknowledgements

We are grateful to Csaba Viski who permitted ~~the performance of~~ our field experiments ~~in~~ his horse farm in Szokolya. We thank for the valuable and constructive comments of three anonymous reviewers.

References

1. Horváth G, Blahó M, Kriska G, Hegedüs R, Gerics B, Farkas R, Åkesson S. An unexpected advantage of whiteness in horses: the most horsefly proof horse has a depolarizing white coat. Proceedings of the Royal Society B 2010; 277: 1643-1650.

2. Blahó M, Egri Á, Báhidszki L, Kriska G, Hegedüs R, Åkesson S, Horváth G. Spottier targets are less attractive to tabanid flies: on the tabanid-repellency of spotty fur patterns. ~~Public Library of Science One~~ 2012; 7: e41138 (doi: 10.1371/journal.pone.0041138).

3. Blahó M, Egri Á, Száz D, Kriska G, Åkesson S, Horváth G. Stripes disrupt odour attractiveness to biting horseflies: Battle between ammonia, CO_2, and colour pattern for dominance in the sensory systems of host-seeking tabanids. Physiology and Behavior 2013; 119: 168-174.

4. Egri Á, Blahó M, Kriska G, Farkas R, Gyurkovszky M, Åkesson S, Horváth G. Polarotactic tabanids find striped patterns with brightness and/or polarization modulation least attractive: an advantage of zebra stripes. Journal of Experimental Biology 2012; 215: 736-745.

5. Krcmar S, Radolic V, Lajos P, Lukacevic I. Efficiency of colored modified box traps for sampling tabanids. Parasite 2014; 21: 67 (doi: 10.1051/parasite/2014068).

6. Tashiro H, Schwardt HH. Biological studies of horse flies in New York. Journal of Economical Entomology 1953; 46: 813-822.

7. Lendzele SS, Eisenbarth A, Christophe ZKR, Mavoungou JF, Renz A. Aspects of the bionomics of hematophagous symbovine dipterans in a hyper-infested rangeland of Ngaoundere (Adamawa-Cameroon). Journal of Asia-Pacific Entomology 2019; 22: 1019-1030.

8. Thorsteinson AJ, Bracken GK, Tostawaryk W. The orientation behaviour of horse flies and deer flies (Tabanidae: Diptera). V. The influence of the number of reflecting surfaces on attractiveness to tabanids of glossy black polyhedra. Canadian Journal of Zoology 1966; 44: 275-279.

9. Catts EP. A canopy trap for collecting Tabanidae. Mosquito News 1970; 30: 472-474.

10. Roberts RH. Attractancy of two black decoys and CO_2 to tabanids (Diptera: Tabanidae). Mosquito News 1977; 37: 169-172.

11. Wall WJ, Doane OW. Large scale use of box traps to study and control saltmarsh greenhead flies (Diptera: Tabanidae) on Cape Cod, Massachusetts. Environmental Entomology 1980; 9: 371-375.

12. Blahó M, Egri Á, Barta A, Antoni G, Kriska G, Horváth G. How can horseflies be captured by solar panels? A new concept of tabanid traps using light polarization and electricity produced by photovoltaics. Veterinary Parasitology 2012; 189: 353-365.

13. Egri Á, Blahó M, Száz D, Barta A, Kriska G, Antoni G, Horváth G. A new tabanid trap applying a modified concept of the old flypaper: Linearly polarising sticky black surfaces as an effective tool to catch polarotactic horseflies. International Journal for Parasitology 2013; 43: 555-563.

14. Egri Á, Blahó M, Száz D, Kriska G, Majer J, Herczeg T, Gyurkovszky M, Farkas R, Horváth G. A horizontally polarizing liquid trap enhances the tabanid-capturing efficiency of the classic canopy trap. Bulletin of Entomological Research 2013; 103: 665-674.

15. Meglic A, Ilic M, Pirih P, Skorjanc A, Wehling MF, Kreft M, Belusic G. Horsefly object-directed polarotaxis is mediated by a stochastically distributed ommatidial subtype in the ventral retina. Proceedings of the National Academy of Sciences of the United States of America 2019; 116: 21843-21853.

16. Otártics MZ, Altbäcker V, Solymosi K, Mátics R, Romvári R, Farkas S. Efficacy of H-traps is affected by exposure to sunshine. Natura Croatica 2019; 28: 257-269.

17. Horváth G. (editor) 2014; Polarized Light and Polarization Vision in Animal Sciences. Springer: Heidelberg, Berlin, New York

18. Horváth G., Szörényi T., Pereszlényi Á., Gerics B., Hegedüs R., Barta A., Åkesson S. Why do horseflies need polarization vision for host detection? Polarization helps tabanid flies to select sunlit dark host animals from the dark patches of the visual environment. Royal Society Open Science 2017; 4: 170735 (doi: 10.1098/rsos.170735)

19. Egri Á, Blahó M, Sándor A, Kriska G, Gyurkovszky M, Farkas R, Horváth G. New kind of polarotaxis governed by degree of polarization: attraction of tabanid flies to differently polarizing host animals and water surfaces. Naturwissenschaften 2012; 99: 407-416.

20. Horváth G, Pereszlényi Á, Tóth T, Polgár S, Jánosi IM. Attractiveness of thermally different uniformly black targets to horseflies: *Tabanus tergestinus* prefers sunlit warm shiny dark targets. Royal Society Open Science 2019; 6: 191119 (doi: 10.1098/rsos.191119).

21. Cena K. Investigations of absorption of solar radiation by cattle and horses with various coat colours. Acta Agraria et Silvestria 1966; 6: 93-138.

22. Cena K, Clark JA. Thermographic measurements of the surface temperatures of animals. Journal of Mammalogy 1973; 54: 1003-1007.

23. Benesch AR, Hilsberg S. Infrarot-thermographische Untersuchungen der Oberflächentemperatur bei Zebras. Zoologischer Garten 2003; NF 2: 74-82.

24. Benesch AR, Hilsberg-Merz S. Oberflächentemperaturen bei Zebrastreifen. Natur und Museum 2006; 136: 49-56.

25. Caro T. Zebra Stripes. 2016; University of Chicago Press: Chicago.

26. Horváth G, Pereszlényi Á, Száz D, Barta A, Jánosi IM, Gerics B, Åkesson S. Experimental evidence that stripes do not cool zebras. Scientific Reports 2018; 8: 9351 (doi: 10.1038/s41598-018-27637-1).

27. Cobb A, Cobb S. Do zebra stripes influence thermoregulation? Journal of Natural History 2019; 53: 863-879.

28. McGavin GC. Essential entomology: an order-by-order introduction. 2001; Oxford University Press: Oxford.

29. Elzinga RJ. Fundamentals of Entomology. 2003; 6th ed. Upper Saddle River, NJ: Prentice Hall.

30. Lehane MJ. The biology of blood-sucking in insects. 2005; 2nd ed, Cambridge University Press: Cambridge, UK.

31. Herczeg T, Blahó B, Száz D, Kriska G, Gyurkovszky M, Farkas R, Horváth G. Seasonality and daily activity of male and female tabanid flies monitored in a Hungarian hill-country pasture by new polarization traps and traditional canopy traps. Parasitology Research 2014; 113: 4251-4260.

32. Herczeg T, Száz D, Blahó B, Barta A, Gyurkovszky M, Farkas R, Horváth G. The effect of weather variables on the flight activity of horseflies (Diptera: Tabanidae) in the continental climate of Hungary. Parasitology Research 2015; 114: 1087-1097.

33. Heinrich B. The Hot-Blooded Insects: Strategies and Mechanisms of Thermoregulation. 1993; Springer: Heidelberg, Berlin, New York

34. R Core Team. R: a language and environment for statistical computing. R Foundation for Statistical Computing, 2013 Vienna http://www.R-project.org/.

Figures with Captions

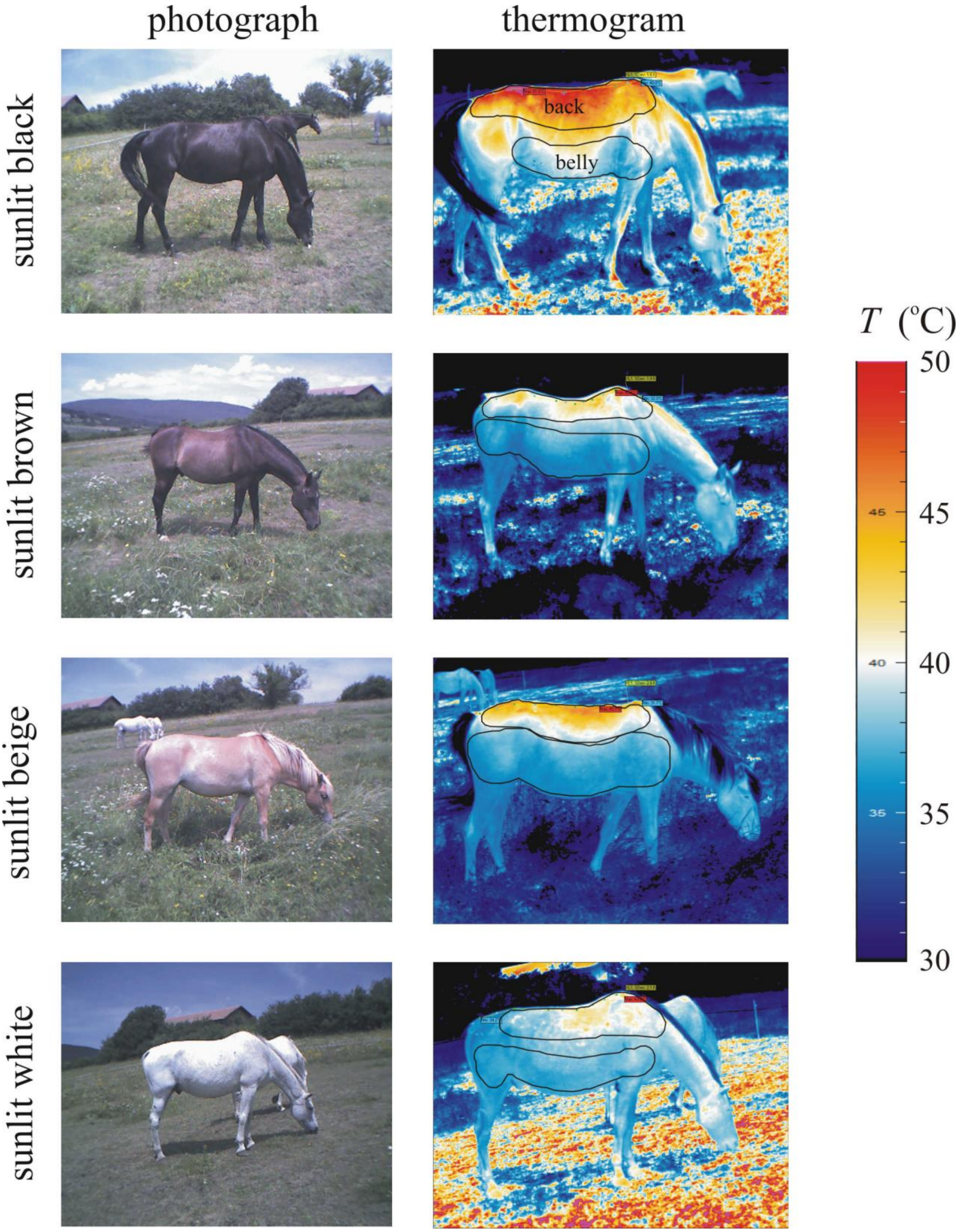

Figure 1. Thermograms of horses. Photographs and thermograms of sunlit black, brown, beige and white horses. In the thermograms the black perimeters of the back and belly areas are shown where the surface temperature T was averaged.

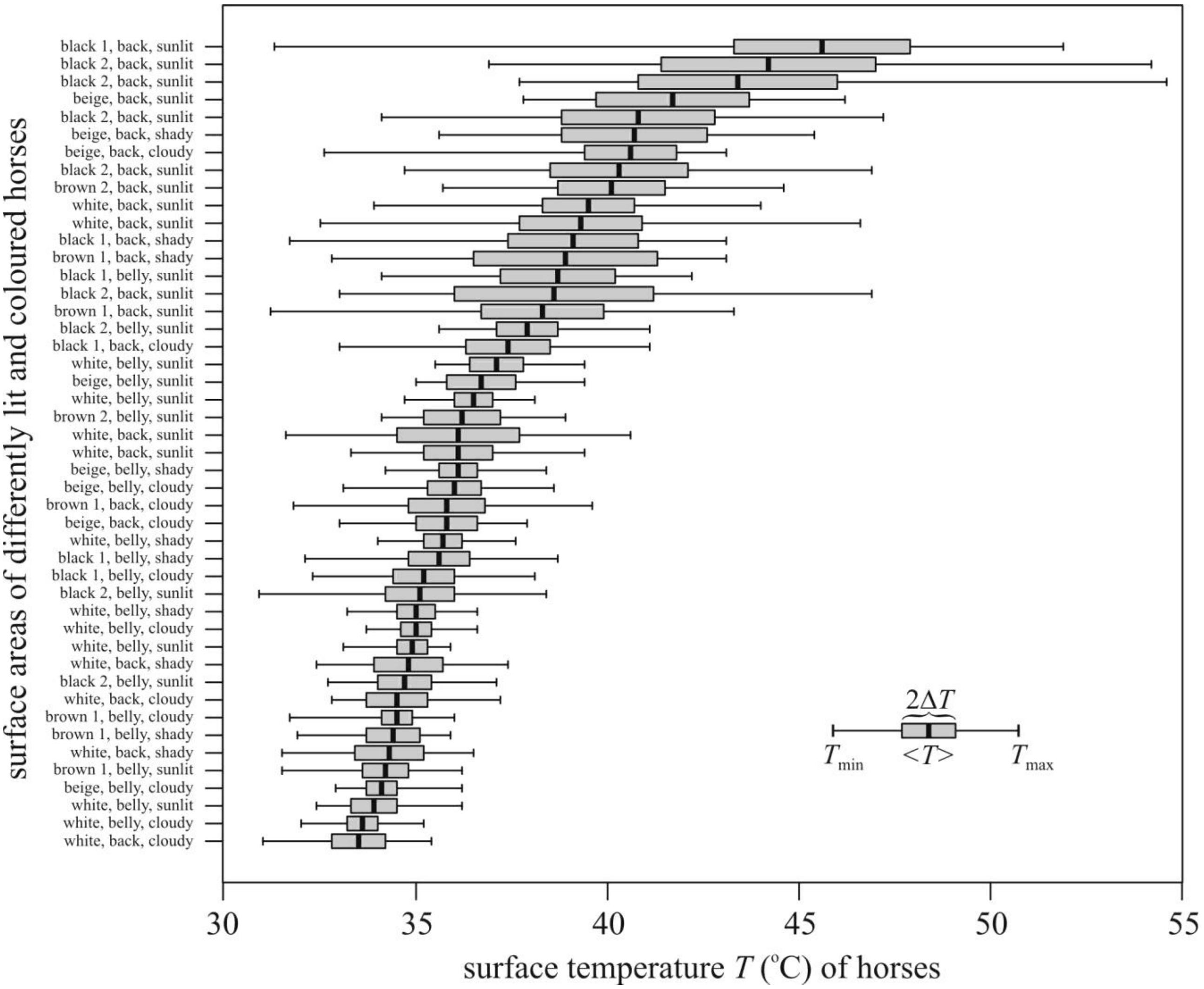

Figure 2. Surface temperatures of horses. Minimum (T_{min}), maximum (T_{max}), average (<*T*>) and standard deviation (ΔT) of the surface temperature of the back and belly of black, brown, beige and white horses measured with thermography under different illumination conditions (Supplementary Tables S1-S4, Supplementary Figs. S1-S4). shady: shady side of a sunlit horse, sunlit: sunlit side of a sunlit horse, cloudy: the horse was illuminated by skylight when the sun was occluded by clouds.

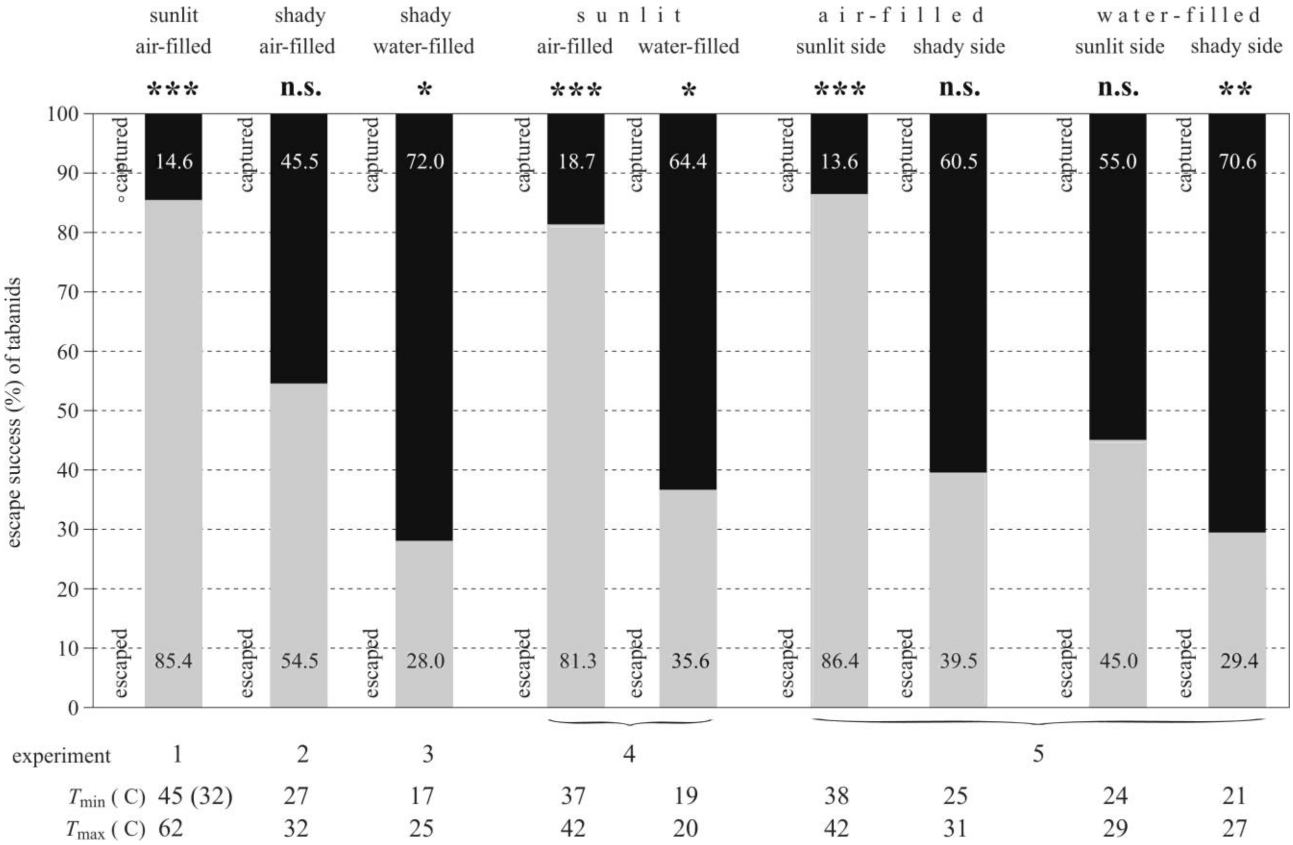

Figure 3. Escape success of horseflies in experiments 1-5. Surface temperature range $T_{min} \leq T \leq T_{max}$ of barrels and the percentage of escape success of tabanids (grey bars) landed on the barrels under different illumination and thermal conditions in experiments 1-5 (Supplementary Tables S6-S10). The results of χ^2 test are indicated: n.s: not significant, p > 0.05, *: 0.01 < p < 0.05, **: 0.001 < p < 0.01, ***: p < 0.001 (Supplementary Table S5). Grey and black bars illustrate the escape and capture rates, the percentage values of which are given in the columns.

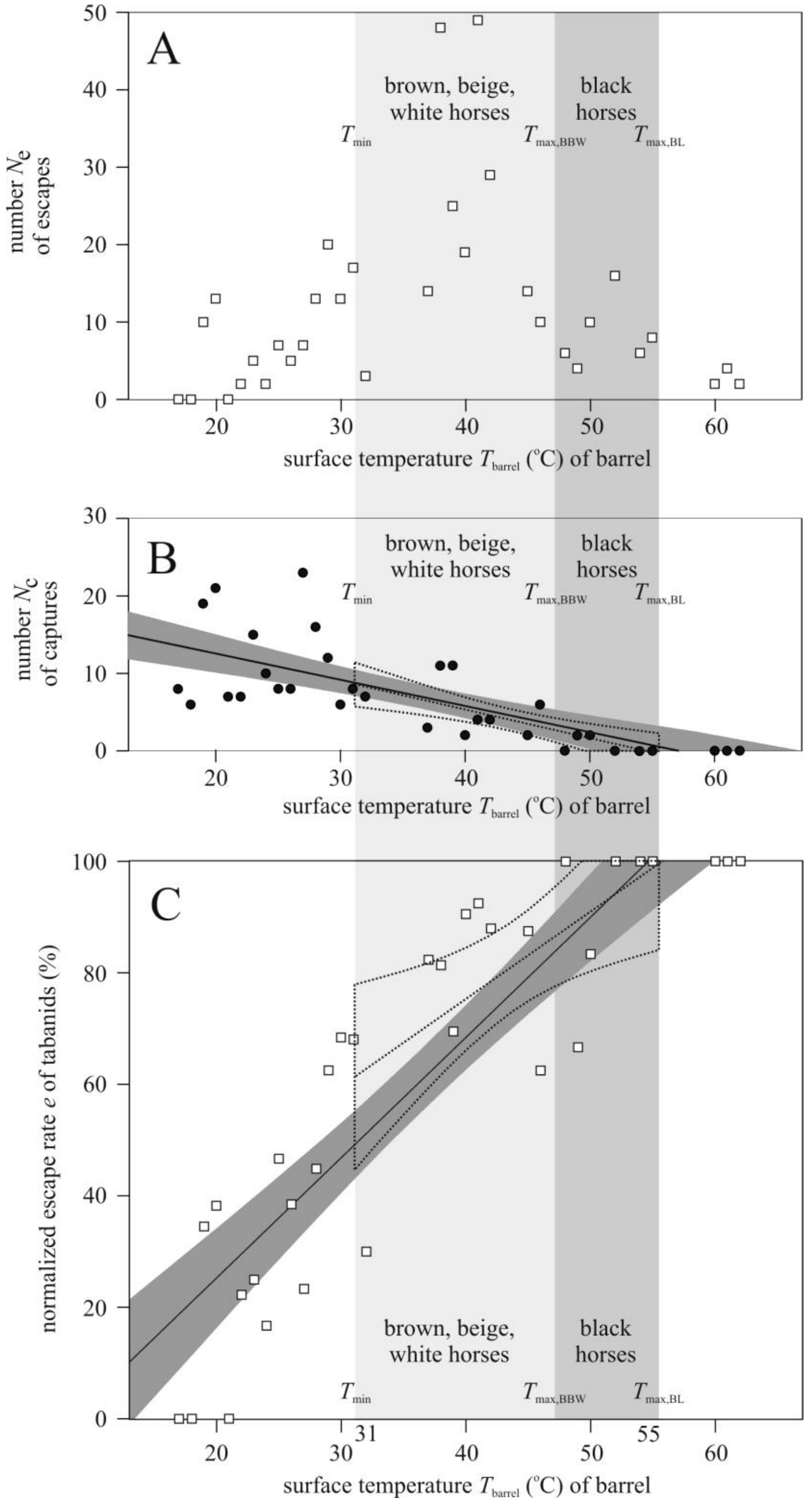

Figure 4. Escape success of horseflies versus surface temperature. Number N_e of escaped (A) and number N_c of captured (B) tabanids landed on barrels as a function of the surface temperature T_{barrel} (°C). In B a continuous straight line indicates the linear fit to all $N_c(T_{barrel})$ data (black circles in the interval 17 °C ≤ T ≤ 62 °C), and a dark grey band around this fit shows the 95 % confidence interval. (C) Normalized escape success $e = N_e/(N_e + N_c)$ versus T_{barrel} (°C). A continuous straight line and a dark grey band illustrate the linear fit to all $e(T_{barrel})$ data (white squares in the interval 17 °C ≤ T ≤ 62 °C) with 95 % confidence interval. The vertical light and medium grey columns denote the interval T_{min} = 31 °C ≤ T ≤ $T_{max,BBW}$ = 47 °C of the surface temperature of brown, beige and white horses (BBW) and the interval T_{min} = 31 °C ≤ T ≤ $T_{max,BL}$ = 55 °C of black (BL) horses measured by thermography (Fig. 2). In B and C a dotted straight line and a 95 % confidence interval with dotted perimeter illustrate the linear fit to the data within the 31 °C ≤ T ≤ 55 °C interval.

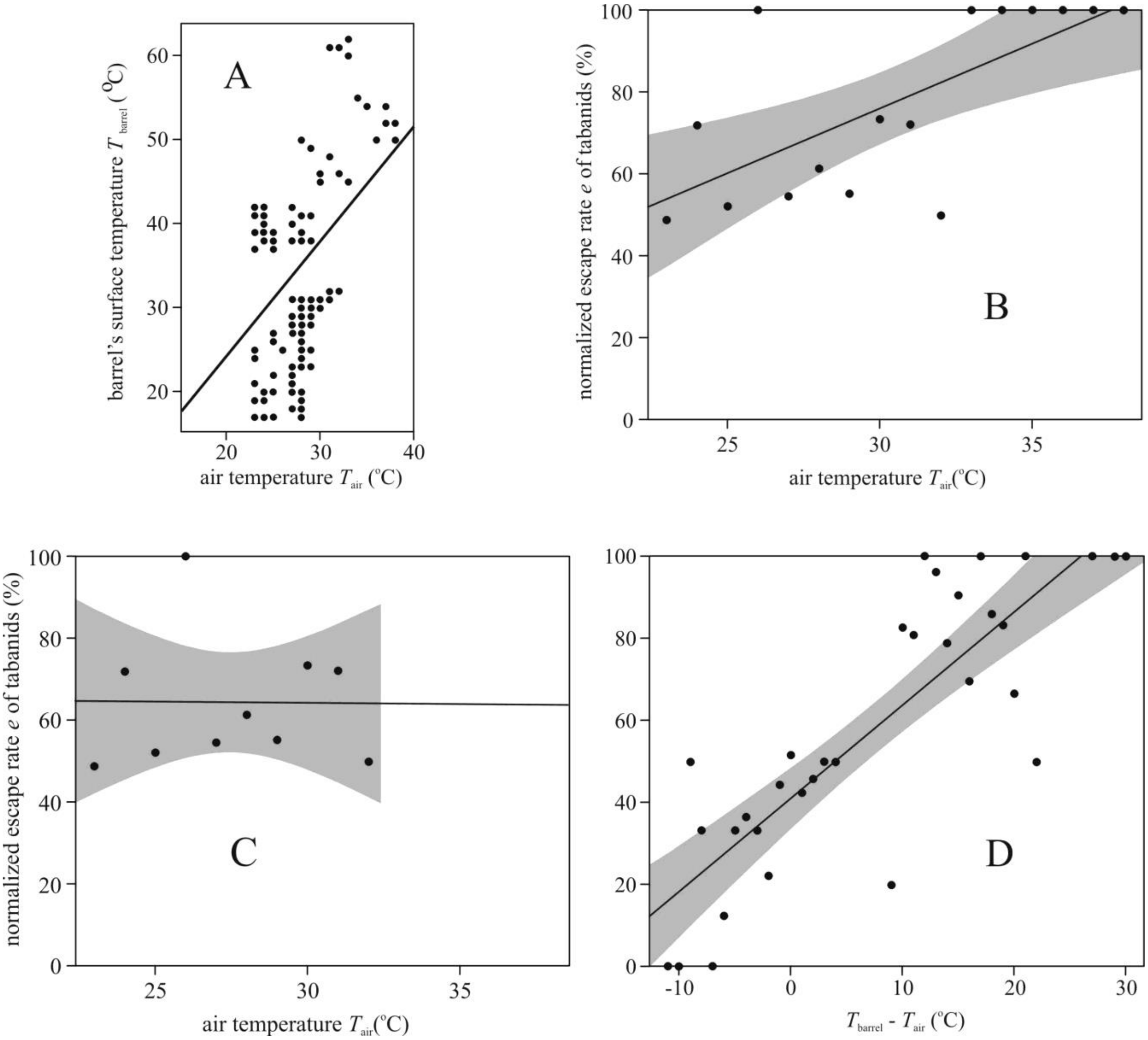

Figure 5. (A) The barrel's surface temperature T_{barrel} (°C) versus air temperature T_{air} (°C) in field experiments 1-5. A straight line indicates the linear fit to the $T_{barrel}(T_{air})$ data (black circles). (B) Normalized escape success *e* versus T_{air} (°C) for all five experiments. (C) As B, but only for $T_{air} \leq$ 32 °C. (D) Normalized escape success *e* versus T_{barrel} - T_{air} (°C) for all five experiments. In B-D continuous straight lines indicate the linear fit to the data (black circles), and dark grey bands around the fit show the 95 % confidence intervals.

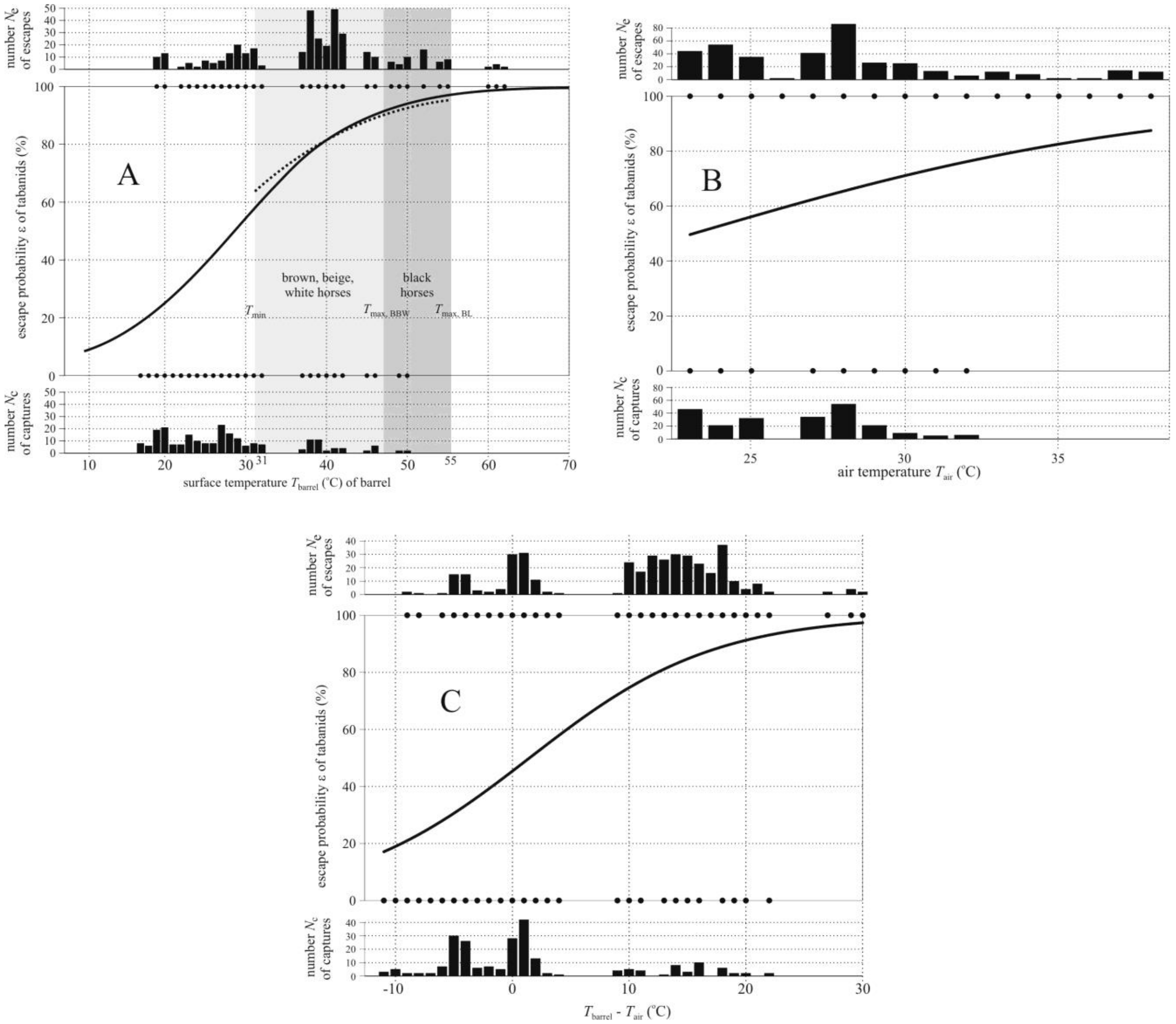

Figure 6. Results of logistic regressions. (A) *Top*: Number N_e of escaped horseflies versus the barrel's surface temperature T_{barrel}. *Middle*: Escape probability ε of horseflies, where the logistic curve is fitted to the dots showing the surface temperatures at which tabanids were escaped (ε = 100 %) and captured (ε = 0 %) within the 17 °C ≤ T_{barrel} ≤ 62 °C interval. A dotted curve illustrates the logistic fit to the data within the 31 °C ≤ T_{barrel} ≤ 55 °C interval. *Bottom*: Number N_c of captured horseflies versus T_{barrel}. The vertical light and medium grey columns denote the interval T_{min} = 31 °C ≤ T ≤ $T_{max,BBW}$ = 47 °C of the surface temperature of brown, beige and white (BBW) horses and the interval T_{min} = 31 °C ≤ T ≤ $T_{max,BL}$ = 55 °C of black (BL) horses measured by thermography (Fig. 2). (B) As A versus the air temperature T_{air}. (C) As A versus the difference T_{barrel} - T_{air}.

Click here to access/download
Supporting Information
+TabanidThermalTrapping_PLoS-One-supplement.doc

Revised Manuscript with Track Changes

Why Do Biting Horseflies Prefer Warmer Hosts? Tabanids can Escape Easier from Warmer Targets

Gábor Horváth[1]*, Ádám Pereszlényi[1,2], Ádám Egri[3,4], Tímea Tóth[1] and Imre M. Jánosi[5]

1: Department of Biological Physics, ELTE Eötvös Loránd University, H-1117 Budapest, Pázmány sétány 1, Hungary

2: Department of Zoology, Hungarian Natural History Museum, H-1083 Budapest, Ludovika tér 2-6., Hungary

3: MTA Centre for Ecological Research, Danube Research Institute, H-1113 Budapest, Karolina út 29-31, Hungary

4: MTA Centre for Ecological Research, Evolutionary Systems Research Group, Klebelsberg Kuno utca 3, H-8237 Tihany, Hungary

5: Department of Physics of Complex Systems, ELTE Eötvös Loránd University, H-1117 Budapest, Pázmány sétány 1, Hungary

*: correspondig author, e-mail: gh@arago.elte.hu

Corrections suggested by Referee 1 are marked by blue.

Corrections suggested by Referee 2 are marked by green.

Corrections suggested by Referee 3 are marked by orange.

Abstract

Blood-sucking horseflies (tabanids) prefer warmer (sunlit, darker) host animals and generally attack them in sunshine, the reason for which was unknown until now. Recently, it was hypothesized that blood-seeking female tabanids prefer elevated temperatures, because their wing muscles are quicker and their nervous system functions better at a warmer body temperature brought about by warmer microclimate, and thus they can more successfully avoid the host's parasite-repelling reactions by prompt takeoffs. To test this hypothesis, we studied in field experiments the success rate of escape reactions of tabanids landed on black targets as a function of the target temperature, and measured the surface temperature of differently coloured horses with thermography. We found that the escape success of tabanids decreased with decreasing target temperature, that is escape success is driven by temperature. Our results explain the behaviour of biting horseflies that they prefer warmer hosts against colder ones. Since in sunshine the darker the host the warmer its body surface, our results also explain why horseflies prefer sunlit dark (brown, black) hosts against bright (beige, white) ones, and why these parasites attack their hosts usually in sunshine, rather than under shady conditions.

Media Abstract

We proposed that horseflies prefer sunlit darker host animals, because they enjoy increased escape success on darker animals. We suggest that this is due to sunlit darker surfaces being warmer than brighter one, which in turn lets horseflies escape faster because of their increased muscle and nervous system performance. To test this hypothesis, we first measured the thermal properties of the surface of horses. This was then compared against the escape response of horseflies that had landed on barrels of different temperatures (in shade/sun, and/or containing warm air or cold water). We found that the escape response of horseflies increased with increasing barrel temperature, that is escape success is driven by temperature. Our finding explains why horseflies prefer sunlit darker hosts.

Keywords: Tabanidae; horsefly; thermoreception; warmth preference; host animal; parasite-host interaction

Introduction

Blood-sucking horseflies (tabanids) prefer warmer (sunlit, darker) host animals against colder (shady, brighter) ones and generally attack them in sunshine [1, 2, 3, 4, 5]. Tabanids attack black cattle more frequently than white ones [6]. Among white, brown and black cattle, black individuals are the preferred targets of *Tabanus* spp. horsefly attacks [7]. The attractiveness of sunlit brown horses to tabanids is about four times larger than that of sunlit white ones, and in comparison with a white horse, a brown horse spends two times longer period in a tabanid-free shady forest than in a sunny field with intense tabanid attacks [1]. The most effective tabanid traps use shiny black decoys [8, 9, 10, 11, 12, 13, 14, 15]. The so-called H-traps – composed of a bright tent with a shiny black sphere suspended below it – placed in sunny sites capture significantly more female tabanids than at shady sites [16]. The reason for this is that sunlit shiny dark targets reflect light at the Brewster's angle with higher degrees of linear polarization *d* than shady ones [17, 18], and host-seeking female tabanids prefer high *d*-values independent of the direction of polarization [19]. Thus, shiny black decoys used to catch horseflies work for their colour and reflected degree of polarization, rather than for their temperature.

After these experimental and observational findings concerning tabanid thermal preference, Horváth *et al.* [20] showed that *Tabanus tergestinus* horseflies prefer sunlit warm shiny black targets against sunlit or shady cold ones with the same optical characteristics. Furthermore, they hypothesized that a blood-seeking female tabanid prefers elevated temperatures, because her wing muscles are quicker and her nervous system functions better in a warmer microclimate, and thus she can more successfully avoid the host's parasite-repelling reactions by prompt takeoffs. Of course, there could also be other reasons why blood-sucking horseflies might prefer to attack warmer host animals. For example, to increase sweating, the capillaries could be enlarged near the epidermis of warmer hosts, which could be advantageous for blood-sucking insects.

The prediction of the hypothesis of Horváth *et al.* [20] is that the escape success of horseflies landed on host animals increases with increasing surface temperature. To test this prediction, we studied in field experiments the escape success of tabanids landed on black targets as a function of the surface temperature, and measured the coat temperature of differently coloured sunlit and shady horses with thermography. The results of our field experiments presented here corroborated the mentioned prediction which explains why blood-seeking horseflies prefer sunlit dark (warmer) host animals.

Results

As expected, the surface temperature T of the sunlit back of horses decreased in the colour order black > brown > beige > white, and the mean temperature $<T>$ of the bellies had smaller standard deviation ΔT than the backs (Fig. 1, Supplementary Figs. S1-S4). The minimum and maximum surface temperatures of horses were: black: 30.9-54.6 °C, brown: 31.2-44.6 °C, beige: 32.6-46.2 °C, white: 31.0-46.6 °C. The range T_{max} - T_{min} and ΔT increased with increasing $<T>$ (Fig. 2, Supplementary Tables S1-S4).

Figure 3 displays the surface temperature range $T_{min} \leq T \leq T_{max}$ of barrels and the percentages of escape success and capture rate of tabanids landed on the barrels under different illumination and thermal conditions. Considering experiments 1-3, the escape success was the highest on the sunlit air-filled barrel (85.4 %, $p < 0.001$, $\chi^2 = 48.167$, df = 1, significant), it was the lowest on the shady water-filled barrel (28 %, p = 0.02781, $\chi^2 = 4.84$, df = 1, significant), and on the shady air-filled barrel it was in between the former two (54.5 %, p = 0.5465, $\chi^2 = 0.36364$, df = 1, not significant). Under sunlit conditions in experiment 4, the escape success on the air-filled barrel (81.3 %) was definitely higher by a factor of 2.3 ($p < 0.001$, $\chi^2 = 34.9634$, df = 1) than that on the water-filled barrel (35.6 %). In experiment 5, tabanids could escape also with a significantly higher success ($p < 0.001$, $\chi^2 = 32.5403$, df = 1) from the sunlit side of the air-filled barrel (86.4 %) than from its shady side (39.5 %), similarly to the sunlit (45 %) and shady (29.4 %) sides of the water-filled barrel (p = 0.074398, $\chi^2 = 3.1832$, df = 1, non-significant). The numbers of captured and escaped tabanids were not significantly different in the following situations: shady side of the air-filled barrel in experiment 2, shady side of the air-filled barrel in experiment 5, and sunlit side of the water-filled barrel in experiment 5 (Fig. 3, Supplementary Table S5). According to Fig. 3 (Supplementary Tables S6-S10), the surface of air-filled barrels was always warmer than that of water-filled ones, and the sunlit surface of a given barrel was warmer than its shady side. All these results support our hypothesis that tabanids can escape more successfully from warmer targets than from cooler ones.

According to Fig. 4A, the number N_e of escaped tabanids landed on barrels has a maximum at around 41 °C, drops to zero at 17 °C and decreases almost to zero at 62 °C. The explanation of the drop of N_e at lower surface temperatures is that (i) less tabanids landed on colder surfaces, because tabanids flying through the cold boundary layer around the cold barrels could sense the lower surface temperature by their thermoreceptors (in the legs, antennae, mouthpart) and decided less frequently to alight, as Horváth *et al.* [20] showed, and (ii) the escape rate is lower at colder surfaces (see Fig. 4C). At higher surface temperatures more tabanids landed on the barrels, but because of the hot surface many of them flew away before the capturing attempt was performed in 10 seconds after landing (see Materials and Methods and Discussion). Therefore, the landing events shorter than 10 seconds were not registered, thus N_e droped with increasing T. In Fig. 4B, the number N_c of captured tabanids landed on barrels exhibits a clear decreasing trend with increasing T_{barrel} for both temperature intervals of 17 °C $\leq T \leq$ 62 °C and T_{min} = 31 °C $\leq T \leq T_{max,BL}$ = 55 °C. In Fig. 4C the increasing tendency of the normalized escape succes $e = N_e/(N_e + N_c)$ with increasing barrel's surface temperature T_{barrel} is clear. Figure 4C also illustrates that the systematic increase of the escape success e is also present in the temperature range T_{min} = 31 °C $\leq T \leq T_{max,BL}$ = 55 °C that is typical for the surface of horses.

We can see in Fig. 5A that the barrel's surface temperature T_{barrel} and the air temperature T_{air} correlate positively. According to Fig. 5B, there is a positive correlation between the normalized escape success $e = N_e/(N_e + N_c)$ and T_{air} if we take into consideration the results of all five experiments. Since T_{air} and T_{barrel} correlate positively (Fig. 5A) and e increases with increasing T_{barrel} (Fig. 4C), it could also be expected that e also increases with increasing T_{air} as seen in Fig. 5B. However, applying linear regression only for e-values measured at air temperatures lower than 33 °C (this way the warmest observations are eliminated when only sunlit air-filled warm barrels were used in experiment 1 resulting in a strong bias in the escape success e), the regressed line becomes horizontal as Fig. 5C displays. In this case there is no correlation between e and T_{air}. This suggests

that the landed horseflies spent enough time (10 seconds) on the barrel so that T_{barrel} determined the escape success, rather than T_{air}.

According to Fig. 5D, the normalized escape success *e* positively correlates with the temperature difference $\Delta T = T_{barrel} - T_{air}$ (°C) for all five experiments. Note that the 17-62 °C range of T_{barrel} is larger than the 23-38 °C range of T_{air}. This means that if ΔT is low/high, then T_{barrel} is also low/high. Thus, the result in Fig. 5D is similar to that in Fig. 4C, because only the temperature range (horizontal axis) was changed, which resulted in some blur due to the relatively small variation T_{air}.

In Fig. 6A the results of a logistic regression show a highly significant ($p < 0.0001$) positive correlation between the barrel's surface temperature T_{barrel} and the escape probability ε of landed tabanids (Supplementary Table S11). The effect of T_{barrel} in the logistic regression is also significant ($p = 0.000395$) for the temperature range 31-55 °C (Supplementary Table S12). The logistic regression in Fig. 6B displays a positive correlation between the air temperature T_{air} and the escape probability ε of tabanids, and the effect of T_{air} is highly significant ($p < 0.0001$, Supplementary Table S13). Figure 6C represents a highly significant ($p < 0.0001$) positive correlation between the difference $T_{barrel} - T_{air}$ and the escape probability ε of tabanids (Supplementary Table S14). These findings correspond to the results of the linear regressions.

Discussion

Female tabanids prefer to attack sunlit against shady dark host animals, and dark against bright hosts for a blood meal, the exact reasons for which were unknown. Our results presented here show that the surface temperature of sunlit darker horses is higher than that of sunlit brighter horses. This result corresponds to previous measurements [21, 22, 23, 24, 25, 26, 27]. The differences in surface temperatures of dark and bright as well as sunlit and shady hosts may partly explain their different attractiveness to tabanids. Horváth *et al.* [20] found that *Tabanus tergestinus* horseflies prefer sunlit warm shiny black targets against sunlit or shady cold ones with the same optical characteristics. They hypothesized that blood-sucking female tabanids prefer higher temperature, because their wing muscles are quicker and their nervous system functions better in a warmer microclimate, therefore they can avoid the parasite-repelling reactions of host animals by prompt takeoffs. Since the thermoreceptors of tabanids (as usually in Diptera) are in their legs, antennae and mouthpart [28, 29, 30], they cannot sense remotely (e.g. by infrareceptors) the temperature of a target. They can sense precisely the surface temperature of a substrate/host only after a physical contact (landing). However, on the basis of the leg/antenna/mouth-sensed temperature of the boundary layer around a target, tabanids can decide whether the target's surface is or is not warm enough for alighting [20].

The blood meal from warm-blooded animals is used by biting female horseflies as an energy source for maturation of their eggs [25, 28, 29, 30]. For this purpose, the blood of any warm-blooded hosts is good, regardless if hosts are dark- or bright-coloured, or shady or sunlit. In spite of this, blood-seeking female tabanids prefer dark and sunlit hosts [1, 2, 3, 4, 5, 6, 7], and this is the reason why horsefly traps usually have black decoys and are most effective in sunshine [8, 9, 10, 11, 12, 13, 14, 15, 16]. Our main assumption was that blood-seeking tabanids prefer to land on sunlit dark hosts to keep their body warm, which aids their rapid escape when the host performs such typical antiparasite reactions as removing horseflies from their coats with tails brushing them off, by stamping and dislodging the flies, or by nibbling their skin, for instance [1, 30]. These fly-repelling reactions are dangerous for blood-sucking tabanids, therefore have to be avoided by a quick flying away.

In this work we analysed the results of our field experiments for the whole tabanid population of the study area without considering differences between tabanid species/genus, because species/genus identification was not feasible in the field. However, there may be differences between species/genus in landing, daily activity and responses to environmental parameters that might influence their escape success. For example, Haematopota species might be more active in

late afternoon when air temperature decreases. It is also unknown whether the influence of air temperature T_{air} on host preference for *Haematopota* sp. is lower than that for *Tabanus* or *Atylotus* sp. To test these hypotheses could be the task of further studies. What we know from our fields experiments is the following: (i) Apart from T_{air} (Supplementary Tables S6-S10) the weather situation (calm with no meteorological fronts) was the same during our field experiments. (ii) There was no correlation between the escape success e of landed horseflies and $T_{air} < 33$ °C (Fig. 5C). In this case the barrel temperature T_{barrel} determined the escape success e, rather than T_{air}.

According to earlier field experiments [31, 32] in the same experimental site (a Hungarian horse farm in Szokolya) with the same tabanid species (*Tabanus tergestinus*, *T. bromius*, *T. bovinus*, *T. autumnalis*, *Atylotus fulvus*, *A. loewianus*, *A. rusticus*, *Haematopota italica*) as in the case of our present field experiments, the daily activity of different tabanid species and the effect of weather variables on their flight activity are slightly different. Herczeg *et al*. [32], for example, found the following: (i) Rainfall, air temperature, and sunshine were the three most important factors influencing the trapping number of tabanids. (ii) The effect of relative air humidity H on tabanids was indirect through the air temperature T: $H \approx 35$ % (corresponding to $T \approx 32$ °C) was optimal for tabanid trapping, and tabanids were not captured for $H \geq 80$ % (corresponding to $T \leq 18$ °C). (iii) A fast decrease in the air pressure enhanced the trapping number of horseflies. (iv) Wind velocities larger than 10 km/h reduced drastically the number of trapped tabanids.

In our field experiments 4 and 5 warm and cold sunlit barrels were used simultaneously, while in experiments 1-3 the cold and the warm barrels were tested separately (experiment 1: sunlit air-filled barrels, experiment 2: shady air-filled barrels, experiment 3: shady water-filled barrels). This was, however, not a problem, because apart from the air temperature (Supplementary Tables S6-S10) the environmental conditions (calm with no meteorological fronts) were practically the same on all experimental days. Thus, the slightly different environmental factors in our field experiments could have resulted in only small differences in the activity of a given tabanid species.

In our field experiments the investigator waited 10 seconds before he tried to capture a tabanid landed on a barrel. This 10-second period turned out to be optimal: it was neither too short, nor too long. Within a period shorter than 10 s the flight muscles of landed tabanids could not warm up or cool down to the surface temperature of barrels. On the other hand, when a landed tabanid recognizes that the barrel is not a host animal, it flies away after a certain period. If the waiting period of counting of landed tabanids were too long, then many landing events would not be counted/registered. The air temperature did not correlate with the escape rate (Fig. 5C), therefore 10 s was long enough to warm the wing muscles of flies landed on the barrel. On the other hand, if a tabanid lands on a sunlit dark (warm) surface, then it does not cool down, contrary to a bright (colder) surface where its wing muscles can cool down. It is pertinent to suppose that during flight the wing muscles are appropriately warm. Furthermore, according to Heinrich [33], many Dipteran species (including true flies and also tsetse flies) can be heterothermic, or generate certain amount of heat which can then be used to improve their performance. However, as far as we know, practically nothing is known about the thermoregulation in horseflies. Thus, without knowing the horsefly thermal physiology, we restricted our study to the correlation between (air/barrel) temperature and escape success with a waiting period of 10 seconds before the experimenter tried to capture a tabanid landed on a barrel.

During the field experiments all barrels were optically uniformly attractive to host-seeking horseflies. Our air-filled sunlit warm barrels (37-62 °C) thermally imitated sunlit black horses (31-55 °C), while our water-filled sunlit cold barrels (19-29 °C) were cooler than sunlit brown, beige and white horses (31-47 °C). Although the measured low escape successes in the case of our water-filled cold barrels were associated with temperatures that were lower than those on the studied horse bodies, the positive correlation between escape success e and surface temperature T (i.e. increasing e with increasing T) is also evident in the interval $T_{min} = 31$ °C $\leq T \leq T_{max,BL} = 55$ °C as clearly shown by the dotted curves of Figs. 4C and 6A. Thus, the results of our field experiments show that the escape success of tabanids depends on the host's surface temperature T: the higher the T, the

larger the escape success of horseflies. The average surface temperature of the studied sunlit black horses was between 48 and 55 °C. Almost all tabanids managed to escape in this temperature range.

In Figs. 4 and 6A, the 100 % escape successes at surface temperatures $T > 50$ °C are associated with very small absolute numbers of horseflies and also with hot air temperatures ranging from 33 to 38 °C (Supplementary Table S6). The reason for this is that only the minority of tabanids landing on hot ($T > 55$ °C) surfaces spent periods longer than 2 seconds on them, and the waiting time until a catch attempt was 10 seconds after a tabanid landed on a barrel. In experiment 1 the weather was very warm ($T_{air} \leq 38$ °C) and the surface temperature of the warm barrel was above 45 °C the whole day (Supplementary Table S6). Larger horseflies can tolerate extreme temperatures for a short time, and are also intrinsically faster [30].

The thermal transfer between a horsefly and a surface could be very different in the following two cases: (i) When a fly lands on a horse, the grip is maintained by the insect's legs holding on the hairs, leaving plenty of insulating air between the two animals. (ii) When a fly lands on a plastic barrel, its legs must remain attached to a much less convenient substrate and the thermal transfer could be different (perhaps more efficient on plastic). In the future, an important task could be to measure both thermal transfers.

It has been shown that dark-bodied host animals have a much stronger reflection-polarization signature than bright-bodied ones, which is an important visual sign, leading to horsefly attacks [18]. The high degrees of polarization of reflected light helps tabanids to select sunlit dark host animals from the dark patches of their visual environment. A strong polarization signature could also advertise a hot animal and greater chances of escape of tabanids, but both traits could be also dissociated, all depending on the thermal transfer and the thermal physiology of horseflies.

Conclusion

We presented here the results of our field experiment studying the dependence of tabanid escape success on the temperature of the landing surface. The temperature of artificial landing sites was parallelled by the analysis of thermal imaging measurements of surface temperatures in different body parts of differently coloured horses. Not surprisingly, fur temperatures were higher in darker horses and lower in bright coloured ones. The tabanid escape success strongly depended on the surface temperature; the highest escape success occurred on surfaces having temperatures similar to those recorded in black horses, i.e. above 50 °C. We drew the conclusion that the warmer (also darker) host animals allow higher escape success of blood-sucking horseflies. This supports our hypothesis that the preference of horseflies to dark hosts has partly evolved due to higher survival rate.

Materials and Methods

Animal Ethics Statement and Field Study Permits

Csaba Viski permitted us to photograph his horses in his horse farm in Szokolya. For the location and activities of our field study no specific permissions were required.

Thermography of horses

On a warm day (6 July 2019) 46 thermograms of 2 black, 2 brown, 2 beige and 2 white horses were measured with an infrared camera (VarioCAM®, Jenoptik Laser Optik Systeme GmbH, Jena, Germany, nominal precision of ±1.5 °C) under sunny and cloudy conditions. The validation and calibration of this thermocamera with a contact thermometer (GAO Digital Multitester EM392B 06554H, EverFlourish Europe Gmbh., Friedrichsthal, Germany, nominal precision of ±1 °C) were described in the Supporting Material of [20].

Field experiments

Field experiments 1-5 were performed on 1, 2, 3, 4 and 11 July 2019 on a Hungarian horse farm in Szokolya (47° 52' North, 19° 00' East), where horseflies were present. All five experimental days were windless, and only weak local winds blew in early afternoons. Meteorological fronts did not move through the site of experiments. Thus, apart from the air temperature (according to Supplementary Tables S6-S10, in experiment 1 the air temperatures were warmer than on the other four experimental days), the environmental conditions were practically the same. In mornings, the weather was sunny, warm and cloudless, however in the afternoons a few cumulus clouds formed. In these experiments the escape success of tabanids landed on shiny black cylindrical plastic barrels (height = 42 cm, diameter = 30 cm, wall thickness = 5 mm) of different surface temperatures (set with warm air or cold water load) but with the same optical characteristics was studied under sunlit and shady conditions. The purpose of these barrels was to imitate warm and cold dark host animals of tabanids. The high enough temperature differences between the warm and cold barrels could easily be ensured with air-filled warm and water-filled cold barrels. An experimenter, who was "blind" to the predictions of the experiment tried to capture the tabanids landed on the barrels with a hemispherical tea-strainer of diameter 15 cm. The waiting time until a catch attempt was 10 seconds (measured with a stopwatch) after a tabanid landed on a barrel (the choice of this optimal 10-second value is explained in the Discussion).

- In **experiment 1** (1 July 2019, 10:20-17:00 hour = local summer time = UTC + 2 h) two air-filled sunlit black barrels were used, which thermally modelled sunlit black host animals (e.g. horses) for tabanids. The two barrels were put on top of each other, and both were placed on a four-legged white plastic stand (height = 46 cm) at a sunlit site without any shade cast by vegetation or other objects. Only tabanids landed on the sunlit side of the barrels were taken into account.
- In **experiment 2** (2 July 2019, 9:40-16:00 h) two air-filled barrels under shadow were used, which modelled shady hosts. The barrels were put on top of each other and the white stand was in the shade of trees during the experiment. Only tabanids landed on the side of the barrels facing toward the open field were considered.
- In **experiment 3** (3 July 2019, 9:50-16:00 h) two cold-water-filled shady barrels were used, which modelled cool shady hosts. The barrels were continuously under the shadow of trees. Both barrels were filled with tap water and 10 frozen ice packs (Aspico G40, 0.25 litre, 0.76 kg). The experimenter tried to capture only tabanids landed on the side of the barrels facing toward the open field.
- In **experiment 4** (4 July 2019, 10:00-12:00 h) two sunlit air-filled barrels and two sunlit cold-water-filled barrels were used which thermally modelled sunlit and shady hosts, respectively. Both barrels were continuously exposed to sunlight. Only tabanids landed on the sunlit side of the barrels were tried to capture.
- **Experiment 5** (11 July 2019, 10:20-16:00 h) was technically the same as experiment 4, but all tabanids landed on both sunlit and shady sides of barrels were subject of attack.

The experimenter wore white clothes and hat against direct sunshine and to minimize his visual attractiveness to tabanids. He was sitting on a chair during the experiments next to the barrels (50 cm) in such a way that he could easily reach the tabanids on the barrels with the tea-strainer. After the fly was successfully caught, it was released. After each capture trial the air temperature and the surface temperature of the barrel at the tabanid's landing location was measured by a contact thermometer (GAO Digital Multitester EM392B 06554H, EverFlourish Europe Gmbh., Friedrichsthal, Germany, nominal precision of ±1 °C). For this purpose, thermography was not possible to use because of technical problems. The experimenter was the same person throughout all experiments, who has practiced himself in tabanid capture during a pilot experiment. In the

vicinity of the barrels there were no many flying tabanids. Threfore, only one tabanid landed on one of the two (warm or cold) barrels at a given time, thus the experimenter did not have to split his attention between two or more landed tabanids.

An *in situ* identification of the species of tabanids landed on the barrels was not feasible. It was obvious, however, that they were tabanids (Diptera: Tabanidae). In previous field experiments [31, 32], the following tabanid species occurred at the same study site: *Tabanus tergestinus*, *T. bromius*, *T. bovinus*, *T. autumnalis*, *Atylotus fulvus*, *A. loewianus*, *A. rusticus*, *Haematopota italica*. Since we could record the escape success of different tabanid species, our results can be considered as the average escape success of the tabanid population of the experimental site.

Since the reflection-polarization characteristics of the dry barrel's surface are independent of its temperature in the visible spectral range, all optical parameters (radiance, degree of linear polarization and angle of polarization) of our warm and cool barrels were the same.

Statistical analysis

For comparison of the numbers of escaped and captured tabanids landed on test surfaces of various temperatures, we applied χ^2 tests of homogeneity, where the escape versus non-escape ratio was tested against the predicted 50/50 ratio. These χ^2 tests were performed to compare escape/non-escape numbers for a given barrel or barrel side (sunlit or shady). Thus, the compared escape/non-escape numbers corresponded to the same barrel temperature and there was no comparison between data originating from different barrel temperatures. In other words, χ^2 test was used to detect whether a given barrel temperature had an effect on tabanid escape success.

Linear regressions were applied to find a trend of the escape success of horseflies as a function of the surface temperature T_{barrel}, air temperature T_{air} and the temperature difference T_{barrel} - T_{air} between the barrel surface and the air. We also applied logistic regression to model the probability of escape as a function of T_{barrel}, T_{air} and T_{barrel} - T_{air}. We also applied the linear and logistic regressions as a funciton of T_{barrel} using the data within the 31 °C ≤ T ≤ 55 °C interval. Logistic regression was also used to find whether there is a correlation between T_{air} and T_{barrel}. The R statistical package 3.0.2 [34] was used for statistical analyses.

Copyrighted Figures

There is no any element of our submission (e.g. figures, supporting information, striking image) under copyright. In our manuscript nothing has been adapted from copyrighted material.

Supporting Information

Our paper has the following electronic supporting materials: Supplementary Tables S1-S11, Supplementary Figures S1-S4.
(DOC)

Supplementary Table S1: Temperatures of black horses measured with thermography on shady and sunlit sides of the back and belly, and when the sun was occluded by clouds (cloudy). <*T*>: average, ±Δ*T*: standard deviation, T_{min}: minimum, T_{max}: maximum.
(DOC)

Supplementary Table S2: Temperatures of brown horses measured with thermography on shady and sunlit sides of the back and belly, and when the sun was occluded by clouds (cloudy). <*T*>: average, ±Δ*T*: standard deviation, T_{min}: minimum, T_{max}: maximum.
(DOC)

Supplementary Table S3: Temperatures of beige horses measured with thermography on shady and sunlit sides of the back and belly, and when the sun was occluded by clouds (cloudy). $<T>$: average, $\pm\Delta T$: standard deviation, T_{min}: minimum, T_{max}: maximum.
(DOC)

Supplementary Table S4: Temperatures of white horses measured with thermography on shady and sunlit sides of the back and belly, and when the sun was occluded by clouds (cloudy). $<T>$: average, $\pm\Delta T$: standard deviation, T_{min}: minimum, T_{max}: maximum.
(DOC)

Supplementary Table S5: Results of χ^2 tests comparing the sums of Supplementary Tables S6-S10 obtained in experiments 1-5.
(DOC)

Supplementary Table S6: Capture success (-: not captured, +: captured) of horseflies, and temperatures of the air (T_{air}) and the surface of the air-filled sunlit barrel (T_{barrel}) in experiment 1 on 1 July 2019.
(DOC)

Supplementary Table S7: Capture success (-: not captured, +: captured)) of horseflies, and temperatures of the air (T_{air}) and the surface of the air-filled shady barrel (T_{barrel}) in experiment 2 on 2 July 2019.
(DOC)

Supplementary Table S8: Capture success (-: not captured, +: captured) of horseflies, and temperatures of the air (T_{air}) and the surface of the cold-water-filled shady barrel (T_{barrel}) in experiment 3 on 3 July 2019.
(DOC)

Supplementary Table S9: Capture success (-: not captured, +: captured) of horseflies, and temperatures of the air (T_{air}) and the sunlit side of the surface of the air-filled warm barrel (T_{warm}) and the water-filled cold barrel (T_{cold}) in experiment 4 on 4 July 2019.
(DOC)

Supplementary Table S10: Capture success (-: not captured, +: captured) of horseflies, and temperatures of the air (T_{air}) and the surface of the air-filled sunlit barrel (T_{warm}) and the cold-water-filled sunlit barrel (T_{cold}) in experiment 5 on 11 July 2019.
(DOC)

Supplementary Table S11. Summary of the logistic regression. The escape probability ε of tabanids depends highly significantly on the barrel's surface temperature T_{barrel} in the interval 17 °C $\leq T_{barrel} \leq$ 62 °C. The large difference between the null deviance and the residual deviance suggests that the logistic regression model is accurate.
(DOC)

Supplementary Table S12. Summary of the logistic regression. The escape probability ε of tabanids depends highly significantly on the barrel's surface temperature T_{barrel} in the interval 31 °C $\leq T_{barrel} \leq$ 55 °C.
(DOC)

Supplementary Table S13. Summary of the logistic regression. The escape probability ε of tabanids depends highly significantly on the air temperature T_{air}.

(DOC)

Supplementary Table S14. Summary of the logistic regression. The escape probability ε of tabanids depends highly significantly on the temperature difference T_{barrel} - T_{air}.
(DOC)

Supplementary Figure S1. Photographs, thermograms and thermograms with selected back and belly areas of black horses under different illumination conditions. shady: shady side of the sunlit horse. sunlit: sunlit side of the sunlit horse. cloudy: illuminated by skylight when the sun was occluded by clouds.
(TIF)

Supplementary Figure S2. Photographs, thermograms and thermograms with selected back and belly areas of brown horses under different illumination conditions. shady: shady side of the sunlit horse. sunlit: sunlit side of the sunlit horse. cloudy: illuminated by skylight when the sun was occluded by clouds.
(TIF)

Supplementary Figure S3. Photographs, thermograms and thermograms with selected back and belly areas of a beige horse under different illumination conditions. shady: shady side of the sunlit horse. sunlit: sunlit side of the sunlit horse. cloudy: illuminated by skylight when the sun was occluded by clouds.
(TIF)

Supplementary Figure S4. Photographs, thermograms and thermograms with selected back and belly areas of white horses under different illumination conditions. shady: shady side of the sunlit horse. sunlit: sunlit side of the sunlit horse. cloudy: illuminated by skylight when the sun was occluded by clouds.
(TIF)

Author contributions

Substantial contributions to conception and design: GH, ÁE, ÁP
Performing experiments and data acquisition: GH, ÁP, TT, IMJ
Data analysis and interpretation: GH, ÁP, TT, IMJ
Drafting the article or revising it critically for important intellectual content: GH, ÁE, ÁP, IMJ

Competing interests

The authors have no competing interests.

Funding

This work was supported by the grant NKFIH K-123930 (Experimental Study of the Functions of Zebra Stripes: A New Thermophysiological Explanation) received by Gábor Horváth from the Hungarian National Research, Development and Innovation Office. Ádám Egri was supported by the Economic Development and Innovation Operational Programme (GINOP-2.3.2-15-2016-00057), the grant NKFIH PD-131738 and the János Bolyai Research Scholarship of the Hungarian Academy of Sciences. The funders had no role in study design, data collection and analysis, decision to publish, or preparation of the manuscript.

Acknowledgements

We are grateful to Csaba Viski who permitted the performance of our field experiments in his horse farm in Szokolya. We thank for the valuable and constructive comments of three anonymous reviewers.

References

1. Horváth G, Blahó M, Kriska G, Hegedüs R, Gerics B, Farkas R, Åkesson S. An unexpected advantage of whiteness in horses: the most horsefly-proof horse has a depolarizing white coat. Proceedings of the Royal Society B 2010; 277: 1643-1650.

2. Blahó M, Egri Á, Báhidszki L, Kriska G, Hegedüs R, Åkesson S, Horváth G. Spottier targets are less attractive to tabanid flies: on the tabanid-repellency of spotty fur patterns. Public Library of Science One 2012; 7: e41138 (doi: 10.1371/journal.pone.0041138).

3. Blahó M, Egri Á, Száz D, Kriska G, Åkesson S, Horváth G. Stripes disrupt odour attractiveness to biting horseflies: Battle between ammonia, CO_2, and colour pattern for dominance in the sensory systems of host-seeking tabanids. Physiology and Behavior 2013; 119: 168-174.

4. Egri Á, Blahó M, Kriska G, Farkas R, Gyurkovszky M, Åkesson S, Horváth G. Polarotactic tabanids find striped patterns with brightness and/or polarization modulation least attractive: an advantage of zebra stripes. Journal of Experimental Biology 2012; 215: 736-745.

5. Krcmar S, Radolic V, Lajos P, Lukacevic I. Efficiency of colored modified box traps for sampling tabanids. Parasite 2014; 21: 67 (doi: 10.1051/parasite/2014068).

6. Tashiro H, Schwardt HH. Biological studies of horse flies in New York. Journal of Economical Entomology 1953; 46: 813-822.

7. Lendzele SS, Eisenbarth A, Christophe ZKR, Mavoungou JF, Renz A. Aspects of the bionomics of hematophagous symbovine dipterans in a hyper-infested rangeland of Ngaoundere (Adamawa-Cameroon). Journal of Asia-Pacific Entomology 2019; 22: 1019-1030.

8. Thorsteinson AJ, Bracken GK, Tostawaryk W. The orientation behaviour of horse flies and deer flies (Tabanidae: Diptera). V. The influence of the number of reflecting surfaces on attractiveness to tabanids of glossy black polyhedra. Canadian Journal of Zoology 1966; 44: 275-279.

9. Catts EP. A canopy trap for collecting Tabanidae. Mosquito News 1970; 30: 472-474.

10. Roberts RH. Attractancy of two black decoys and CO_2 to tabanids (Diptera: Tabanidae). Mosquito News 1977; 37: 169-172.

11. Wall WJ, Doane OW. Large scale use of box traps to study and control saltmarsh greenhead flies (Diptera: Tabanidae) on Cape Cod, Massachusetts. Environmental Entomology 1980; 9: 371-375.

12. Blahó M, Egri Á, Barta A, Antoni G, Kriska G, Horváth G. How can horseflies be captured by solar panels? A new concept of tabanid traps using light polarization and electricity produced by photovoltaics. Veterinary Parasitology 2012; 189: 353-365.

13. Egri Á, Blahó M, Száz D, Barta A, Kriska G, Antoni G, Horváth G. A new tabanid trap applying a modified concept of the old flypaper: Linearly polarising sticky black surfaces as an effective tool to catch polarotactic horseflies. International Journal for Parasitology 2013; 43: 555-563.

14. Egri Á, Blahó M, Száz D, Kriska G, Majer J, Herczeg T, Gyurkovszky M, Farkas R, Horváth G. A horizontally polarizing liquid trap enhances the tabanid-capturing efficiency of the classic canopy trap. Bulletin of Entomological Research 2013; 103: 665-674.

15. Meglic A, Ilic M, Pirih P, Skorjanc A, Wehling MF, Kreft M, Belusic G. Horsefly object-directed polarotaxis is mediated by a stochastically distributed ommatidial subtype in the ventral retina. Proceedings of the National Academy of Sciences of the United States of America 2019; 116: 21843-21853.

16. Otártics MZ, Altbäcker V, Solymosi K, Mátics R, Romvári R, Farkas S. Efficacy of H-traps is affected by exposure to sunshine. Natura Croatica 2019; 28: 257-269.

17. Horváth G. (editor) 2014; Polarized Light and Polarization Vision in Animal Sciences. Springer: Heidelberg, Berlin, New York

18. Horváth G., Szörényi T., Pereszlényi Á., Gerics B., Hegedüs R., Barta A., Åkesson S. Why do horseflies need polarization vision for host detection? Polarization helps tabanid flies to select sunlit dark host animals from the dark patches of the visual environment. Royal Society Open Science 2017; 4: 170735 (doi: 10.1098/rsos.170735)

19. Egri Á, Blahó M, Sándor A, Kriska G, Gyurkovszky M, Farkas R, Horváth G. New kind of polarotaxis governed by degree of polarization: attraction of tabanid flies to differently polarizing host animals and water surfaces. Naturwissenschaften 2012; 99: 407-416.

20. Horváth G, Pereszlényi Á, Tóth T, Polgár S, Jánosi IM. Attractiveness of thermally different uniformly black targets to horseflies: *Tabanus tergestinus* prefers sunlit warm shiny dark targets. Royal Society Open Science 2019; 6: 191119 (doi: 10.1098/rsos.191119).

21. Cena K. Investigations of absorption of solar radiation by cattle and horses with various coat colours. Acta Agraria et Silvestria 1966; 6: 93-138.

22. Cena K, Clark JA. Thermographic measurements of the surface temperatures of animals. Journal of Mammalogy 1973; 54: 1003-1007.

23. Benesch AR, Hilsberg S. Infrarot-thermographische Untersuchungen der Oberflächentemperatur bei Zebras. Zoologischer Garten 2003; NF 2: 74-82.

24. Benesch AR, Hilsberg-Merz S. Oberflächentemperaturen bei Zebrastreifen. Natur und Museum 2006; 136: 49-56.

25. Caro T. Zebra Stripes. 2016; University of Chicago Press: Chicago.

26. Horváth G, Pereszlényi Á, Száz D, Barta A, Jánosi IM, Gerics B, Åkesson S. Experimental evidence that stripes do not cool zebras. Scientific Reports 2018; 8: 9351 (doi: 10.1038/s41598-018-27637-1).

27. Cobb A, Cobb S. Do zebra stripes influence thermoregulation? Journal of Natural History 2019; 53: 863-879.

28. McGavin GC. Essential entomology: an order-by-order introduction. 2001; Oxford University Press: Oxford.

29. Elzinga RJ. Fundamentals of Entomology. 2003; 6th ed. Upper Saddle River, NJ: Prentice Hall.

30. Lehane MJ. The biology of blood-sucking in insects. 2005; 2nd ed, Cambridge University Press: Cambridge, UK.

31. Herczeg T, Blahó B, Száz D, Kriska G, Gyurkovszky M, Farkas R, Horváth G. Seasonality and daily activity of male and female tabanid flies monitored in a Hungarian hill-country pasture by new polarization traps and traditional canopy traps. Parasitology Research 2014; 113: 4251-4260.

32. Herczeg T, Száz D, Blahó B, Barta A, Gyurkovszky M, Farkas R, Horváth G. The effect of weather variables on the flight activity of horseflies (Diptera: Tabanidae) in the continental climate of Hungary. Parasitology Research 2015; 114: 1087-1097.

33. Heinrich B. The Hot-Blooded Insects: Strategies and Mechanisms of Thermoregulation. 1993; Springer: Heidelberg, Berlin, New York

34. R Core Team. R: a language and environment for statistical computing. R Foundation for Statistical Computing, 2013 Vienna http://www.R-project.org/.

Figures with Captions

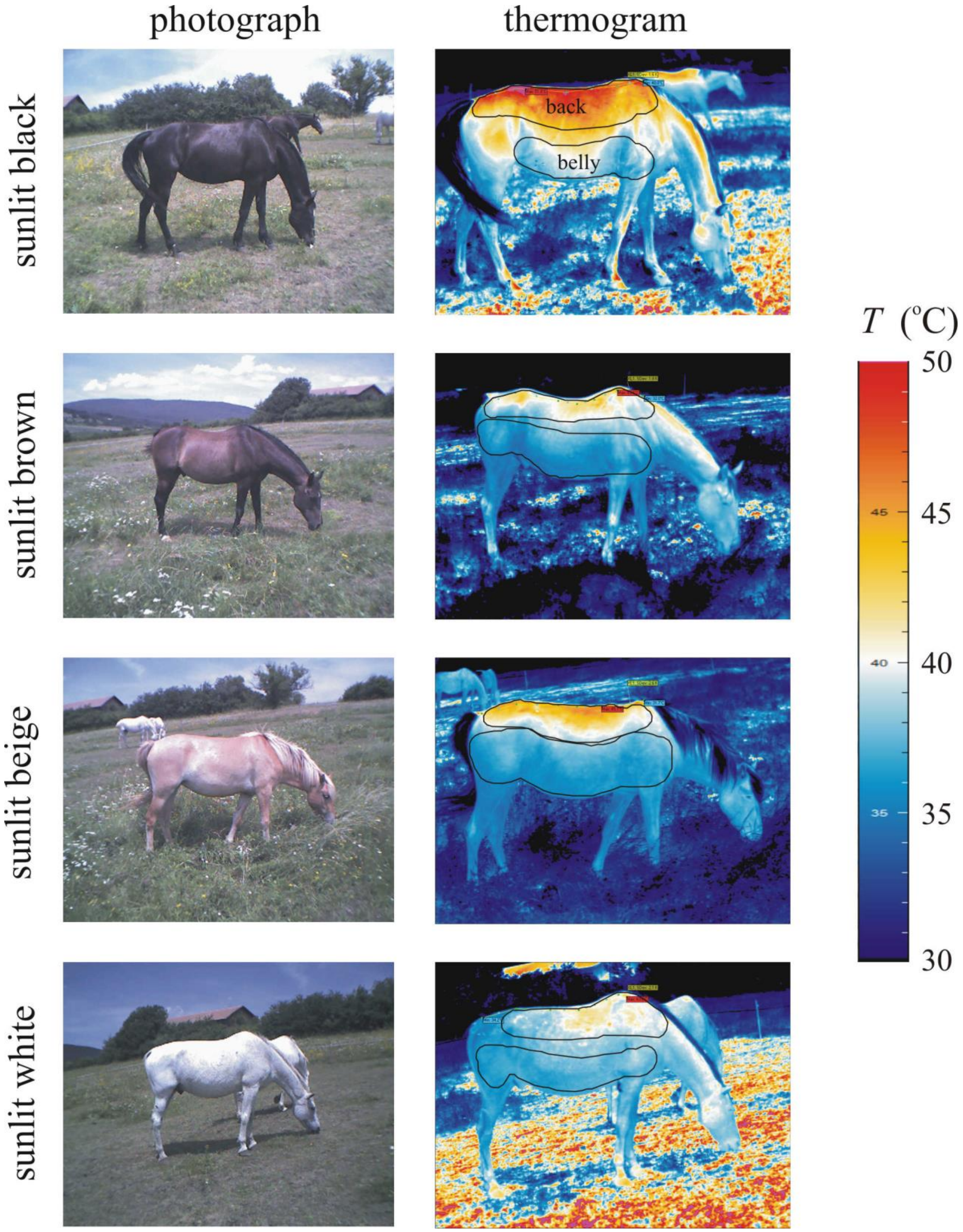

Figure 1. Thermograms of horses. Photographs and thermograms of sunlit black, brown, beige and white horses. In the thermograms the black perimeters of the back and belly areas are shown where the surface temperature T was averaged.

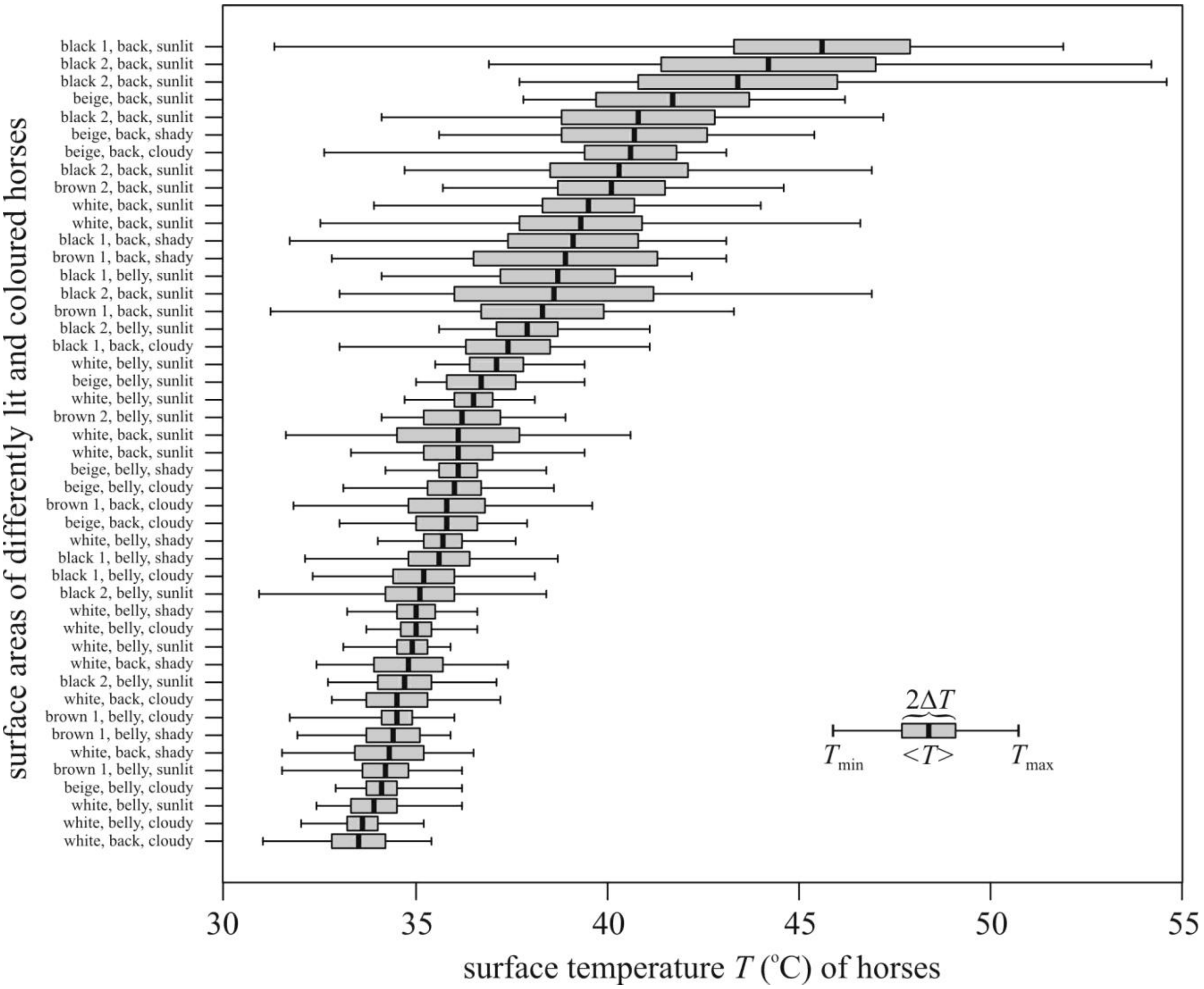

Figure 2. Surface temperatures of horses. Minimum (T_{min}), maximum (T_{max}), average ($<T>$) and standard deviation (ΔT) of the surface temperature of the back and belly of black, brown, beige and white horses measured with thermography under different illumination conditions (Supplementary Tables S1-S4, Supplementary Figs. S1-S4). shady: shady side of a sunlit horse, sunlit: sunlit side of a sunlit horse, cloudy: the horse was illuminated by skylight when the sun was occluded by clouds.

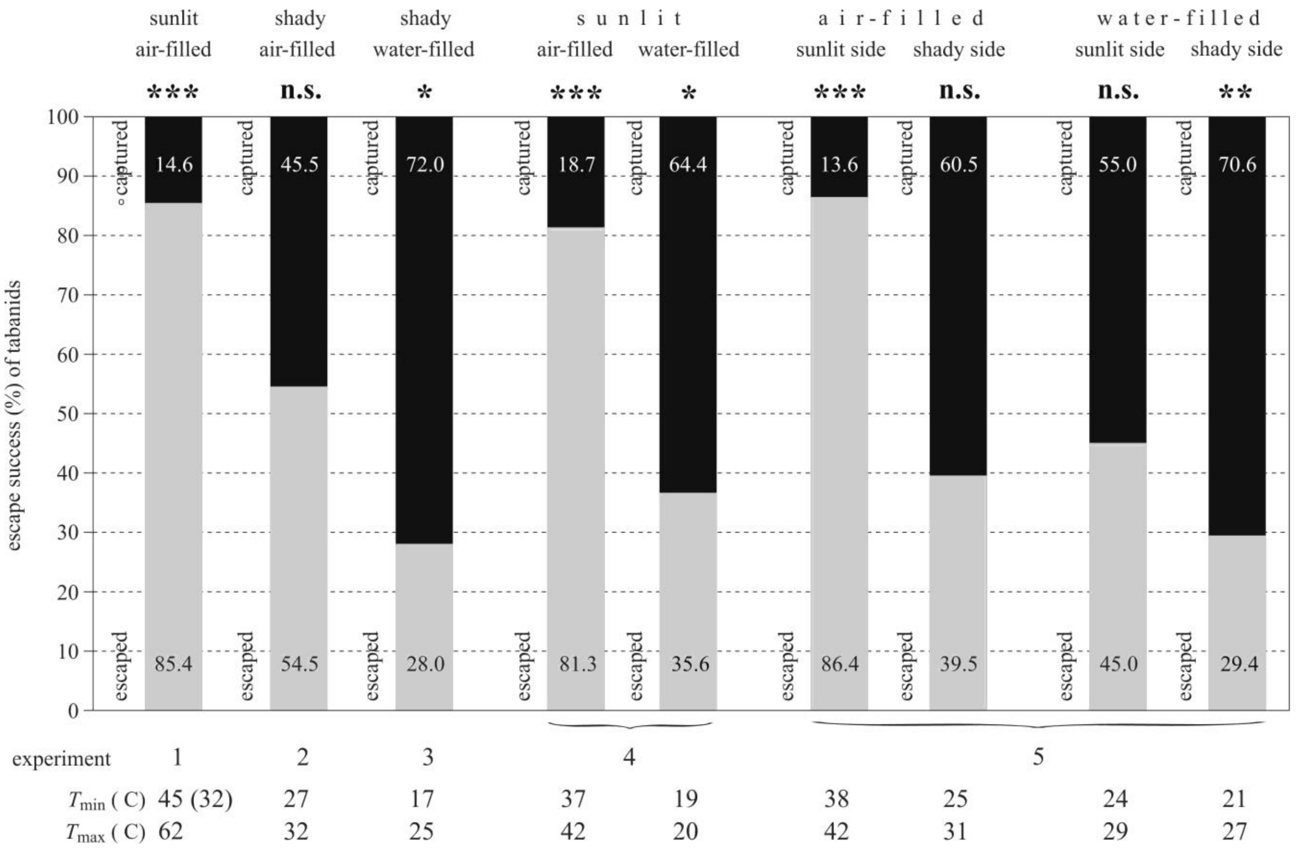

Figure 3. Escape success of horseflies in experiments 1-5. Surface temperature range $T_{min} \leq T \leq T_{max}$ of barrels and the percentage of escape success of tabanids (grey bars) landed on the barrels under different illumination and thermal conditions in experiments 1-5 (Supplementary Tables S6-S10). The results of χ^2 test are indicated: n.s: not significant, p > 0.05, *: 0.01 < p < 0.05, **: 0.001 < p < 0.01, ***: p < 0.001 (Supplementary Table S5). Grey and black bars illustrate the escape and capture rates, the percentage values of which are given in the columns.

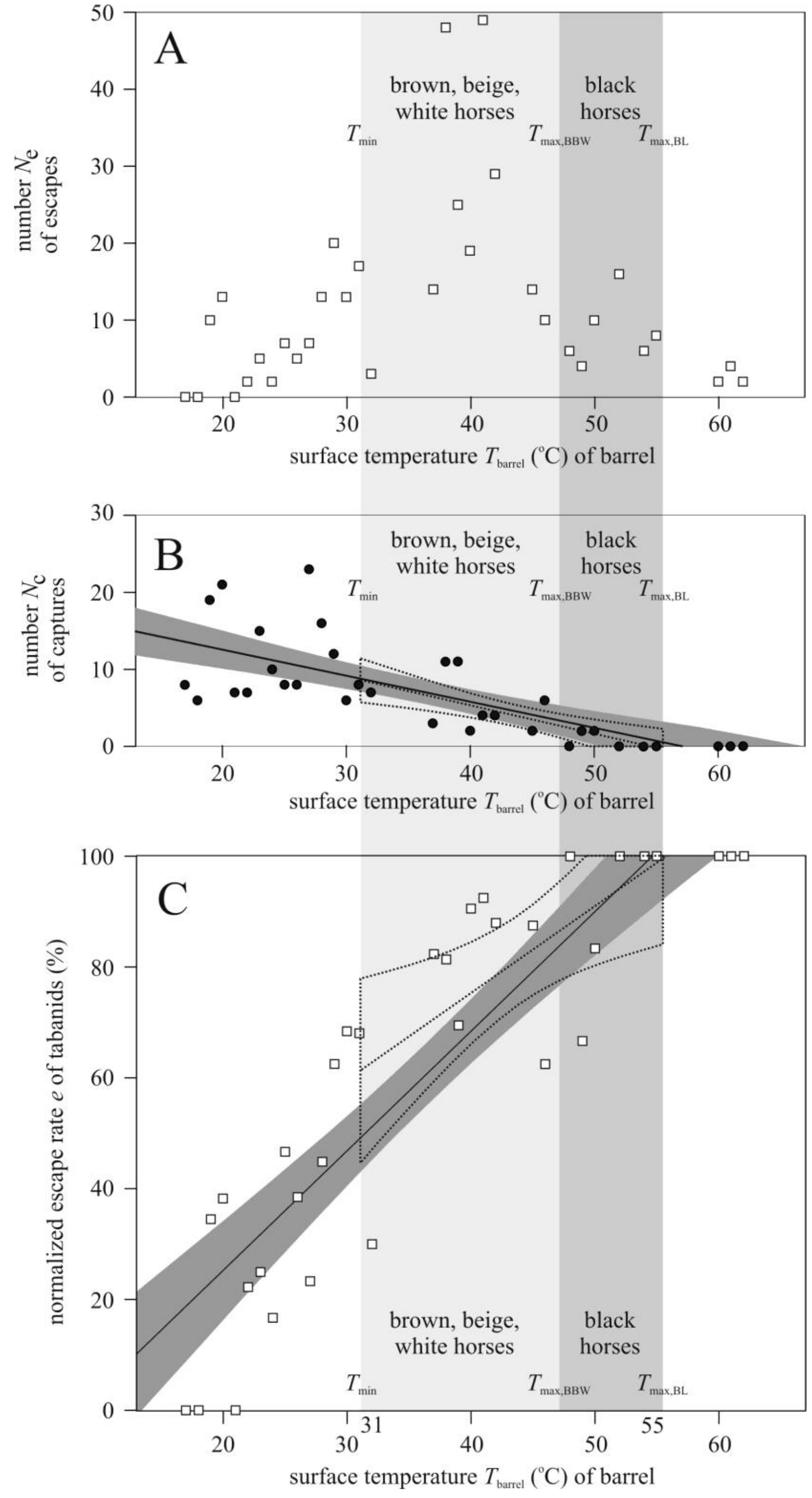

Figure 4. Escape success of horseflies versus surface temperature. Number N_e of escaped (A) and number N_c of captured (B) tabanids landed on barrels as a function of the surface temperature T_{barrel} (°C). In B a continuous straight line indicates the linear fit to all $N_c(T_{barrel})$ data (black circles in the interval 17 °C $\leq T \leq$ 62 °C), and a dark grey band around this fit shows the 95 % confidence interval. (C) Normalized escape success $e = N_e/(N_e + N_c)$ versus T_{barrel} (°C). A continuous straight line and a dark grey band illustrate the linear fit to all $e(T_{barrel})$ data (white squares in the interval 17 °C $\leq T \leq$ 62 °C) with 95 % confidence interval. The vertical light and medium grey columns denote the interval T_{min} = 31 °C $\leq T \leq T_{max,BBW}$ = 47 °C of the surface temperature of brown, beige and white horses (BBW) and the interval T_{min} = 31 °C $\leq T \leq T_{max,BL}$ = 55 °C of black (BL) horses measured by thermography (Fig. 2). In B and C a dotted straight line and a 95 % confidence interval with dotted perimeter illustrate the linear fit to the data within the 31 °C $\leq T \leq$ 55 °C interval.

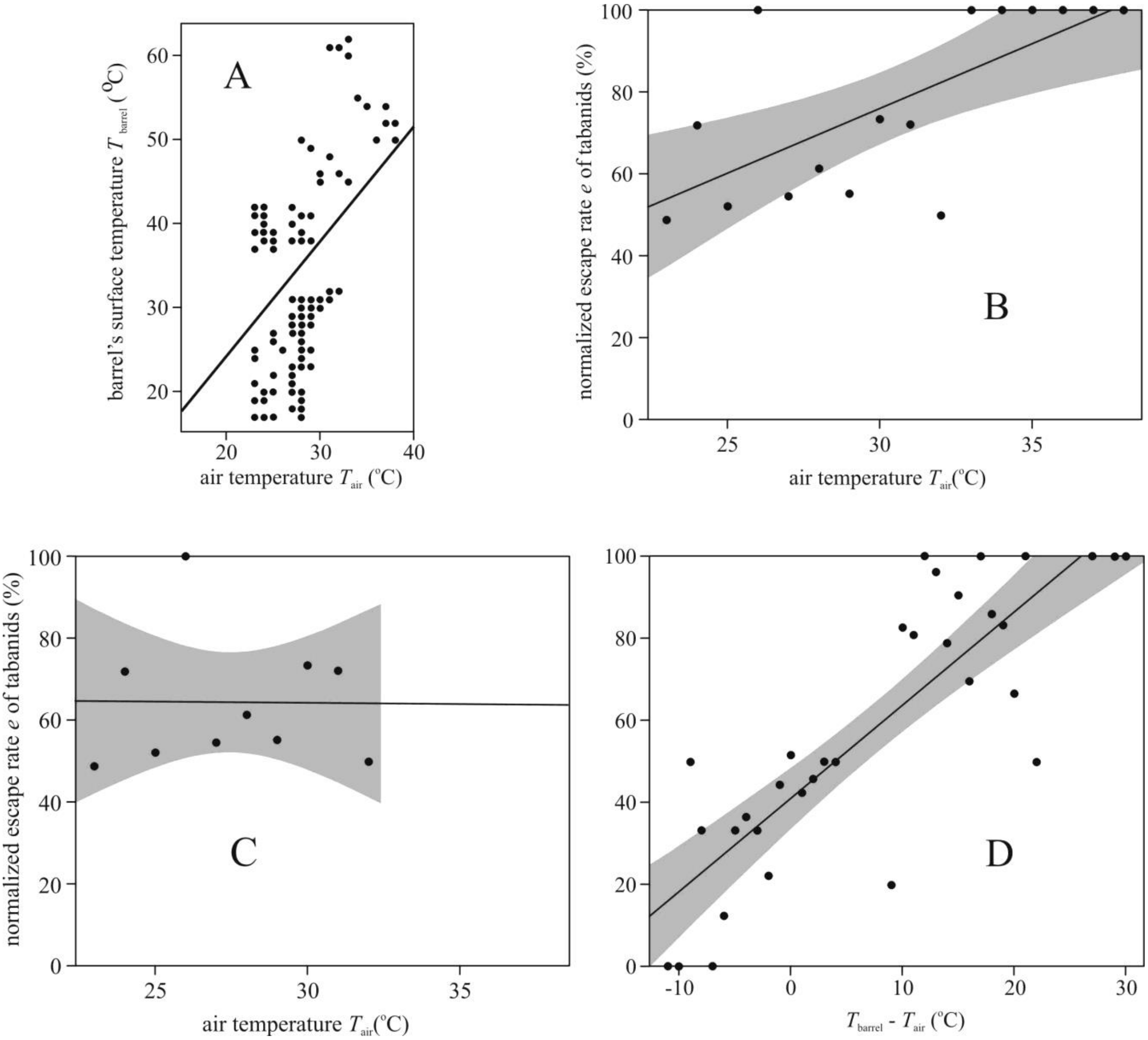

Figure 5. (A) The barrel's surface temperature T_{barrel} (°C) versus air temperature T_{air} (°C) in field experiments 1-5. A straight line indicates the linear fit to the $T_{barrel}(T_{air})$ data (black circles). (B) Normalized escape success *e* versus T_{air} (°C) for all five experiments. (C) As B, but only for $T_{air} \leq$ 32 °C. (D) Normalized escape success *e* versus T_{barrel} - T_{air} (°C) for all five experiments. In B-D continuous straight lines indicate the linear fit to the data (black circles), and dark grey bands around the fit show the 95 % confidence intervals.

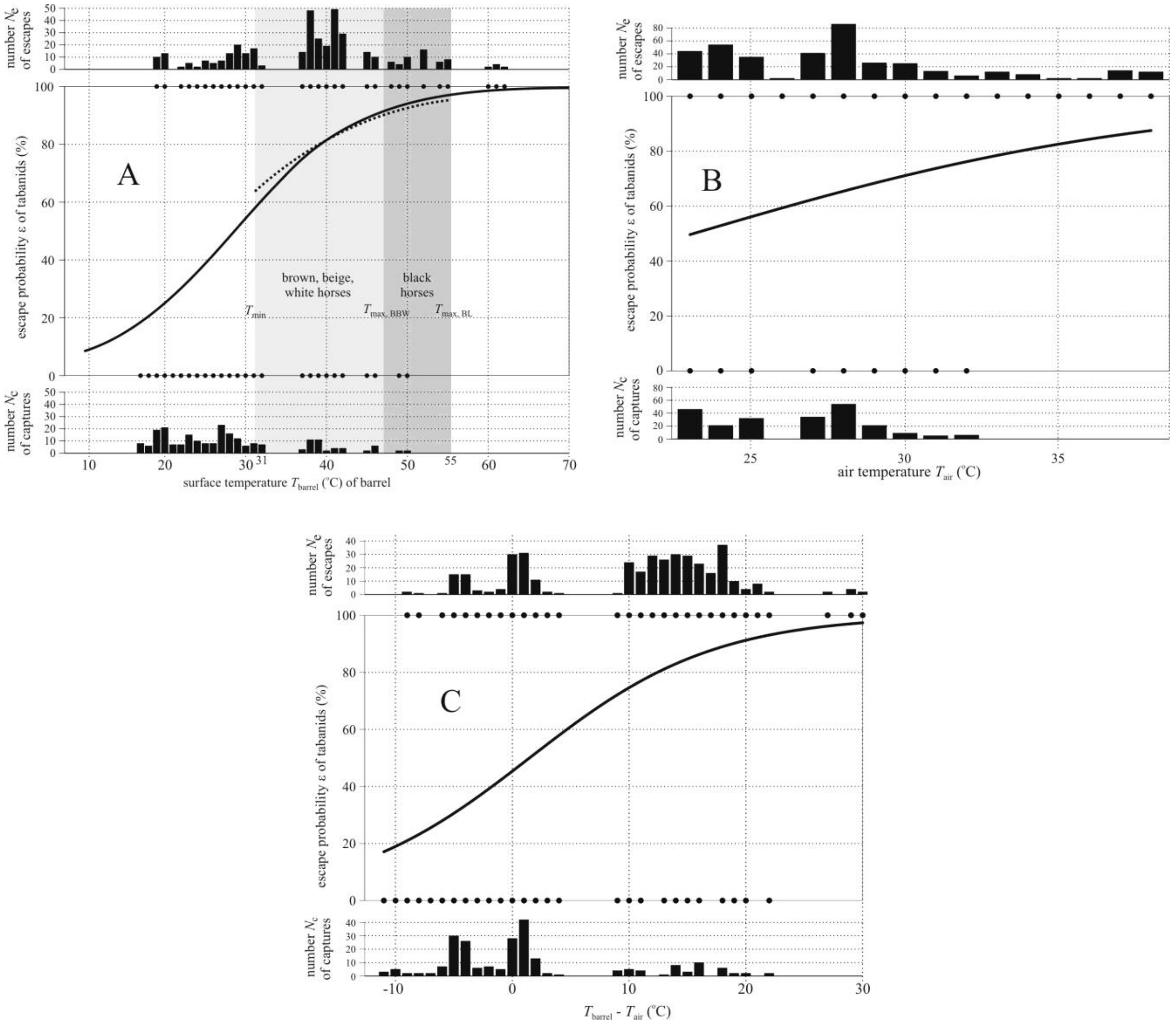

Figure 6. Results of logistic regressions. (A) *Top*: Number N_e of escaped horseflies versus the barrel's surface temperature T_{barrel}. *Middle*: Escape probability ε of horseflies, where the logistic curve is fitted to the dots showing the surface temperatures at which tabanids were escaped (ε = 100 %) and captured (ε = 0 %) within the 17 °C ≤ T_{barrel} ≤ 62 °C interval. A dotted curve illustrates the logistic fit to the data within the 31 °C ≤ T_{barrel} ≤ 55 °C interval. *Bottom*: Number N_c of captured horseflies versus T_{barrel}. The vertical light and medium grey columns denote the interval T_{min} = 31 °C ≤ T ≤ $T_{max,BBW}$ = 47 °C of the surface temperature of brown, beige and white (BBW) horses and the interval T_{min} = 31 °C ≤ T ≤ $T_{max,BL}$ = 55 °C of black (BL) horses measured by thermography (Fig. 2). (B) As A versus the air temperature T_{air}. (C) As A versus the difference T_{barrel} - T_{air}.

Point-by-Point Response to the Comments of Reviewer 1

We thank Referee 1 for her/his constructive comment. We have revised our paper on the basis of three reviews. All changes done with respect to the reviews of Referee 1, Referee 2 and Referee 3 are marked with blue, green and orange, respectively. Below one can read our Point-by-Point Response to the point of Referee 1.

Referee 1 wrote: I have one comment in chapter Introduction; last part of last sentence line 82 to 85 is not clear 'though the blood of shady or bright (cooler) hosts would also good for the development of tabanid eggs'. I would like to suggest delete this in the last sentence. Blood meal of warm blooded animals biting female horseflies use as source of energy for egg maturation, regardless if hosts are dark or bright colour or in shady place or in sunlit.

Answer: As suggested, this sentence was revised as follows:

The results of our field experiments presented here corroborated the mentioned prediction which explains why blood-seeking horseflies prefer sunlit dark (warmer) host animals.

Point-by-Point Response to the Comments of Reviewer 2

We thank Referee 2 for her/his constructive and valuable comments. We have revised our paper on the basis of three reviews. All changes done with respect to the reviews of Referee 1, Referee 2 and Referee 3 are marked with blue, green and orange, respectively. Below one can read our Point-by-Point Response to the points of Referee 2.

Referee 2 wrote: The article is about tabanid preferences to darker hosts. The authors tested the hypothesis that tabanids prefer dark sunlit warm hosts because high temperatures might increase wing muscle and nervous system performance so that tabanid females escape easily and quickly in case of host defensive reactions. First, the authors estimated surface temperatures of horses with different coat colour. Then, they compared tabanid escape success in the field using different barrels modelling horse surface temperatures. This study provides an interesting approach to explain why tabanids are attracted by warm hosts or targets since it is well-known that host attractivity is dependant on olfactive, visual and thermal cues. However, here are some minor concerns about the study:

- The authors analysed the results for all the tabanid population of the study area without considering differences between species. I understand that species identification is not feasible in the field, however the authors could have identified tabanid at the genus level. Tabanus, Atylotus and Haematopota have main characteristics that allow to discriminate them. There are differences between species in landing, daily activity and responses to environmental parameters that might influence their escape success. For example, Haematopota species might be more active in late afternoon when temperatures decrease. Maybe, the influence of temperatures on host preference for Haematopota sp. is lower than for Tabanus sp. or Atylotus sp. I think that this point should be discussed by the authors.

Answer: To the revised Discussion we added the following:

In this work we analysed the results of our field experiments for the whole tabanid population of the study area without considering differences between tabanid species/genus, because species/genus identification was not feasible in the field. However, there may be differences between species/genus in landing, daily activity and responses to environmental parameters that might influence their escape success. For example, Haematopota species might be more active in late afternoon when air temperature decreases. It is also unknown whether the influence of air temperature T_{air} on host preference for *Haematopota* sp. is lower than that for *Tabanus* or *Atylotus* sp. To test these hypotheses could be the task of further studies. What we know from our fields experiments is the following: (i) Apart from T_{air} (Supplementary Tables S6-S10) the weather situation (calm with no meteorological fronts) was the same during our field experiments. (ii) There was no correlation between the escape success e of landed horseflies and T_{air} < 33 °C (Fig. 5C). In this case the barrel temperature T_{barrel} determined the escape success e, rather than T_{air}.

Referee 2 wrote: - Field experiment setting: only the experiments 4 and 5 are well set to compare tabanid escape success between the two types of barrels modelling hosts with different temperatures in the same conditions. In experiments 1, 2 and 3, the two barrels were not tested simultaneously. They were used different days with different environmental conditions. However, factors other than temperatures (for example, wind) have an effect on tabanid activity and these other factors might also influenced escape success.

Answer: In the revised manuscript we wrote:

Field experiments

Field experiments 1-5 were performed on 1, 2, 3, 4 and 11 July 2019 on a Hungarian horse farm in Szokolya (47° 52' North, 19° 00' East), where horseflies were present. All five experimental days were windless, and only weak local winds blew in early afternoons. Meteorological fronts did not move through the site of experiments. Thus, apart from the air temperature (according to Supplementary Tables S6-S10, in experiment 1 the air temperatures were warmer than on the other four experimental days), the environmental conditions were practically the same.

Discussion

According to earlier field experiments [31, 32] in the same experimental site (a Hungarian horse farm in Szokolya) with the same tabanid species (*Tabanus tergestinus*, *T. bromius*, *T. bovinus*, *T. autumnalis*, *Atylotus fulvus*, *A. loewianus*, *A. rusticus*, *Haematopota italica*) as in the case of our present field experiments, the daily activity of different tabanid species and the effect of weather variables on their flight activity are slightly different. Herczeg *et al*. [32], for example, found the following: (i) Rainfall, air temperature, and sunshine were the three most important factors influencing the trapping number of tabanids. (ii) The effect of relative air humidity H on tabanids was indirect through the air temperature T: $H \approx 35$ % (corresponding to $T \approx 32$ °C) was optimal for tabanid trapping, and tabanids were not captured for $H \geq 80$ % (corresponding to $T \leq 18$ °C). (iii) A fast decrease in the air pressure enhanced the trapping number of horseflies. (iv) Wind velocities larger than 10 km/h reduced drastically the number of trapped tabanids.

In our field experiments 4 and 5 warm and cold sunlit barrels were used simultaneously, while in experiments 1-3 the cold and the warm barrels were tested separately (experiment 1: sunlit air-filled barrels, experiment 2: shady air-filled barrels, experiment 3: shady water-filled barrels). This was, however, not a problem, because apart from the air temperature (Supplementary Tables S6-S10) the environmental conditions (calm with no meteorological fronts) were practically the same on all experimental days. Thus, the slightly different environmental factors in our field experiments could have resulted in only small differences in the activity of a given tabanid species.

Referee 2 wrote: - Statistical analysis: the authors used a Khi^2 test to compare the escape/non-escape ratio versus the 50/50 ratio. This test did not allow to compare warm barrel versus cold barrel.

Answer: As Referee 2 wrote, χ^2 test was used to compare the escape/non-escape ratio versus the 50/50 ratio. But it is important to mention that χ^2 tests were performed to compare escape/non-escape numbers for a given barrel or barrel side (sunlit or shady). Consequently, the compared escape/non-escape numbers corresponded to the same barrel temperature and there was no comparison between data originating from different barrel temperatures. In other words, χ^2 test was used to detect whether a given barrel temperature had an effect on tabanid escape success. In the revised subsection 'Statistical analysis' we wrote:
For comparison of the numbers of escaped and captured tabanids landed on test surfaces of various temperatures, we applied χ^2 tests of homogeneity, where the escape versus non-escape ratio was tested against the predicted 50/50 ratio. These χ^2 tests were performed to compare escape/non-escape numbers for a given barrel or barrel side (sunlit or shady). Thus, the compared escape/non-escape numbers corresponded to the same barrel temperature and there was no comparison between data originating from different barrel temperatures. In other words, χ^2 test was used to detect whether a given barrel temperature had an effect on tabanid escape success.

Referee 2 wrote: - Discussion (lines 191-194): I suggest to develop this part and to cite some references about tabanid landings and host defensive reactions.

Answer: As suggested, we revised this part in the following way:

The blood meal from warm-blooded animals is used by biting female horseflies as an energy source for maturation of their eggs [25, 28, 29, 30]. For this purpose, the blood of any warm-blooded hosts is good, regardless if hosts are dark- or bright-coloured, or shady or sunlit. In spite of this, blood-seeking female tabanids prefer dark and sunlit hosts [1, 2, 3, 4, 5, 6, 7], and this is the reason why horsefly traps usually have black decoys and are most effective in sunshine [8, 9, 10, 11, 12, 13, 14, 15, 16]. Our main assumption was that blood-seeking tabanids prefer to land on sunlit dark hosts to keep their body warm, which aids their rapid escape when the host performs such typical antiparasite reactions as removing horseflies from their coats with tails brushing them off, by stamping and dislodging the flies, or by nibbling their skin, for instance [1, 30]. These fly-repelling reactions are dangerous for blood-sucking tabanids, therefore have to be avoided by a quick flying away.

Referee 2 wrote: I am not English-native speaker, but I think that the manuscript might be improved by an English speaker review.

Answer: During its revision, we tried to improve the English of our manuscript.

Point-by-Point Response to the Comments of Reviewer 3

We thank Referee 3 for her/his constructive and valuable comments. We have revised our paper on the basis of three reviews. All changes done with respect to the reviews of Referee 1, Referee 2 and Referee 3 are marked with blue, green and orange, respectively. Below one can read our Point-by-Point Response to the points of Referee 3.

Referee 3 wrote: General comments: The paper by Horváth et al. deals with the interesting hypothesis, demonstrated by the Authors, that horseflies, after landing, would prefer to walk around and feed on warmer hosts as this would allow them to be more reactive and ready to take off, escaping hosts defensive reactions. The paper is interesting for an entomologist working on horseflies, English language is very good, methods are sound and provided data strong, but there are some major concerns affecting the manuscript, to be evaluated before taking a decision regarding its publication on PLOS ONE. The first one regards the exceedingly excessive length of the manuscript that is really redundant and rambling. Whole sections should be reduced or totally deleted. Authors spent a lot of words both in materials and methods and results dealing with very obvious statements regarding horse temperatures, that should be considered not a for its own sake section, but just something functional to experiments on horseflies. As a consequence, all the sections dealing with horse temperatures should be reduced to few lines, also considering that results are very obvious: a dark horse is warmer than a white one, a horse in sunlight is warmer than a horse in the shadow, the belly is cooler than the back, and other similar things. This is very boring for the reader. Also figures, photos and graphs on this subject should be eliminated. The whole manuscript needs to be simpler and slenderer to make it really interesting.

Answer: As requested by Referee 3, in our revised manuscript we performed the suggested text shortenings. However, we kept the original Figures 1 and 2, because we as well as Referees 1 and 2 consider them as important.

Referee 3 wrote: But, there are also some logic and methodological problems, with contradictory statements and results. There is a methodological problem, which in theory could impair a big part of the results, needing an explanation by the Authors. I hereby detail the problems I revealed during the review, problems that have to be addressed by Authors with a very thorough revision, to make the manuscript deserving publication on PLOS ONE.

Answer: We addressed/corrected the logical and methodological problems, as well as the contradictory statements criticized by Referee 3.

Referee 3 wrote: Specific Comments

1. Manuscript Length: a) Lines 93-110: reduce to 3-5 lines at maximum, as they are very obvious results, which should be only functional to the experiments on horseflies.

Answer: As suggested, the following two paragraphs

Figure 1 and Supplementary Figs. S1-S4 show the photographs and thermograms of sunlit black, brown, beige and white horses as typical examples. It is clearly seen that the temperature of the sunlit back of horses decreases in the following colour order: black, brown, beige and white, because darker sunlit surfaces absorb more sunlight resulting in elevated temperatures [18, 19]. The mean temperature of the bellies exhibits less variability, because the incident angle of sunlight was almost parallel with the belly surface.

In the case of a given horse, the surface temperature changed spatially due to the continuous curvature of the body surface receiving sunlight at various angles. Figure 2 shows the minimum (T_{min}), maximum (T_{max}), average (<T>) and standard deviation (ΔT) of the surface temperature of the back and belly of black, brown, beige and white horses measured by thermography under different illuminations (Supplementary Figs. S1-S4, Supplementary Tables S1-S4). Considering the average temperature <T>, the shady side of sunlit horses was always cooler than the sunlit side. Under cloudy skies, <T> of a given horse was lower than in sunlit situations, as expected. In sunshine, the backs of black horses were the warmest, brown and beige backs were cooler, and the backs of white horses were the coldest. In sunshine the belly of a given horse was always cooler than its back. The observed minimum and maximum temperatures of horses were the following: black: 30.9-54.6 ^oC, brown: 31.2-44.6 ^oC, beige: 32.6-46.2 ^oC, white: 31.0-46.6 ^oC. The range T_{max} - T_{min} and the standard deviation ΔT increased with increasing mean temperature <T>.

were shortened as follows:

As expected, the surface temperature T of the sunlit back of horses decreased in the colour order black > brown > beige > white, and the mean temperature <T> of the bellies had smaller standard deviation ΔT than the backs (Fig. 1, Supplementary Figs. S1-S4). The minimum and maximum surface temperatures of horses were: black: 30.9-54.6 ^oC, brown: 31.2-44.6 ^oC, beige: 32.6-46.2 ^oC, white: 31.0-46.6 ^oC. The range T_{max} - T_{min} and ΔT increased with increasing <T> (Fig. 2, Supplementary Tables S1-S4).

Referee 3 wrote: Moreover, remove figures and graphs only dealing with horse temperatures.

Answer: Figure 1 is important, because it shows typical thermograms of the black, brown, beige and white horses studied by us together with the back and belly areas where the surface temperature was averaged.

Figure 2 is also relevant, since it displays the minimum, maximum, average and standard deviation of the surface temperature of the back and belly of black, brown, beige and white horses measured by us with thermography under different illumination conditions.

Since we consider of importance Figures 1 and 2, we did not remove/delete them. Note that neither Referee 1, nor Referee 2 suggested the removal of these figures. All six figures of our paper are relevant for the informative presentation of our results.

Referee 3 wrote: b) Lines 145-149: these lines should be deleted, as results of this analysis are clear enough also without this heavy introduction.

Answer: The criticized sentences

Figure 5A displays the barrel's surface temperature T_{barrel} versus air temperature T_{air} in field experiments 1-5. These two temperatures have a positive correlation, because at lower air temperatures the sunlit/shady air-filled (warm) barrels could not warm up as much as at higher air temperatures. In experiment 1 (when the weather was very warm) we used only air-filled sunlit warm barrels. Therefore, in this case low T_{barrel}-values were missing at high air temperatures T_{air}.

were shortened in the following way:

We can see in Fig. 5A that the barrel's surface temperature T_{barrel} and the air temperature T_{air} correlate positively.

Referee 3 wrote: c) Lines 197-201: delete these lines. Not necessary and only making the discussion heavier.

Answer: As suggested, these sentences were deleted:

As far as we know, the time period for flies to change the temperature of their wing muscles has never been measured. This would be a very difficult task, because it would be problematic to measure the wing muscle temperature, and the speed of its warming up depends on the local temperature of the air and the thorax containing the muscles, the measurements of which would also be difficult.

Referee 3 wrote: d) Lines 209-218: delete these lines. They are an unnecessary and really unexplainable repetition of things already said elsewhere in the manuscript. They are Introduction, Materials and Methods, not a Discussion at all.

Answer: As suggested, the following paragraph and its references were deleted:

We studied experimentally the success rate of escape reactions of tabanid flies landed on black targets as a function of the surface temperature. Our aim was to test the prediction of Horváth *et al*. [17]. In order to avoid the many difficulties with freely moving horses, we used artificial targets mimicking the surface temperatures of darker (black, brown) and brighter (beige, white) ungulates, but of the same optical attractiveness. The latter condition is essential, because it is widely known that bright surfaces repel tabanids [5, 29, 30, 31, 32, 33, 34, 35]. Optically uniform shiny black barrels proved as an optimal choice: the surface temperature of these barrels could be changed in a wide range with changing the filling (air or cold water) and illumination (sunlit or shady), but the visual attractiveness to tabanids (radiance, colour, polarization) remained the same. Very similar methodology was used successfully in earlier field experiments [17].

29. Bracken GK, Hanec W, Thorsteinson AJ. The orientation behaviour of horse flies and deer flies (Tabanidae: Diptera). II. The role of some visual factors in the attractiveness of decoy silhouettes. Canadian Journal of Zoology 1962; 40: 685-695.
30. Granger CA. Trap design and color as factors in trapping the salt marsh greenhead fly. Journal of Economical Entomology 1970; 63: 1670-1673.
31. Browne SM, Bennett GF. Colour and shape as mediators of host-seeking responses of simuliids and tabanids (Diptera) in the Tantramar marshes, New Brunswick, Canada. Journal of Medical Entomology 1980; 17: 58-62.
32. Allan SA, Stoffolano JG. The effects of hue and intensity on visual attraction of adult Tabanus nigrovittatus (Diptera: Tabanidae). Journal of Medical Entomology 1986; 23: 83-91.
33. Sasaki H. Comparison of capturing tabanid flies (Diptera: Tabanidae) by five different color traps in the fields. Applied Entomology and Zoology 2001; 36: 515-519.
34. Krcmar S. Comparison of the efficiency of the olfactory and visual traps in the collection of horseflies (Diptera: Tabanidae). Entomologia Generalis 2013; 34: 261-267.
35. Horváth G, Blahó M, Egri Á, Lerner A. Applying polarization-based traps to insect control. In: G. Horváth (ed.) Polarized Light and Polarization Vision in Animal Sciences. 2014; Springer: Heidelberg, pp. 561-584.

Referee 3 wrote: e) Lines 265-278: the same as for point a). Reduce to 3 lines. This section is not for its own sake, but only functional to experiments on tabanids. Moreover, most part of relative results are very obvious. Again, shorten all the sections regarding horse temperatures and remove graphs, pics. They all make the paper very strenuous to read.

Answer: As suggested, the following subsection

Thermography of horses: On 6 July 2019 the distribution of the surface temperature (thermogram) of black, brown, beige and white horses was measured with an infrared camera (VarioCAM®, Jenoptik Laser Optik Systeme GmbH, Jena, Germany, nominal precision of ±1.5 °C) under sunny and cloudy (when the sun was behind clouds) conditions. We recorded several infrared snapshots of a given horse and selected typical thermograms where the optical axis of the thermocamera was nearly perpendicular to the long axis of the horse. To determine the average surface temperature of horses over their back and belly, we used a custom-developed software. The validation and calibration of our thermocamera with a contact thermometer (GAO Digital Multitester EM392B 06554H, EverFlourish Europe Gmbh., Friedrichsthal, Germany, nominal precision of ±1 °C) was described in detail in the Supporting Material of [17]. On each thermogram an extended area was selected on the back and belly of horses. Within these areas the mean temperature $<T>$, its standard deviation ΔT, minimum $T_{\min}$ and maximum $T_{\max}$ were determined with a custom-developed software in order to compare characteristic surface temperatures of 2 black, 2 brown, 2 beige and 2 white horses under sunlit and shady conditions on 46 thermograms.

was shortened in the following way:

Thermography of horses: On a warm day (6 July 2019) 46 thermograms of 2 black, 2 brown, 2 beige and 2 white horses were measured with an infrared camera (VarioCAM®, Jenoptik Laser Optik Systeme GmbH, Jena, Germany, nominal precision of ±1.5 °C) under sunny and cloudy conditions. The validation and calibration of this thermocamera with a contact thermometer (GAO Digital Multitester EM392B 06554H, EverFlourish Europe Gmbh., Friedrichsthal, Germany, nominal precision of ±1 °C) were described in the Supporting Material of [20].

Referee 3 wrote: f) Lines 290-292: not necessary. Delete;
g) Lines 295-296: not necessary. Delete.

Answer: As suggested, the following sentences were deleted:

We did not use barrels filled with warm/hot water, because the continuous production of warm/hot water throughout the day was technically impossible in the field.

The barrels were closed by black caps which hindered the outflow of air/water or the loss by evaporation.

Referee 3 wrote: h) Lines 326-329: shorten this explanation. It was not possible for technical problems. Most part of readers will be entomologists!!!.

Answer: The original sentence

For this purpose, thermography could not have been used, because (i) the thermocamera needed about two minutes for self-calibration after each switch on, whilst the next tabanids could land on the barrels,

and (ii) on the recorded thermograms it would have been impossible to localize the exact landing sites of tabanids.

was shortened as suggested:

For this purpose, thermography was not possible to use because of technical problems.

Referee 3 wrote: i) Lines 342-345: Delete these lines. Authors just said that from an optical point of view the barrels were the same, this is enough.

Answer: As suggested, the following sentence was deleted.

Only tiny water droplets condensed from air humidity on the surface of the cold barrels could have resulted in some differences in reflection-polarization, but such condensation was never observed in our field experiments.

Referee 3 wrote: 2. Methodological problem: What reported at lines 89-92 is quite strange and needs to be explained by the Authors. In fact, while warm and cold barrels ranges of temperatures don't overlap, warm and 'cold horses' are highly overlapping. Moreover, while warm barrels are, for the most part, in the range of temperatures of 'warm horses', cold barrels temperatures are lower than those of 'colder horses', totally out of the range of temperature of 'cold horses'. This is very strange. Standing this situation, it seems that authors used cold barrels too cold, don't reflecting a real situation and hence possibly producing wrong results. This is really very relevant. The use of so cold barrels could invalidate the whole study. Authors should explain this choice and why, in their opinion, this is not affecting the validity of the work.

Answer: During our field experiments we could not keep the surface temperature of barrels within a desired range. That is why we chose the solution to use air-filled barrels to imitate warm hosts (which represents e.g. sunlit dark horses) and cold water-filled barrels with ice-packs to imitate cooler hosts. The temperature of barrels changed with time and was altered by the daily air temperature, so we regularly measured their surface temperature. Because of the experimental setup, the range of the barrels’ surface temperature was wider than the observed surface temperature range of the studied horses. During the linear and logistic regressions we used the barrel temperature as the independent variable and not the cold/warm barrel categorical variable. That is why cold/warm barrel categories were irrelevant at the regressions. Furthermore, we also applied the linear and logistic regressions as a funciton of the barrel temperature using the data within the 31 °C ≤ T ≤ 55 °C interval. This temperature range is what we have measured on living horses. The results of the linear and logistic regressions for the 31 °C ≤ T ≤ 55 °C interval and the wider 17 °C ≤ T ≤ 62 °C range were similar: the escape rate *e* increased with increasing T_{barrel}. On the basis of these, the criticized sentences were moved from the Results to the revised Discussion in which we wrote the following:

During the field experiments all barrels were optically uniformly attractive to host-seeking horseflies. Our air-filled sunlit warm barrels (37-62 ^{o}C) thermally imitated sunlit black horses (31-55 ^{o}C), while our water-filled sunlit cold barrels (19-29 ^{o}C) were cooler than sunlit brown, beige and white horses (31-47 ^{o}C). Although the measured low escape successes in the case of our water-filled cold barrels were associated with temperatures that were lower than those on the studied horse bodies, the positive correlation between escape success *e* and surface temperature *T* (i.e. increasing *e* with increasing *T*) is also evident in the interval T_{min} = 31 ^{o}C ≤ T ≤ $T_{max,BL}$ = 55 ^{o}C as clearly shown by the dotted curves of Figs. 4C and 6A. Thus, the results of our field experiments show that the escape success of tabanids

depends on the host's surface temperature *T*: the higher the *T*, the larger the escape success of horseflies. The average surface temperature of the studied sunlit black horses was between 48 and 55 °C. Almost all tabanids managed to escape in this temperature range.

Referee 3 wrote: 3. Contradictory Statements and Needed Explanations: a) Lines 66-68: if it is true, as Authors say in another part of the manuscript, that tabanids are not able to feel temperature of an object before landing on it, what stated in these lines is in contradiction with what Authors try to demonstrate in the paper. Black decoys used for catching horseflies would work not for their temperature, but for their colour.

Answer: There is here no any contradiction! On the one hand, to the revised Introduction we added the following:

The most effective tabanid traps use shiny black decoys [8, 9, 10, 11, 12, 13, 14, 15]. The so-called H-traps – composed of a bright tent with a shiny black sphere suspended below it – placed in sunny sites capture significantly more female tabanids than at shady sites [16]. The reason for this is that sunlit shiny dark targets reflect light at the Brewster's angle with higher degrees of linear polarization *d* than shady ones [17, 18], and host-seeking female tabanids prefer high *d*-values independent of the direction of polarization [19]. Thus, shiny black decoys used to catch horseflies work for their colour and reflected degree of polarization, rather than for their temperature.

On the other hand, to the revised Discussion we added the following:

Since the thermoreceptors of tabanids (as usually in Diptera) are in their legs, antennae and mouthpart [28, 29, 30], they cannot sense remotely (e.g. by infrareceptors) the temperature of a target. They can sense precisely the surface temperature of a substrate/host only after a physical contact (landing). However, on the basis of the leg/antenna/mouth-sensed temperature of the boundary layer around a target, tabanids can decide whether the target's surface is or is not warm enough for alighting [20].

Referee 3 wrote: b) Lines 129-144: the whole paragraph is very mixed up, difficult to read and understand. Furthermore, Authors don't explain some of the findings. Why there is a drop of escaped tabanids at very high temperatures? Zero at 62 °C? Why fewer tabanids landed on cold barrels if they feel temperature only after landing? Why the specification at lines 139-140? All these things are quite obscure for the reader. Authors should greatly simplify this section, explain these points and report only the relevant results.

Answer: Note that the missed explanations were partly involved in the original version of this criticized paragraph:

Referee 3 asked: Why there is a drop of escaped tabanids at very high temperatures? Zero at 62 °C? Why the specification at lines 139-140 (Note again that the waiting time until a catch attempt was 10 seconds after a tabanid landed on a barrel.)?

The answer is: At higher surface temperatures more tabanids landed on the barrels, but because of the hot surface many of them flew away before the capturing attempt was performed in 10 seconds after landing (see Materials and Methods and Discussion). Therefore, the landing events shorter than 10 seconds were not registered, thus N_e dropped with increasing *T*.

Referee 3 asked: Why fewer tabanids landed on cold barrels if they feel temperature only after landing?

The answer is: The explanation of the drop of N_e at lower surface temperatures is that (i) less tabanids landed on colder surfaces, because tabanids flying through the cold boundary layer around the cold barrels could sense the lower surface temperature by their thermoreceptors (in the legs, antennae, mouthpart) and decided less frequently to alight, as Horváth *et al*. [20] showed, and (ii) the escape rate is lower at colder surfaces (see Fig. 4C).

As requested by Referee 3, this paragraph was revised in the following way:

According to Fig. 4A, the number N_e of escaped tabanids landed on barrels has a maximum at around 41 °C, drops to zero at 17 °C and decreases almost to zero at 62 °C. The explanation of the drop of N_e at lower surface temperatures is that (i) less tabanids landed on colder surfaces, because tabanids flying through the cold boundary layer around the cold barrels could sense the lower surface temperature by their thermoreceptors (in the legs, antennae, mouthpart) and decided less frequently to alight, as Horváth *et al*. [20] showed, and (ii) the escape rate is lower at colder surfaces (see Fig. 4C). At higher surface temperatures more tabanids landed on the barrels, but because of the hot surface many of them flew away before the capturing attempt was performed in 10 seconds after landing (see Materials and Methods and Discussion). Therefore, the landing events shorter than 10 seconds were not registered, thus N_e droped with increasing T. In Fig. 4B, the number N_c of captured tabanids landed on barrels exhibits a clear decreasing trend with increasing T_{barrel} for both temperature intervals of 17 °C ≤ T ≤ 62 °C and T_{min} = 31 °C ≤ T ≤ $T_{max,BL}$ = 55 °C. In Fig. 4C the increasing tendency of the normalized escape succes $e = N_e/(N_e + N_c)$ with increasing barrel's surface temperature T_{barrel} is clear. Figure 4C also illustrates that the systematic increase of the escape success e is also present in the temperature range T_{min} = 31 °C ≤ T ≤ $T_{max,BL}$ = 55 °C that is typical for the surface of horses.

Referee 3 wrote: c) Line 294: Why 10 seconds? In Materials and Methods Authors should explain this choice.

Answer: In the revised manuscript we wrote:

Field experiments

The waiting time until a catch attempt was 10 seconds (measured with a stopwatch) after a tabanid landed on a barrel (the choice of this optimal 10-second value is explained in the Discussion).

Results

Note that the waiting time until a catch attempt was 10 seconds after a tabanid landed on a barrel (see Materials and Methods and Discussion).

Discussion

In our field experiments the investigator waited 10 seconds before he tried to capture a tabanid landed on a barrel. This 10-second period turned out to be optimal: it was neither too short, nor too long. Within a period shorter than 10 s the flight muscles of landed tabanids could not warm up or cool down to the surface temperature of barrels. On the other hand, when a landed tabanid recognizes that the barrel is not a host animal, it flies away after a certain period. If the waiting period of counting of landed tabanids were too long, then many landing events would not be counted/registered. The air temperature did not correlate with the escape rate (Fig. 5C), therefore 10 s was long enough to warm the wing muscles of flies landed on the barrel. On the other hand, if a tabanid lands on a sunlit dark

(warm) surface, then it does not cool down, contrary to a bright (colder) surface where its wing muscles can cool down. It is pertinent to suppose that during flight the wing muscles are appropriately warm. Furthermore, according to Heinrich [33], many Dipteran species (including true flies and also tsetse flies) can be heterothermic, or generate certain amount of heat which can then be used to improve their performance. However, as far as we know, practically nothing is known about the thermoregulation in horseflies. Thus, without knowing the horsefly thermal physiology, we restricted our study to the correlation between (air/barrel) temperature and escape success with a waiting period of 10 seconds before the experimenter tried to capture a tabanid landed on a barrel.

Referee 3 wrote: d) Lines 331-333: it is not clear how Authors prevented the simultaneous landing of two or more tabanids on barrels. They give it for granted, but it is not.

Answer: We did not need to prevent the simultaneous landing of two or more tabanids on the barrels, because in the barrels' vicinity there were no so many flying tabanids. In the revised manuscript we wrote:

In the vicinity of the barrels there were no many flying tabanids. Threfore, only one tabanid landed on one of the two (warm or cold) barrels at a given time, thus the experimenter did not have to split his attention between two or more landed tabanids.

Referee 3 wrote: At Line 62, 'spp.' not in italics.

Answer: Italic 'ssp.' was corrected to normal 'spp.'.

Referee 3 wrote: At line 84 'would also good for' should be changed in 'would also be good for'.

Answer: As suggested by Referee 1, this sentence was revised as follows:

The results of our field experiments presented here corroborated the mentioned prediction which explains why blood-seeking horseflies prefer sunlit dark (warmer) host animals.

Referee 3 wrote: In conclusion, the paper by Horváth et al. could deserve publication on PLOS ONE only after major changes regarding its length and some logic inconsistency that Authors should modify or better explain.

Answer: The changes regarding length and some logic inconsistency suggested by Referee 3 were done in our revised manuscript.
