# Supplementary material for: Why do biting horseflies prefer warmer hosts? tabanids can escape easier from warmer targets
Source: PLoS One. 2020 May 13;15(5):e0233038. doi: 10.1371/journal.pone.0233038 (PMC7219777; doi:10.1371/journal.pone.0233038)
Supplement: Attachment — Submitted filename: +TabanidThermalTrapping_PLoS-One-response.doc [file pone.0233038.s019.doc]

**Point-by-Point Response to the Comments of Reviewer 1**

We thank Referee 1 for her/his constructive comment. We have revised our paper on the basis of three reviews. All changes done with respect to the reviews of Referee 1, Referee 2 and Referee 3 are marked with blue, green and orange, respectively. Below one can read our Point-by-Point Response to the point of Referee 1.

**Referee 1 wrote**: I have one comment in chapter Introduction; last part of last sentence line 82 to 85 is not clear 'though the blood of shady or bright (cooler) hosts would also good for the development of tabanid eggs'. I would like to suggest delete this in the last sentence. Blood meal of warm blooded animals biting female horseflies use as source of energy for egg maturation, regardless if hosts are dark or bright colour or in shady place or in sunlit.

**Answer**: As suggested, this sentence was revised as follows:

The results of our field experiments presented here corroborated the mentioned prediction which explains why blood-seeking horseflies prefer sunlit dark (warmer) host animals.

**Point-by-Point Response to the Comments of Reviewer 2**

We thank Referee 2 for her/his constructive and valuable comments. We have revised our paper on the basis of three reviews. All changes done with respect to the reviews of Referee 1, Referee 2 and Referee 3 are marked with blue, green and orange, respectively. Below one can read our Point-by-Point Response to the points of Referee 2.

**Referee 2 wrote**: The article is about tabanid preferences to darker hosts. The authors tested the hypothesis that tabanids prefer dark sunlit warm hosts because high temperatures might increase wing muscle and nervous system performance so that tabanid females escape easily and quickly in case of host defensive reactions. First, the authors estimated surface temperatures of horses with different coat colour. Then, they compared tabanid escape success in the field using different barrels modelling horse surface temperatures. This study provides an interesting approach to explain why tabanids are attracted by warm hosts or targets since it is well-known that host attractivity is dependant on olfactive, visual and thermal cues. However, here are some minor concerns about the study:

- The authors analysed the results for all the tabanid population of the study area without considering differences between species. I understand that species identification is not feasible in the field, however the authors could have identified tabanid at the genus level. Tabanus, Atylotus and Haematopota have main characteristics that allow to discriminate them. There are differences between species in landing, daily activity and responses to environmental parameters that might influence their escape success. For example, Haematopota species might be more active in late afternoon when temperatures decrease. Maybe, the influence of temperatures on host preference for Haematopota sp. is lower than for Tabanus sp. or Atylotus sp. I think that this point should be discussed by the authors.

**Answer**: To the revised Discussion we added the following:

In this work we analysed the results of our field experiments for the whole tabanid population of the study area without considering differences between tabanid species/genus, because species/genus identification was not feasible in the field. However, there may be differences between species/genus in landing, daily activity and responses to environmental parameters that might influence their escape success. For example, Haematopota species might be more active in late afternoon when air temperature decreases. It is also unknown whether the influence of air temperature *T*_air_ on host preference for *Haematopota* sp. is lower than that for *Tabanus* or *Atylotus* sp. To test these hypotheses could be the task of further studies. What we know from our fields experiments is the following: (i) Apart from *T*_air_ (Supplementary Tables S6-S10) the weather situation (calm with no meteorological fronts) was the same during our field experiments. (ii) There was no correlation between the escape success *e* of landed horseflies and *T*_air_ < 33 °C (Fig. 5C). In this case the barrel temperature *T*_barrel_ determined the escape success *e*, rather than *T*_air_.

**Referee 2 wrote**: - Field experiment setting: only the experiments 4 and 5 are well set to compare tabanid escape success between the two types of barrels modelling hosts with different temperatures in the same conditions. In experiments 1, 2 and 3, the two barrels were not tested simultaneously. They were used different days with different environmental conditions. However, factors other than temperatures (for example, wind) have an effect on tabanid activity and these other factors might also influenced escape success.

**Answer**: In the revised manuscript we wrote:

Field experiments

Field experiments 1-5 were performed on 1, 2, 3, 4 and 11 July 2019 on a Hungarian horse farm in Szokolya (47^o^ 52' North, 19^o^ 00' East), where horseflies were present. All five experimental days were windless, and only weak local winds blew in early afternoons. Meteorological fronts did not move through the site of experiments. Thus, apart from the air temperature (according to Supplementary Tables S6-S10, in experiment 1 the air temperatures were warmer than on the other four experimental days), the environmental conditions were practically the same.

Discussion

According to earlier field experiments [31, 32] in the same experimental site (a Hungarian horse farm in Szokolya) with the same tabanid species (*Tabanus tergestinus*, *T. bromius*, *T. bovinus*, *T. autumnalis*, *Atylotus fulvus*, *A. loewianus*, *A. rusticus*, *Haematopota italica*) as in the case of our present field experiments, the daily activity of different tabanid species and the effect of weather variables on their flight activity are slightly different. Herczeg *et al*. [32], for example, found the following: (i) Rainfall, air temperature, and sunshine were the three most important factors influencing the trapping number of tabanids. (ii) The effect of relative air humidity *H* on tabanids was indirect through the air temperature *T*: *H* ≈ 35 % (corresponding to *T* ≈ 32 °C) was optimal for tabanid trapping, and tabanids were not captured for *H* ≥ 80 % (corresponding to *T* ≤ 18 °C). (iii) A fast decrease in the air pressure enhanced the trapping number of horseflies. (iv) Wind velocities larger than 10 km/h reduced drastically the number of trapped tabanids.

In our field experiments 4 and 5 warm and cold sunlit barrels were used simultaneously, while in experiments 1-3 the cold and the warm barrels were tested separately (experiment 1: sunlit air-filled barrels, experiment 2: shady air-filled barrels, experiment 3: shady water-filled barrels). This was, however, not a problem, because apart from the air temperature (Supplementary Tables S6-S10) the environmental conditions (calm with no meteorological fronts) were practically the same on all experimental days. Thus, the slightly different environmental factors in our field experiments could have resulted in only small differences in the activity of a given tabanid species.

**Referee 2 wrote**: - Statistical analysis: the authors used a Khi^2^ test to compare the escape/non-escape ratio versus the 50/50 ratio. This test did not allow to compare warm barrel versus cold barrel.

**Answer**: As Referee 2 wrote, χ^2^ test was used to compare the escape/non-escape ratio versus the 50/50 ratio. But it is important to mention that χ^2^ tests were performed to compare escape/non-escape numbers for a given barrel or barrel side (sunlit or shady). Consequently, the compared escape/non-escape numbers corresponded to the same barrel temperature and there was no comparison between data originating from different barrel temperatures. In other words, χ^2^ test was used to detect whether a given barrel temperature had an effect on tabanid escape success. In the revised subsection ‘Statistical analysis’ we wrote:

For comparison of the numbers of escaped and captured tabanids landed on test surfaces of various temperatures, we applied χ^2^ tests of homogeneity, where the escape versus non-escape ratio was tested against the predicted 50/50 ratio. These χ^2^ tests were performed to compare escape/non-escape numbers for a given barrel or barrel side (sunlit or shady). Thus, the compared escape/non-escape numbers corresponded to the same barrel temperature and there was no comparison between data originating from different barrel temperatures. In other words, χ^2^ test was used to detect whether a given barrel temperature had an effect on tabanid escape success.

**Referee 2 wrote**: - Discussion (lines 191-194): I suggest to develop this part and to cite some references about tabanid landings and host defensive reactions.

**Answer**: As suggested, we revised this part in the following way:

The blood meal from warm-blooded animals is used by biting female horseflies as an energy source for maturation of their eggs [25, 28, 29, 30]. For this purpose, the blood of any warm-blooded hosts is good, regardless if hosts are dark- or bright-coloured, or shady or sunlit. In spite of this, blood-seeking female tabanids prefer dark and sunlit hosts [1, 2, 3, 4, 5, 6, 7], and this is the reason why horsefly traps usually have black decoys and are most effective in sunshine [8, 9, 10, 11, 12, 13, 14, 15, 16]. Our main assumption was that blood-seeking tabanids prefer to land on sunlit dark hosts to keep their body warm, which aids their rapid escape when the host performs such typical antiparasite reactions as removing horseflies from their coats with tails brushing them off, by stamping and dislodging the flies, or by nibbling their skin, for instance [1, 30]. These fly-repelling reactions are dangerous for blood-sucking tabanids, therefore have to be avoided by a quick flying away.

**Referee 2 wrote**: I am not English-native speaker, but I think that the manuscript might be improved by an English speaker review.

**Answer**: During its revision, we tried to improve the English of our manuscript.

**Point-by-Point Response to the Comments of Reviewer 3**

We thank Referee 3 for her/his constructive and valuable comments. We have revised our paper on the basis of three reviews. All changes done with respect to the reviews of Referee 1, Referee 2 and Referee 3 are marked with blue, green and orange, respectively. Below one can read our Point-by-Point Response to the points of Referee 3.

**Referee 3 wrote**: General comments: The paper by Horváth et al. deals with the interesting hypothesis, demonstrated by the Authors, that horseflies, after landing, would prefer to walk around and feed on warmer hosts as this would allow them to be more reactive and ready to take off, escaping hosts defensive reactions. The paper is interesting for an entomologist working on horseflies, English language is very good, methods are sound and provided data strong, but there are some major concerns affecting the manuscript, to be evaluated before taking a decision regarding its publication on PLOS ONE. The first one regards the exceedingly excessive length of the manuscript that is really redundant and rambling. Whole sections should be reduced or totally deleted. Authors spent a lot of words both in materials and methods and results dealing with very obvious statements regarding horse temperatures, that should be considered not a for its own sake section, but just something functional to experiments on horseflies. As a consequence, all the sections dealing with horse temperatures should be reduced to few lines, also considering that results are very obvious: a dark horse is warmer than a white one, a horse in sunlight is warmer than a horse in the shadow, the belly is cooler than the back, and other similar things. This is very boring for the reader. Also figures, photos and graphs on this subject should be eliminated. The whole manuscript needs to be simpler and slenderer to make it really interesting.

**Answer**: As requested by Referee 3, in our revised manuscript we performed the suggested text shortenings. However, we kept the original Figures 1 and 2, because we as well as Referees 1 and 2 consider them as important.

**Referee 3 wrote**: But, there are also some logic and methodological problems, with contradictory statements and results. There is a methodological problem, which in theory could impair a big part of the results, needing an explanation by the Authors. I hereby detail the problems I revealed during the review, problems that have to be addressed by Authors with a very thorough revision, to make the manuscript deserving publication on PLOS ONE.

**Answer**: We addressed/corrected the logical and methodological problems, as well as the contradictory statements criticized by Referee 3.

**Referee 3 wrote**: Specific Comments

1. Manuscript Length: a) Lines 93-110: reduce to 3-5 lines at maximum, as they are very obvious results, which should be only functional to the experiments on horseflies.

**Answer**: As suggested, the following two paragraphs

Figure 1 and Supplementary Figs. S1-S4 show the photographs and thermograms of sunlit black, brown, beige and white horses as typical examples. It is clearly seen that the temperature of the sunlit back of horses decreases in the following colour order: black, brown, beige and white, because darker sunlit surfaces absorb more sunlight resulting in elevated temperatures [18, 19]. The mean temperature of the bellies exhibits less variability, because the incident angle of sunlight was almost parallel with the belly surface.

In the case of a given horse, the surface temperature changed spatially due to the continuous curvature of the body surface receiving sunlight at various angles. Figure 2 shows the minimum (*T*_min_), maximum (*T*_max_), average (<*T*>) and standard deviation (Δ*T*) of the surface temperature of the back and belly of black, brown, beige and white horses measured by thermography under different illuminations (Supplementary Figs. S1-S4, Supplementary Tables S1-S4). Considering the average temperature <*T*>, the shady side of sunlit horses was always cooler than the sunlit side. Under cloudy skies, <*T*> of a given horse was lower than in sunlit situations, as expected. In sunshine, the backs of black horses were the warmest, brown and beige backs were cooler, and the backs of white horses were the coldest. In sunshine the belly of a given horse was always cooler than its back. The observed minimum and maximum temperatures of horses were the following: black: 30.9-54.6 ^o^C, brown: 31.2-44.6 ^o^C, beige: 32.6-46.2 ^o^C, white: 31.0-46.6 ^o^C. The range *T*_max_ - *T*_min_ and the standard deviation Δ*T* increased with increasing mean temperature <*T*>.

were shortened as follows:

As expected, the surface temperature *T* of the sunlit back of horses decreased in the colour order black > brown > beige > white, and the mean temperature <*T*> of the bellies had smaller standard deviation Δ*T* than the backs (Fig. 1, Supplementary Figs. S1-S4). The minimum and maximum surface temperatures of horses were: black: 30.9-54.6 ^o^C, brown: 31.2-44.6 ^o^C, beige: 32.6-46.2 ^o^C, white: 31.0-46.6 ^o^C. The range *T*_max_ - *T*_min_ and Δ*T* increased with increasing <*T*> (Fig. 2, Supplementary Tables S1-S4).

**Referee 3 wrote**: Moreover, remove figures and graphs only dealing with horse temperatures.

**Answer**: Figure 1 is important, because it shows typical thermograms of the black, brown, beige and white horses studied by us together with the back and belly areas where the surface temperature was averaged.

Figure 2 is also relevant, since it displays the minimum, maximum, average and standard deviation of the surface temperature of the back and belly of black, brown, beige and white horses measured by us with thermography under different illumination conditions.

Since we consider of importance Figures 1 and 2, we did not remove/delete them. Note that neither Referee 1, nor Referee 2 suggested the removal of these figures. All six figures of our paper are relevant for the informative presentation of our results.

**Referee 3 wrote**: b) Lines 145-149: these lines should be deleted, as results of this analysis are clear enough also without this heavy introduction.

**Answer**: The criticized sentences

Figure 5A displays the barrel’s surface temperature *T*_barrel_ versus air temperature *T*_air_ in field experiments 1-5. These two temperatures have a positive correlation, because at lower air temperatures the sunlit/shady air-filled (warm) barrels could not warm up as much as at higher air temperatures. In experiment 1 (when the weather was very warm) we used only air-filled sunlit warm barrels. Therefore, in this case low *T*_barrel_-values were missing at high air temperatures *T*_air_.

were shortened in the following way:

We can see in Fig. 5A that the barrel’s surface temperature *T*_barrel_ and the air temperature *T*_air_ correlate positively.

**Referee 3 wrote**: c) Lines 197-201: delete these lines. Not necessary and only making the discussion heavier.

**Answer**: As suggested, these sentences were deleted:

As far as we know, the time period for flies to change the temperature of their wing muscles has never been measured. This would be a very difficult task, because it would be problematic to measure the wing muscle temperature, and the speed of its warming up depends on the local temperature of the air and the thorax containing the muscles, the measurements of which would also be difficult.

**Referee 3 wrote**: d) Lines 209-218: delete these lines. They are an unnecessary and really unexplainable repetition of things already said elsewhere in the manuscript. They are Introduction, Materials and Methods, not a Discussion at all.

**Answer**: As suggested, the following paragraph and its references were deleted:

We studied experimentally the success rate of escape reactions of tabanid flies landed on black targets as a function of the surface temperature. Our aim was to test the prediction of Horváth *et al*. [17]. In order to avoid the many difficulties with freely moving horses, we used artificial targets mimicking the surface temperatures of darker (black, brown) and brighter (beige, white) ungulates, but of the same optical attractiveness. The latter condition is essential, because it is widely known that bright surfaces repel tabanids [5, 29, 30, 31, 32, 33, 34, 35]. Optically uniform shiny black barrels proved as an optimal choice: the surface temperature of these barrels could be changed in a wide range with changing the filling (air or cold water) and illumination (sunlit or shady), but the visual attractiveness to tabanids (radiance, colour, polarization) remained the same. Very similar methodology was used successfully in earlier field experiments [17].

29. Bracken GK, Hanec W, Thorsteinson AJ. The orientation behaviour of horse flies and deer flies (Tabanidae: Diptera). II. The role of some visual factors in the attractiveness of decoy silhouettes. Canadian Journal of Zoology 1962; 40: 685-695.

30. Granger CA. Trap design and color as factors in trapping the salt marsh greenhead fly. Journal of Economical Entomology 1970; 63: 1670-1673.

31. Browne SM, Bennett GF. Colour and shape as mediators of host-seeking responses of simuliids and tabanids (Diptera) in the Tantramar marshes, New Brunswick, Canada. Journal of Medical Entomology 1980; 17: 58-62.

32. Allan SA, Stoffolano JG. The effects of hue and intensity on visual attraction of adult Tabanus nigrovittatus (Diptera: Tabanidae). Journal of Medical Entomology 1986; 23: 83-91.

33. Sasaki H. Comparison of capturing tabanid flies (Diptera: Tabanidae) by five different color traps in the fields. Applied Entomology and Zoology 2001; 36: 515-519.

34. Krcmar S. Comparison of the efficiency of the olfactory and visual traps in the collection of horseflies (Diptera: Tabanidae). Entomologia Generalis 2013; 34: 261-267.

35. Horváth G, Blahó M, Egri Á, Lerner A. Applying polarization-based traps to insect control. In: G. Horváth (ed.) Polarized Light and Polarization Vision in Animal Sciences. 2014; Springer: Heidelberg, pp. 561-584.

**Referee 3 wrote**: e) Lines 265-278: the same as for point a). Reduce to 3 lines. This section is not for its own sake, but only functional to experiments on tabanids. Moreover, most part of relative results are very obvious. Again, shorten all the sections regarding horse temperatures and remove graphs, pics. They all make the paper very strenuous to read.

**Answer**: As suggested, the following subsection

Thermography of horses: On 6 July 2019 the distribution of the surface temperature (thermogram) of black, brown, beige and white horses was measured with an infrared camera (VarioCAM^®^, Jenoptik Laser Optik Systeme GmbH, Jena, Germany, nominal precision of ±1.5 ^o^C) under sunny and cloudy (when the sun was behind clouds) conditions. We recorded several infrared snapshots of a given horse and selected typical thermograms where the optical axis of the thermocamera was nearly perpendicular to the long axis of the horse. To determine the average surface temperature of horses over their back and belly, we used a custom-developed software. The validation and calibration of our thermocamera with a contact thermometer (GAO Digital Multitester EM392B 06554H, EverFlourish Europe Gmbh., Friedrichsthal, Germany, nominal precision of ±1 ^o^C) was described in detail in the Supporting Material of [17]. On each thermogram an extended area was selected on the back and belly of horses. Within these areas the mean temperature <*T*>, its standard deviation Δ*T*, minimum *T*_min_ and maximum *T*_max_ were determined with a custom-developed software in order to compare characteristic surface temperatures of 2 black, 2 brown, 2 beige and 2 white horses under sunlit and shady conditions on 46 thermograms.

was shortened in the following way:

Thermography of horses: On a warm day (6 July 2019) 46 thermograms of 2 black, 2 brown, 2 beige and 2 white horses were measured with an infrared camera (VarioCAM^®^, Jenoptik Laser Optik Systeme GmbH, Jena, Germany, nominal precision of ±1.5 ^o^C) under sunny and cloudy conditions. The validation and calibration of this thermocamera with a contact thermometer (GAO Digital Multitester EM392B 06554H, EverFlourish Europe Gmbh., Friedrichsthal, Germany, nominal precision of ±1 ^o^C) were described in the Supporting Material of [20].

**Referee 3 wrote**: f) Lines 290-292: not necessary. Delete;

g) Lines 295-296: not necessary. Delete.

**Answer**: As suggested, the following sentences were deleted:

We did not use barrels filled with warm/hot water, because the continuous production of warm/hot water throughout the day was technically impossible in the field.

The barrels were closed by black caps which hindered the outflow of air/water or the loss by evaporation.

**Referee 3 wrote**: h) Lines 326-329: shorten this explanation. It was not possible for technical problems. Most part of readers will be entomologists!!!.

**Answer**: The original sentence

For this purpose, thermography could not have been used, because (i) the thermocamera needed about two minutes for self-calibration after each switch on, whilst the next tabanids could land on the barrels, and (ii) on the recorded thermograms it would have been impossible to localize the exact landing sites of tabanids.

was shortened as suggested:

For this purpose, thermography was not possible to use because of technical problems.

**Referee 3 wrote**: i) Lines 342-345: Delete these lines. Authors just said that from an optical point of view the barrels were the same, this is enough.

**Answer**: As suggested, the following sentence was deleted.

Only tiny water droplets condensed from air humidity on the surface of the cold barrels could have resulted in some differences in reflection-polarization, but such condensation was never observed in our field experiments.

**Referee 3 wrote**: 2. Methodological problem: What reported at lines 89-92 is quite strange and needs to be explained by the Authors. In fact, while warm and cold barrels ranges of temperatures don't overlap, warm and 'cold horses' are highly overlapping. Moreover, while warm barrels are, for the most part, in the range of temperatures of 'warm horses', cold barrels temperatures are lower than those of 'colder horses', totally out of the range of temperature of 'cold horses'. This is very strange. Standing this situation, it seems that authors used cold barrels too cold, don't reflecting a real situation and hence possibly producing wrong results. This is really very relevant. The use of so cold barrels could invalidate the whole study. Authors should explain this choice and why, in their opinion, this is not affecting the validity of the work.

**Answer**: During our field experiments we could not keep the surface temperature of barrels within a desired range. That is why we chose the solution to use air-filled barrels to imitate warm hosts (which represents e.g. sunlit dark horses) and cold water-filled barrels with ice-packs to imitate cooler hosts. The temperature of barrels changed with time and was altered by the daily air temperature, so we regularly measured their surface temperature. Because of the experimental setup, the range of the barrels’ surface temperature was wider than the observed surface temperature range of the studied horses. During the linear and logistic regressions we used the barrel temperature as the independent variable and not the cold/warm barrel categorical variable. That is why cold/warm barrel categories were irrelevant at the regressions. Furthermore, we also applied the linear and logistic regressions as a funciton of the barrel temperature using the data within the 31 °C ≤ T ≤ 55 °C interval. This temperature range is what we have measured on living horses. The results of the linear and logistic regressions for the 31 °C ≤ T ≤ 55 °C interval and the wider 17 °C ≤ T ≤ 62 °C range were similar: the escape rate *e* increased with increasing *T*_barrel_. On the basis of these, the criticized sentences were moved from the Results to the revised Discussion in which we wrote the following:

During the field experiments all barrels were optically uniformly attractive to host-seeking horseflies. Our air-filled sunlit warm barrels (37-62 ^o^C) thermally imitated sunlit black horses (31-55 ^o^C), while our water-filled sunlit cold barrels (19-29 ^o^C) were cooler than sunlit brown, beige and white horses (31-47 ^o^C). Although the measured low escape successes in the case of our water-filled cold barrels were associated with temperatures that were lower than those on the studied horse bodies, the positive correlation between escape success *e* and surface temperature *T* (i.e. increasing *e* with increasing *T*) is also evident in the interval *T*_min_ = 31 ^o^C ≤ *T* ≤ *T*_max,BL_ = 55 ^o^C as clearly shown by the dotted curves of Figs. 4C and 6A. Thus, the results of our field experiments show that the escape success of tabanids depends on the host’s surface temperature *T*: the higher the *T*, the larger the escape success of horseflies. The average surface temperature of the studied sunlit black horses was between 48 and 55 °C. Almost all tabanids managed to escape in this temperature range.

**Referee 3 wrote**: 3. Contradictory Statements and Needed Explanations: a) Lines 66-68: if it is true, as Authors say in another part of the manuscript, that tabanids are not able to feel temperature of an object before landing on it, what stated in these lines is in contradiction with what Authors try to demonstrate in the paper. Black decoys used for catching horseflies would work not for their temperature, but for their colour.

**Answer**: There is here no any contradiction! On the one hand, to the revised Introduction we added the following:

The most effective tabanid traps use shiny black decoys [8, 9, 10, 11, 12, 13, 14, 15]. The so-called H-traps – composed of a bright tent with a shiny black sphere suspended below it – placed in sunny sites capture significantly more female tabanids than at shady sites [16]. The reason for this is that sunlit shiny dark targets reflect light at the Brewster’s angle with higher degrees of linear polarization *d* than shady ones [17, 18], and host-seeking female tabanids prefer high *d*-values independent of the direction of polarization [19]. Thus, shiny black decoys used to catch horseflies work for their colour and reflected degree of polarization, rather than for their temperature.

On the other hand, to the revised Discussion we added the following:

Since the thermoreceptors of tabanids (as usually in Diptera) are in their legs, antennae and mouthpart [28, 29, 30], they cannot sense remotely (e.g. by infrareceptors) the temperature of a target. They can sense precisely the surface temperature of a substrate/host only after a physical contact (landing). However, on the basis of the leg/antenna/mouth-sensed temperature of the boundary layer around a target, tabanids can decide whether the target’s surface is or is not warm enough for alighting [20].

**Referee 3 wrote**: b) Lines 129-144: the whole paragraph is very mixed up, difficult to read and understand. Furthermore, Authors don't explain some of the findings. Why there is a drop of escaped tabanids at very high temperatures? Zero at 62 °C? Why fewer tabanids landed on cold barrels if they feel temperature only after landing? Why the specification at lines 139-140? All these things are quite obscure for the reader. Authors should greatly simplify this section, explain these points and report only the relevant results.

**Answer**: Note that the missed explanations were partly involved in the original version of this criticized paragraph:

Referee 3 asked: Why there is a drop of escaped tabanids at very high temperatures? Zero at 62 °C? Why the specification at lines 139-140 (Note again that the waiting time until a catch attempt was 10 seconds after a tabanid landed on a barrel.)?

The answer is: At higher surface temperatures more tabanids landed on the barrels, but because of the hot surface many of them flew away before the capturing attempt was performed in 10 seconds after landing (see Materials and Methods and Discussion). Therefore, the landing events shorter than 10 seconds were not registered, thus *N*_e_ dropped with increasing *T*.

Referee 3 asked: Why fewer tabanids landed on cold barrels if they feel temperature only after landing?

The answer is: The explanation of the drop of *N*_e_ at lower surface temperatures is that (i) less tabanids landed on colder surfaces, because tabanids flying through the cold boundary layer around the cold barrels could sense the lower surface temperature by their thermoreceptors (in the legs, antennae, mouthpart) and decided less frequently to alight, as Horváth *et al*. [20] showed, and (ii) the escape rate is lower at colder surfaces (see Fig. 4C).

As requested by Referee 3, this paragraph was revised in the following way:

According to Fig. 4A, the number *N*_e_ of escaped tabanids landed on barrels has a maximum at around 41 ^o^C, drops to zero at 17 ^o^C and decreases almost to zero at 62 ^o^C. The explanation of the drop of *N*_e_ at lower surface temperatures is that (i) less tabanids landed on colder surfaces, because tabanids flying through the cold boundary layer around the cold barrels could sense the lower surface temperature by their thermoreceptors (in the legs, antennae, mouthpart) and decided less frequently to alight, as Horváth *et al*. [20] showed, and (ii) the escape rate is lower at colder surfaces (see Fig. 4C). At higher surface temperatures more tabanids landed on the barrels, but because of the hot surface many of them flew away before the capturing attempt was performed in 10 seconds after landing (see Materials and Methods and Discussion). Therefore, the landing events shorter than 10 seconds were not registered, thus *N*_e_ droped with increasing *T*. In Fig. 4B, the number *N*_c_ of captured tabanids landed on barrels exhibits a clear decreasing trend with increasing *T*_barrel_ for both temperature intervals of 17 ^o^C ≤ *T* ≤ 62 ^o^C and *T*_min_ = 31 ^o^C ≤ *T* ≤ *T*_max,BL_ = 55 ^o^C. In Fig. 4C the increasing tendency of the normalized escape succes *e* = *N*_e_/(*N*_e_ + *N*_c_) with increasing barrel’s surface temperature *T*_barrel_ is clear. Figure 4C also illustrates that the systematic increase of the escape success *e* is also present in the temperature range *T*_min_ = 31 ^o^C ≤ *T* ≤ *T*_max,BL_ = 55 ^o^C that is typical for the surface of horses.

**Referee 3 wrote**: c) Line 294: Why 10 seconds? In Materials and Methods Authors should explain this choice.

**Answer**: In the revised manuscript we wrote:

Field experiments

The waiting time until a catch attempt was 10 seconds (measured with a stopwatch) after a tabanid landed on a barrel (the choice of this optimal 10-second value is explained in the Discussion).

Results

Note that the waiting time until a catch attempt was 10 seconds after a tabanid landed on a barrel (see Materials and Methods and Discussion).

Discussion

In our field experiments the investigator waited 10 seconds before he tried to capture a tabanid landed on a barrel. This 10-second period turned out to be optimal: it was neither too short, nor too long. Within a period shorter than 10 s the flight muscles of landed tabanids could not warm up or cool down to the surface temperature of barrels. On the other hand, when a landed tabanid recognizes that the barrel is not a host animal, it flies away after a certain period. If the waiting period of counting of landed tabanids were too long, then many landing events would not be counted/registered. The air temperature did not correlate with the escape rate (Fig. 5C), therefore 10 s was long enough to warm the wing muscles of flies landed on the barrel. On the other hand, if a tabanid lands on a sunlit dark (warm) surface, then it does not cool down, contrary to a bright (colder) surface where its wing muscles can cool down. It is pertinent to suppose that during flight the wing muscles are appropriately warm. Furthermore, according to Heinrich [33], many Dipteran species (including true flies and also tsetse flies) can be heterothermic, or generate certain amount of heat which can then be used to improve their performance. However, as far as we know, practically nothing is known about the thermoregulation in horseflies. Thus, without knowing the horsefly thermal physiology, we restricted our study to the correlation between (air/barrel) temperature and escape success with a waiting period of 10 seconds before the experimenter tried to capture a tabanid landed on a barrel.

**Referee 3 wrote**: d) Lines 331-333: it is not clear how Authors prevented the simultaneous landing of two or more tabanids on barrels. They give it for granted, but it is not.

**Answer**: We did not need to prevent the simultaneous landing of two or more tabanids on the barrels, because in the barrels’ vicinity there were no so many flying tabanids. In the revised manuscript we wrote:

In the vicinity of the barrels there were no many flying tabanids. Threfore, only one tabanid landed on one of the two (warm or cold) barrels at a given time, thus the experimenter did not have to split his attention between two or more landed tabanids.

**Referee 3 wrote**: At Line 62, 'spp.' not in italics.

**Answer**: Italic ’ssp.’ was corrected to normal ‘spp.’.

**Referee 3 wrote**: At line 84 'would also good for' should be changed in 'would also be good for'.

**Answer**: As suggested by Referee 1, this sentence was revised as follows:

The results of our field experiments presented here corroborated the mentioned prediction which explains why blood-seeking horseflies prefer sunlit dark (warmer) host animals.

**Referee 3 wrote**: In conclusion, the paper by Horváth et al. could deserve publication on PLOS ONE only after major changes regarding its length and some logic inconsistency that Authors should modify or better explain.

**Answer**: The changes regarding length and some logic inconsistency suggested by Referee 3 were done in our revised manuscript.
