# Supplementary material for: Why do biting horseflies prefer warmer hosts? tabanids can escape easier from warmer targets
Source: PLoS One. 2020 May 13;15(5):e0233038. doi: 10.1371/journal.pone.0233038 (PMC7219777; doi:10.1371/journal.pone.0233038)
Supplement: Attachment — Submitted filename: +PONE-D-20-04024-R2_response.doc [file pone.0233038.s021.doc]

**Point-by-Point Response to the Comments of the Academic Editor**

We thank the Academic Editor for her new comments. We have revised our paper on the basis of the concerns and comments raised in the annotated pdf. All changes done with respect to this annotated pdf are marked with violet. Below one can read our Point-by-Point Response to these points.

**Editor wrote**: Given that PLoS One does not provide any editorial service after acceptance at minimum the authors should address the grammatical concerns raised in the annotated pdf. These include

1. the replacement of the term 'shady' with 'shaded' as well as

2. the inclusion of 'that' before 'landed' on many occasions

3. In addition, the authors should use their abbreviations (i.e. Tair, Tbarrel, Tbarrel-Tair) consistently after once briefly introducing them at first mention.

**Answer**: In our 2nd revised manuscript

- all grammatical errors were corrected,

- all statistical results were reported in order X2, df and p,

- any kind of interpretation was moved to the Discussion section,

- where requested, new references were added the concerned statement,

- in the References, doi's were added whereever possible.

**Editor wrote**: However, there should be some empirical statement here why this time window was chosen.

**Answer**: In our 2nd revised manuscript we wrote:

The time allowed to elapse before a capture attempt was 10 seconds (measured with a stopwatch) after a tabanid landed on a barrel. This 10-second period turned out to be optimal: it was neither too short, nor too long (a more detailed explanation of the choice of this optimal 10-second value can be read in the Discussion).

**Editor wrote**: Please elaborate on the technical difficulties that prevented the use of thermography.

**Answer**: In our 2nd revised manuscript we wrote:

For this part of the study, the use of thermography was not possible, because (i) the thermocamera needed about two minutes for self-calibration after each switch on, whilst the next tabanids could land on the barrels, and (ii) on the recorded thermograms it would have been impossible to localize the exact landing sites of tabanids. The experimenter was the same person throughout all experiments, who had practised the capture of tabanids during a pilot experiment. Due to the low number of flying tabanids in the vicinity of the barrels, only single tabanids landed on the barrels at any given time. Thus, the experimenter’s attention could focus entirely on one fly at a time.

**Editor wrote**: Please clearly indicate dependent and independent variables in these regression analyses.

**Answer**: In our 2nd revised manuscript we wrote:

Linear regressions were applied to find a trend of the escape success of horseflies as a function of *T*_barrel_, *T*_air_ and *T*_barrel_ - *T*_air_. The independent variables were *T*_barrel_, *T*_air_ and *T*_barrel_ - *T*_air_, while the dependent variable was the normalized escape success *e* = *N*_e_/(*N*_e_ + *N*_c_, where *N*_e_ is the number of escaped tabanids and *N*_c_ is the number of captured horseflies.
